# Integrating deep mutational scanning and low-throughput mutagenesis data to predict the impact of amino acid variants

**DOI:** 10.1093/gigascience/giad073

**Published:** 2023-09-18

**Authors:** Yunfan Fu, Justin Bedő, Anthony T Papenfuss, Alan F Rubin

**Affiliations:** The Walter and Eliza Hall Institute of Medical Research, Bioinformatics Division, 1G Royal Pde, Parkville, Victoria 3052, Australia; The University of Melbourne, Department of Medical Biology, Parkville, Victoria 3010, Australia; The Walter and Eliza Hall Institute of Medical Research, Bioinformatics Division, 1G Royal Pde, Parkville, Victoria 3052, Australia; The University of Melbourne, Department of Medical Biology, Parkville, Victoria 3010, Australia; The Walter and Eliza Hall Institute of Medical Research, Bioinformatics Division, 1G Royal Pde, Parkville, Victoria 3052, Australia; The University of Melbourne, Department of Medical Biology, Parkville, Victoria 3010, Australia; Peter MacCallum Cancer Centre, Melbourne, Victoria 3000, Australia; The Walter and Eliza Hall Institute of Medical Research, Bioinformatics Division, 1G Royal Pde, Parkville, Victoria 3052, Australia; The University of Melbourne, Department of Medical Biology, Parkville, Victoria 3010, Australia

**Keywords:** deep mutational scanning, alanine scanning, machine learning, predictor

## Abstract

**Background:**

Evaluating the impact of amino acid variants has been a critical challenge for studying protein function and interpreting genomic data. High-throughput experimental methods like deep mutational scanning (DMS) can measure the effect of large numbers of variants in a target protein, but because DMS studies have not been performed on all proteins, researchers also model DMS data computationally to estimate variant impacts by predictors.

**Results:**

In this study, we extended a linear regression-based predictor to explore whether incorporating data from alanine scanning (AS), a widely used low-throughput mutagenesis method, would improve prediction results. To evaluate our model, we collected 146 AS datasets, mapping to 54 DMS datasets across 22 distinct proteins.

**Conclusions:**

We show that improved model performance depends on the compatibility of the DMS and AS assays, and the scale of improvement is closely related to the correlation between DMS and AS results.

## Introduction

Deep mutational scanning (DMS) is a functional genomics method that can experimentally measure the impact of many thousands of protein variants by combining high-throughput sequencing with a functional assay [1]. In a typical DMS, a complementary DNA library of genetic variants of a target gene is generated, containing all possible single amino acid substitutions. This variant library is then expressed in a functional assay system where the DMS variants can be selected based on their properties. The change in variant frequency in the pre- and postselection populations is determined by high-throughput sequencing, which is then used to calculate a multiplexed functional score that captures the variant's impact [2–4]. The versatility of DMS assays makes it possible to measure variant impact on a wide range of protein properties, including protein binding affinity [5, 6], protein abundance [7–9], enzyme activity [10, 11], and cell survival [12–14]. So far, hundreds of DMS studies covering tens of thousands of nucleotides have been published [15], and experiments targeting over a hundred additional genes are under way according to MaveRegistry [16].

Computational studies have used DMS data to build predictive models of variant impact. These predictors use supervised or semi-supervised learning models trained on experimental DMS data and various protein features to make predictions [17–23]. Envision is one such method that used protein structural, physicochemical, and evolutionary features to predict variant effect scores and was trained on DMS data from 8 proteins using gradient boosting [17]. Another method, DeMaSk, predicted DMS scores by combining 2 evolutionary features (protein positional conservation and variant homologous frequency) with a DMS substitution matrix and was trained on data from 17 proteins using a linear model [19]. Deep learning algorithms have also been applied to build protein fitness predictors [18, 20], which are usually based only on variant sequences. These variant effect predictors can also be benchmarked using DMS experimental results and assist in the interpretation of experimental data [20, 24, 25].

Low-throughput mutagenesis experiments that measure tens of variants at a time have also been used extensively to study diverse protein properties, including substrate binding affinity [26, 27], protein stability [28, 29], and protein-specific activities [30, 31]. Alanine scanning (AS) is a widely used low-throughput mutagenesis method [32, 33], and AS data are available for many proteins. In this method, each targeted protein residue is substituted with alanine, and the impacts of these variants are measured by a functional assay [34]. AS experiments are typically used to identify functional hot spots or critical residues in the target protein [35, 36] and have been used as a source of independent validation for DMS studies [31, 37–39].

**Figure 1:**
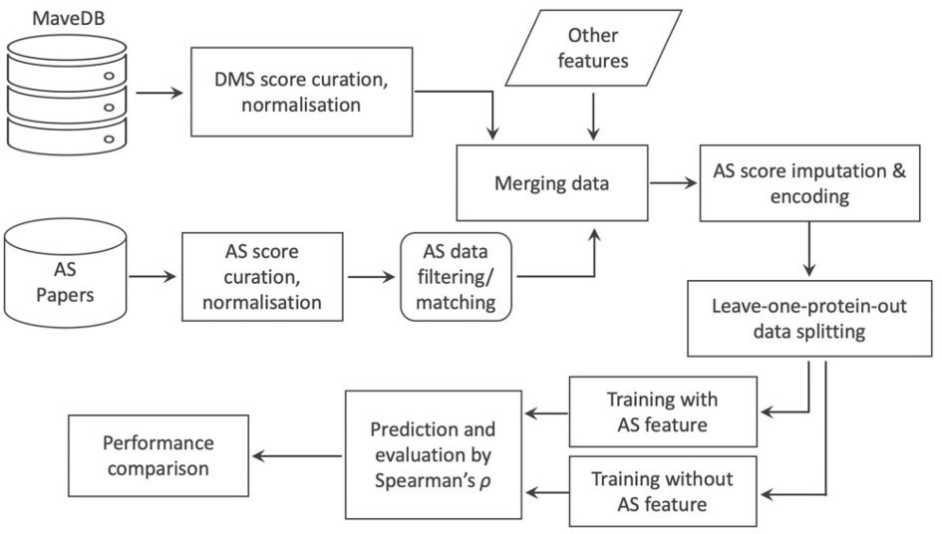
Workflow for model training and testing. DMS and AS datasets are collected from online resources and are normalized. DMS and AS datasets targeting the same protein are then matched, filtered, and merged. Two predictors are constructed and tested: the first uses DMS data, AS data, and other protein features, and the second uses only DMS data and the same other protein features.

In this study, we explore whether a predictive model can be improved by incorporating low-throughput mutagenesis data (Fig. 1). We find that AS data can increase prediction accuracy and that the improvement is related to the similarity of the functional assays and the correlation of DMS and AS results.

## Methods

### DMS data collection

DMS data were downloaded from MaveDB [40, 41], which were then filtered and curated. DMS experiments targeting antibody and virus proteins were removed because of their potentially unique functionality. We retrieved the UniProt accession ID of target proteins by searching the protein names or sequences in UniProt [42], and proteins lacking available UniProt ID were also excluded. Datasets that are computationally processed or their wild-type-like and nonsense-like scores (see Normalization) cannot be identified were also filtered out (Supplementary Table S1). All missense variants with only a single amino acid substitution were curated from the DMS studies for our analysis. A total of 130 DMS experiments from 53 studies [5, 6, 9–14, 24, 31, 37–39, 43–80] were collected for our analysis.

### Collection of AS data and other features

The following process was used to search for candidate AS studies. Papers were identified by searching on PubMed and Google Scholar for the “alanine scan” or “alanine scanning” together with the name of candidate proteins. While searching in Google Scholar, we included the protein's UniProt ID rather than molecule name as the search term to reduce false positives. Appropriate AS data were collected from the search results. Western blot results were transformed to values by ImageJ if it was the only experimental data available in the study. A total 146 AS experiments were collected from 45 distinct studies [26–28, 30, 31, 81–86, 70, 87–119].

Protein features of Shannon entropy and the logarithm of variant amino acid frequency were downloaded from the DeMaSk online toolkit [19]. The substitution score matrix feature was calculated from the mean of training DMS scores for each of the 380 possible amino acid substitutions before each iteration of cross-validation.

### Normalization

DMS and AS datasets were normalized to a common scale using the following approach adapted from previous studies [17, 120]. Let *D* denote a protein study measuring scores $s_i^D$ for a single variant *i*, $s_{wt}^D$ denote the scores for wild-type, and $s_{non}^D$ represent the score for nonsense-like variants. The normalized scores $s_i^{^{\prime}D}$ are given by


\begin{eqnarray*}
s_i^{^{\prime}D}\colon = \frac{{s_i^D - s_{wt}^D}}{{s_{wt}^D - s_{non}^D}} + 1
\end{eqnarray*}

Wild-type scores were directly identified from the paper or the median score of synonymous variants. For DMS data, since not all DMS studies report the score of nonsense variants, we defined the nonsense-like scores as the median DMS scores for the 1% missense variants with the strongest loss of function for each dataset. For AS data, nonsense-like scores were defined according to the paper or by using the extreme values (Supplementary Table S1).

### AS data filtering and matching

AS data subsets were filtered/matched according to either assay compatibility or score correlation. For assay compatibility filtering, we first categorized each DMS or AS assay by the protein property or function using the following assay types: binding affinity, enzyme activity, protein abundance, cell survival, pathogen infection, drug response, ability to perform a novel function, or other protein-specific activities (e.g., transcription activity for transcription factors) (Supplementary Table S1). The DMS/AS assay pairs were then classified into 3 levels of compatibility based on these categories (Supplementary Fig. S2). For each DMS dataset, we first tried to use only AS data with high assay compatibility for further modeling, removing AS data of medium and low assay compatibility. We then also tried to model with AS data of both high and medium assay compatibility.

For score correlation matching, Spearman's correlation (*ρ*) is calculated between alanine substitution scores in each pair of AS and DMS data. To avoid influence from the size of AS datasets, we estimated the *ρ* value with the empirical copula, which is related to the standard estimator by a factor of (*n –* 1)/(*n* + 1) [121, 122]:


\begin{eqnarray*}
{\rho }_r\colon = \rho \times \frac{{n - 1}}{{n + 1}}
\end{eqnarray*}

where ${\rho }_r$ is the regularized correlation coefficient, and *n* is the number of alanine substitutions used for correlation calculation. For each DMS dataset, the AS result with the highest ${\rho }_r$ was picked for modeling.

### AS data preprocessing

AS data were preprocessed prior to modeling. For variants without available (filtered/matched) AS data, their AS scores were imputed with the mean value of all available AS scores across all studies. Then the AS data were encoded by the wild-type and variant amino acid type with one-hot encoding. For each variant, the AS feature is expanded with 2 one-hot vectors. Each of the vectors has 19 zeros and 1 nonzero value that was the AS score, with the location of the nonzero value indicating the wild-type or variant amino acid type.

### Training and evaluation of DMS score predictor

To build the predictors, we performed linear regression using the function sklearn.linear_model.LinearRegression from scikit-learn [123]. Training and validation data were separated with leave-one-protein-out cross-validation. In this process, data from 1 protein were withheld for subsequent validation, and the rest were used for training. This process was iterated over all proteins in the data. Variants were inversely weighted during the training process by the number of measurements available, thus compensating for some regions having greater coverage with DMS and AS assays. Predictors were trained on protein features, DMS data, and (optionally) AS data using 4 different filtering or matching strategies: (i) all DMS/AS data, (ii) compatibility-filtered DMS/AS data, (iii) correlation-matched DMS/AS data, and (iv) a control, constructed using DMS data only.

In the evaluation process, let *V* be protein variants assayed by both DMS study *D* and AS study *A*. Variant scores are predicted by the previously mentioned predictors either using AS data ($\hat{s}_V^A$) or not (${\hat{s}}_V$). Spearman's correlation (*ρ*) was calculated between the DMS scores $s_V^D$ and each set of predicted scores. The difference of *ρ* was used to evaluate the performance change ($\Delta {\rho }_V$).


\begin{eqnarray*}
\rho _V^A = {\mathrm{Spearman{^{\prime}}s\,\, correlation}}\left( {\hat{s}_V^A,s_V^D} \right) \end{eqnarray*}

\begin{eqnarray*}
{\rho }_V = {\mathrm{Spearman{^{\prime}}s\ correlation}}\left( {{{\hat{s}}}_V,s_V^D} \right) \end{eqnarray*}

\begin{eqnarray*}
\Delta {\rho }_V = \rho _V^A - {\rho }_V
\end{eqnarray*}

To evaluate, we iterated over variants from each pair of DMS/AS studies. Results were dropped for variants *V* with only 1 protein residue available during analysis and visualization. Model performance was compared using the following statistical tests. Results in Fig. 5 and Supplementary Fig. S5 were tested with Welch's test, and results in Supplementary Figs. S4 and S6 were tested with paired *t*-tests. The *P* values were jointly corrected using the Holm–Šidák method. The 95% confidence interval of median values was calculated by Gaussian-based asymptotic approximation [124].

### Prediction with other variant effect predictors

For PROVEAN [125] and SIFT [126], prediction results on target variants were directly downloaded from the precalculated database for PROVEAN. For PolyPhen-2 [127] and GEMME [128], variant scores were computed through their online toolkits, using the default settings. ESM-1v [129] was set up locally and run according to its examples and documentations. EVE [130] results were collected from their precalculated database and a benchmarking study [131].

## Results

### Overview of DMS and AS data

**Figure 2:**
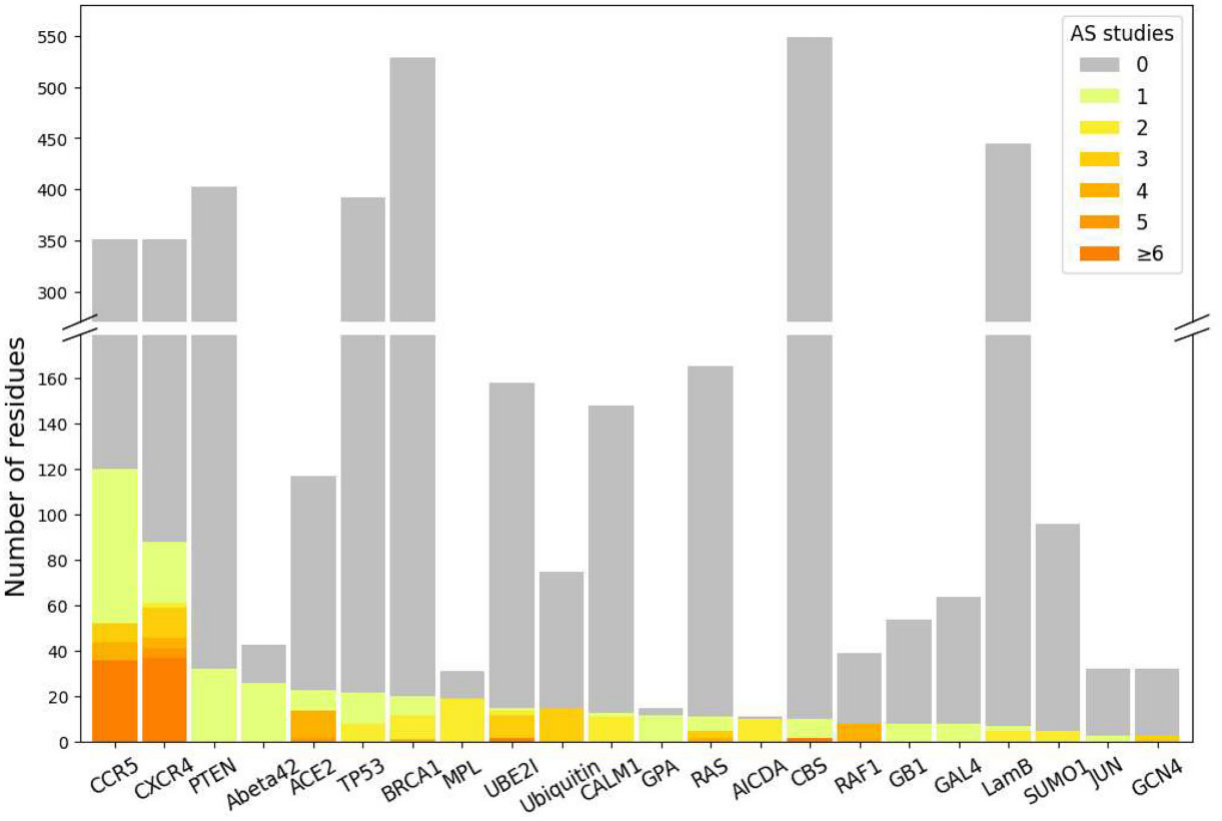
DMS data generally cover more protein residues than AS data. Each bar shows the number of residues assayed by DMS studies on given target proteins. Color indicates the number of AS studies available for the DMS-tested residues.

To build the predictive model, 130 DMS datasets were collected from MaveDB [40, 41] (Supplementary Table S1). We searched the literature and found 146 AS datasets targeting the same proteins as 54 of the DMS datasets. In total, we obtained both DMS and AS data for 22 different proteins: 17 human proteins, 3 yeast proteins, and 2 bacterial proteins. Most DMS experiments were highly complete, with a mean coverage of 95.0% of all possible single amino acid substitutions assayed in the target region, comprising 373,219 total protein variant measurements. AS data were only available on a small number of protein residues (Fig. 2), and we were able to curate 1,480 alanine substitution scores from the 146 studies. Variant scores from collected DMS and AS studies were linearly normalized to a common scale (see Methods) to make them comparable across datasets (Supplementary Fig. S1).

### The correlation of DMS and AS scores is related to assay compatibility

**Figure 3:**
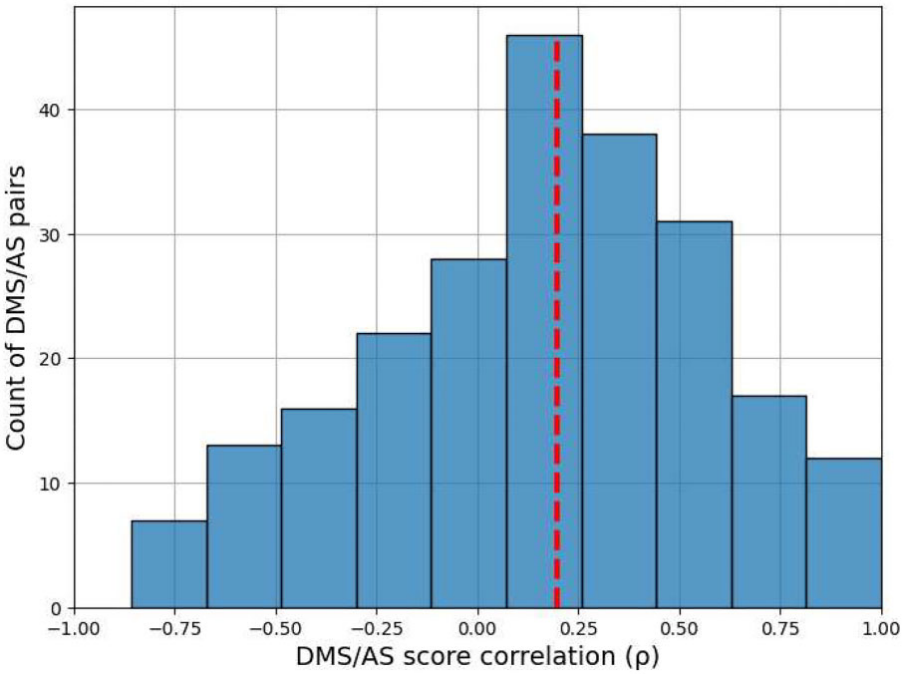
Correlation between DMS and AS data shows substantial variation. We calculated Spearman's *ρ* between alanine substitution scores in each pair of AS and DMS data. The results for pairs with fewer than 3 alanine substitutions are not shown. The red dashed line shows the median *ρ*.

To evaluate the similarity of AS and DMS scores, we calculated Spearman's correlation (*ρ*) between the AS scores and DMS scores for the same alanine substitutions. Since each protein may have results from several AS and DMS experiments, we calculated *ρ* between each possible pair. The median *ρ* over DMS and AS data (DMS/AS) pairs was 0.2, indicating that the experimental scores were poorly correlated overall (Fig. 3).

**Figure 4:**
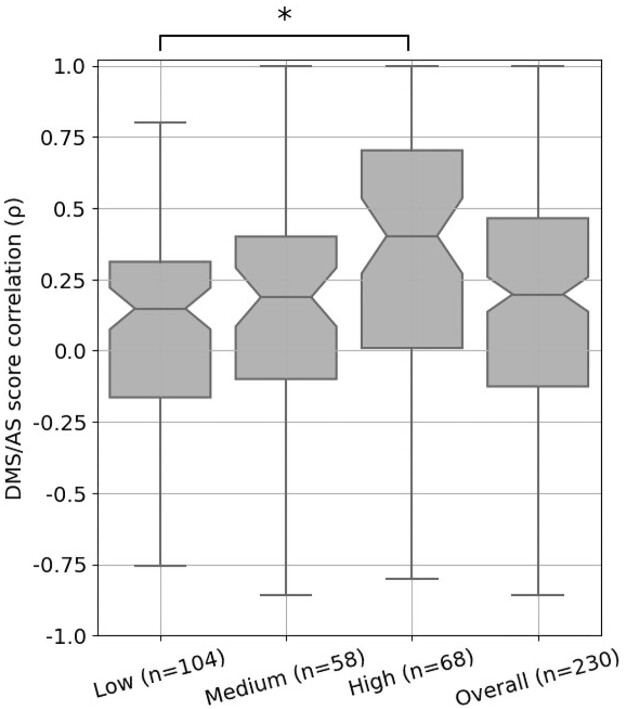
DMS and AS data pairs with high assay compatibility show a higher score correlation. Each box shows the Spearman's *ρ* between DMS and AS data pairs for each level of assay compatibility or overall. The correlation coefficients were calculated between alanine substitution scores in each pair of AS and DMS datasets. Results for pairs with fewer than 3 alanine substitutions were removed. *P* values calculated using Welch's test and corrected using Holm–Šidák, *P < 0.05; notches show 95% confidence interval around median, and whiskers show the full value range.

We then considered if differences between AS and DMS assay designs might contribute to this low agreement between scores. To explore this, we developed a decision tree (Supplementary Fig. S2) to classify whether DMS/AS pairs had low, medium, or high assay compatibility, which we defined as a similarity measurement of the functional assays performed. For example, the DMS assay measuring the binding affinity of a cell surface protein, CXCR4, to its natural ligand [43] has high compatibility with the AS experiment also measuring this ligand binding but has low compatibility with the study on CXCR4’s ability to facilitate virus infection [81]. A full assay compatibility table can be found in Supplementary Table S1 with the compatibility classifications and justification for each pair. We then compared DMS and AS score correlation for each compatibility class and found that score correlations were closely related to assay compatibility. Data from low-compatibility assays had a median correlation of 0.15, rising to 0.19 for medium-compatibility assays and 0.40 for high-compatibility assays (Fig. 4). This trend of increased correlation for high-compatibility assay pairs holds across secondary structures (Supplementary Table S4). This link between assay compatibility and score correlation indicates that our decision tree approach was able to capture the similarity between assay systems.

### Compatible AS data improve DMS score prediction accuracy

To test if incorporating AS data into DMS score models would improve prediction accuracy, we decided to build a new model based on DeMaSk [19]. We chose DeMaSk because it showed better performance compared to similar methods and was straightforward to modify. The published DeMaSk model predicts DMS scores using protein positional conservation, variant homologous frequency, and substitution score matrix, and we incorporated AS data as an additional feature. Our new predictor was modeled with all 130 DMS we collected, and we applied a leave-one-protein-out cross-validation approach to training and testing, avoiding information leakage for variants of the same protein target [17]. Prediction performance was evaluated using the Spearman's correlation (*ρ*) between the experimentally derived DMS scores and the predicted scores for each pair of DMS and AS studies. The performance of our DMS/AS model was compared with a model trained only on DMS data, equivalent to retrained DeMaSk (Supplementary Fig. S3), by calculating the change of prediction *ρ* (see Methods).

**Figure 5:**
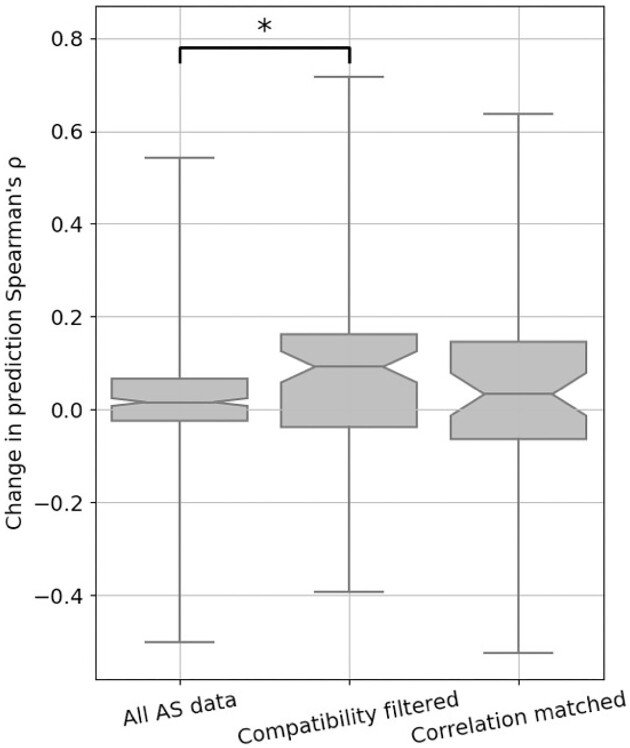
Performance of variant impact prediction is improved using AS data with high assay compatibility. The change in prediction *ρ* achieved by including the AS data feature for each DMS and AS data pair is shown as box plots. A higher value represents higher prediction accuracy achieved for using AS data. Different approaches to filtering/matching the data are shown on the x-axis: “All AS data” used all available data; “Compatibility filtered” used only data of high assay compatibility; “Correlation matched” used only data with the highest regularized correlation for each DMS dataset. Results for data pairs with only 1 residue are not shown. *P* values were calculated using Welch's test and jointly corrected using Holm–Šidák (Methods), *P < 0.05. Notches show the 95% confidence interval around the median, and whiskers show the full value range.

We trained our model with either all or a subset of AS data we collected (Fig. 5, Supplementary Table S5). We first integrated all 146 AS data collected for training and evaluation but observed only a modest improvement of prediction *ρ* (Fig. 5, left box, and Supplementary Fig. S4). We then retrained and evaluated our model on filtered AS data with only high-compatibility assays and observed a median increase in prediction Spearman's *ρ* of 0.1 compared to the results with no AS data (Fig. 5, middle box, and Supplementary Fig. S4). However, training with both high- and medium-compatibility pairs reduced the performance improvement (Supplementary Fig. S5). These results indicate that medium- and low-compatibility pairs might provide inconsistent training data, degrading model performance. We also evaluated the impact of including high-compatibility AS data in an alternative model based on Envison [17, 132] and found similar results (Supplementary Fig. S6 and Supplementary Information). To differentiate between high assay compatibility and high DMS/AS score correlation, we trained the model using the most highly correlated AS result for each DMS dataset (see Methods). Although the upper quartile was high, the median performance change of this predictor was lower than the high assay compatibility model, suggesting that matching with the highest score correlation alone is insufficient (Fig. 5, right box). However, when applying a stricter threshold, the correlation matched models still show limited improvement (Supplementary Fig. S7). Additionally, to ensure the models performance is not biased by pseudo-replication of multiple datasets, we averaged DMS and AS scores that were part of the same study and type of assay and saw similar results (Supplementary Fig. S8).

**Figure 6:**
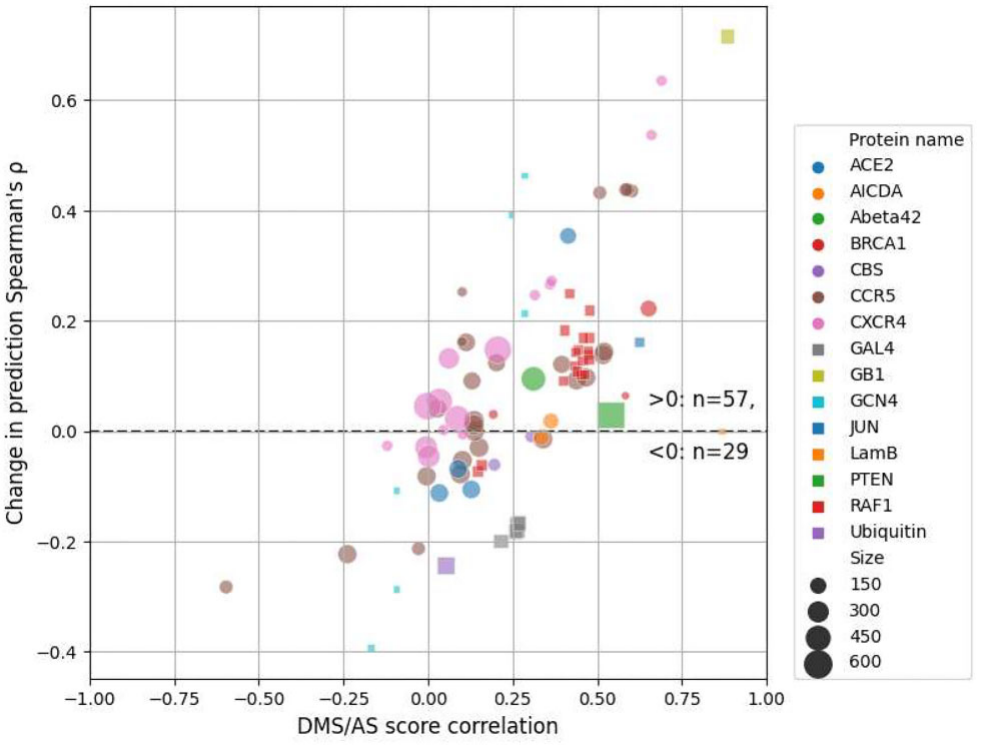
Prediction performance change is related to DMS and AS score correlation. Each dot represents a filtered DMS/AS data pair of high assay compatibility. The vertical axis shows the change of prediction *ρ* by using AS data (larger means higher performance achieved by using AS data). The horizontal axis shows the DMS/AS score correlation for *all* variants on the matched residues rather than just alanine substitutions. The colors and shapes of the dots correspond to the target protein, and size indicates the number of variants in each data pair. Results for data pairs with only 1 residue are not shown.

Our compatibility-filtered predictor shows improved prediction accuracy for these regions compared to not only the baseline model but other widely used predictors as well (Supplementary Fig. S9). To further explore the higher performance of this compatibility-filtered predictor, we examined the relationship between prediction *ρ* change and score correlation for each high-compatibility DMS/AS pair (Fig. 6). For most pairs, prediction performance was improved by using AS data, and the scale of improvement was also related to the score correlation. This relationship could also be observed for multiple DMS/AS pairs from an individual protein, such as CXCR4 and CCR5. We saw the same trend in the predictor trained with all DMS/AS pairs but noted that the performance even of highly correlated pairs was worse, likely due to the influence of low-compatibility training data on the model (Supplementary Fig. S10).

We also explored the consequences of the sparsity of AS data on our model in 3 ways: (i) by training only with variants that have AS data available (Supplementary Fig. S11), (ii) by using a boosting approach that focuses only on residues with AS data (Supplementary Fig. S12 and Supplementary Information), and (iii) by using complete alanine substitution information from DMS as the AS feature (Supplementary Fig. S13 and Supplementary Information). The first approach gave lower absolute prediction performance, presumably because the model was underfitted due to the small number of variants. The last 2 approaches performed very similarly to the primary model constructed using high-compatibility DMS/AS data and simple mean score imputation.

**Figure 7:**
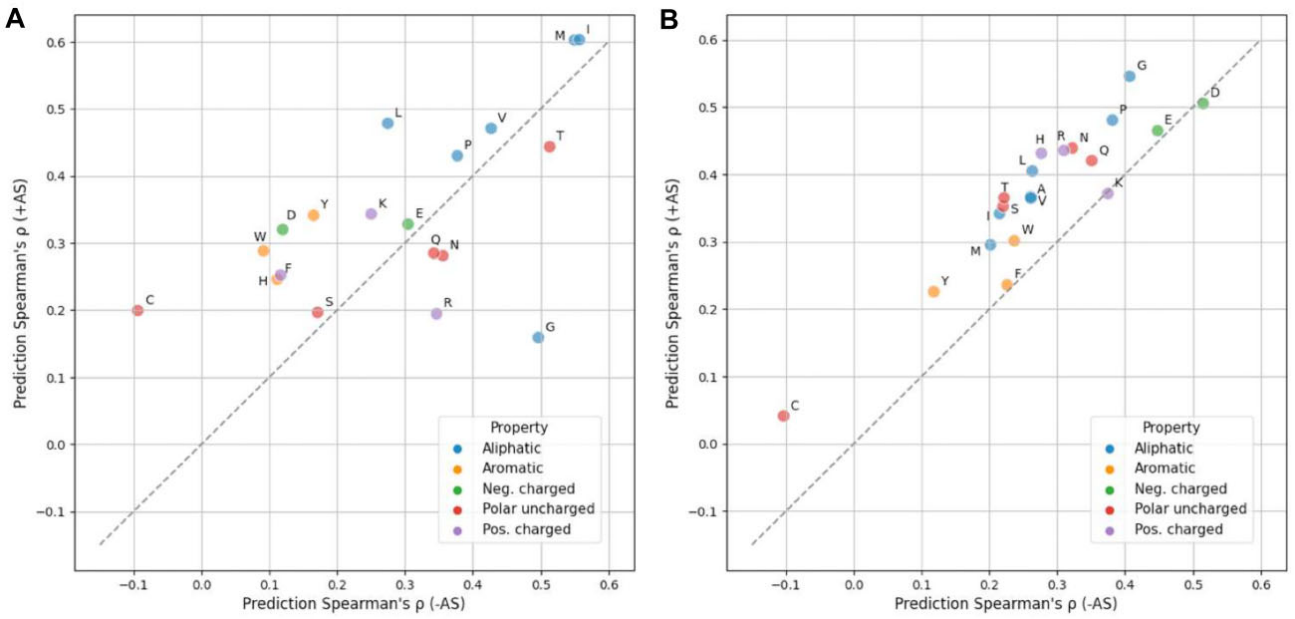
Model performance is generally improved for each wild-type and variant amino acid. Prediction Spearman's *ρ* when using (y-axis) or not using (x-axis) AS data on each wild-type (A) or variant (B) amino acid is shown in the scatterplots. The results are colored according to the property of each amino acid type. Alanine (A) result is not applicable in the first figure since alanine scanning data are always missing when the wild-type is alanine itself. Absolute count for each amino acid can be found in Supplementary Fig. S14. Neg., negatively; Pos., positively.

To test the influence of amino acids on our predictor, we grouped the prediction results by either wild-type or variant amino acid and calculated the prediction improvement when AS data were included (Fig. 7). We found that 14 of 19 wild-type amino acids performed better with the addition of AS data, with cysteine showing the largest improvement and performing worst in the model lacking AS data. Eighteen of 20 variant amino acids benefited from the inclusion of AS data, with marginal performance decrease on lysine and aspartic acid (|Δ*ρ*| < 0.01) (Fig. 7). We also noticed that variants to alanine are not most improved, but we observed an overall trend showing higher improvement for amino acids that are physiochemically similar to alanine (Supplementary Fig. S15).

## Discussion

In this study, we integrated AS data into DMS score prediction, leading to modest improvements in the accuracy of variant score prediction. We also explored the impact of the diversity of protein properties measured by DMS and AS. Filtering DMS and AS data based on our manual classification of assay type compatibility led to improved prediction performance.

A potential shortcoming of our current approach is that AS data were available for only a small proportion of the DMS data. Although most recent DMS studies can analyze variants of the whole protein, most AS experiments only cover a handful of residues in the target protein, leaving missing AS scores for the vast majority of residues. We explored this here and found that alternative methods for addressing the sparsity of AS data did not improve or degrade performance, but we anticipate further improved prediction accuracy if the low completeness and unevenness of AS data are appropriately handled before modeling.

In this study, we identified the importance of DMS/AS assay compatibility as a crucial factor for improving prediction accuracy. An issue with using this concept is that it further shrinks already sparse data. It also fails to take advantage of the fact that even for low-compatible assays, some fundamental information like protein abundance can still be mutually captured. Instead of hard filtering, proper implementation of this underlying information may facilitate variant impact prediction in the future. Nonetheless, filtering on assay compatibility still leads to performance improvement. We also briefly explored whether the consistency of DMS and AS scores can be considered more directly by matching the best-correlated AS data for each DMS dataset. Consistency is partially driven by assay compatibility but also reflects other features of the data, such as bias and noise.

The concepts of compatibility and data quality are also relevant to training any DMS-based predictors. DMS assays have been developed to measure variant impacts to distinct protein properties, and a variant can behave similarly to wild-type when measured by one assay yet show altered protein properties in other assay results, which are frequently found in regions with specific biochemical functions [25, 133–137]. With more experimental assays to be applied, the diverse measurements may impede the progress of future DMS-based predictors unless this assay effect is properly addressed, for example, by building assay-specific predictors. Measurement error is another source of DMS data heterogeneity that potentially affects the model performance. In our current study, DMS scores of protein variants are weighted equally while training. Adjustable weighting can be applied in future studies to adapt the distinct experimental error between individual variants and datasets, reducing the influence of low-confident data.

In summary, we conclude that the careful inclusion of low-throughput mutagenesis data improves the prediction of DMS scores, and the approaches described here can potentially be applied to other prediction methods.

## Availability of Supporting Source Code and Requirements

**Project name:** DMS_with_Alanine_scan

**Project homepage:**  https://github.com/PapenfussLab/DMS_with_Alanine_scan

**Operating system:** Platform independent

**Programming language:** Python

**Other requirements:** Python 3.10 or higher

**License:** MIT license

RRID: SCR_023949

## Supplementary Material

giad073_GIGA-D-23-00040_Original_Submission

giad073_GIGA-D-23-00040_Revision_1

giad073_GIGA-D-23-00040_Revision_2

giad073_Response_to_Reviewer_Comments_Original_Submission

giad073_Response_to_Reviewer_Comments_Revision_1

giad073_Reviewer_1_Report_Original_Submission Joseph Ng -- 3/21/2023 Reviewed

giad073_Reviewer_1_Report_Revision_1 Joseph Ng -- 7/12/2023 Reviewed

giad073_Reviewer_2_Report_Original_Submission Leopold Parts -- 4/7/2023 Reviewed

giad073_Reviewer_2_Report_Revision_1 Leopold Parts -- 7/21/2023 Reviewed

giad073_Supplemental_Files

## Data Availability

A copy of the data analysis code and a full set of data files required to reproduce this work are openly available in the *GigaScience* repository, GigaDB, under the record described in [138]. MaveDB accession numbers, UniProt accession numbers, and other metadata describing the matched DMS-AS datasets are listed in Supplementary Table S1 (see supporting information).

## Additional Files

**Supplementary Table S1**. All candidate DMS and alanine scanning data with detailed dataset information.

**Supplementary Table S2**. Normalized DMS dataset with protein property features.

**Supplementary Table S3**. Normalized alanine scanning dataset.

**Supplementary Table S4**. DMS/AS correlation on each secondary structural region.

**Supplementary Table S5**. Amount of data with AS scores available.

**Supplementary Fig. S1**. DMS and AS score distribution. The figure shows the kernel estimated density of normalized AS scores and DMS scores for variants with or without available AS data.

**Supplementary Fig. S2**. Decision tree for classifying DMS and AS assay compatibility. The similarity of DMS and AS assays is compared (Methods) and the DMS/AS assay pairs are classified using 3 levels of compatibility (low, medium, high). The leaf-node text and color show the classified assay compatibility. The number indicates the count of assay pairs for each compatibility level.

**Supplementary Fig. S3**. Comparison between published and reimplemented predictors. The plot shows leave-one-protein-out cross-validation performance on predictors built from the published DeMaSk code or our code. The predictors were trained and evaluated on DMS data either provided by the DeMaSk study or curated by our own. The “DeMaSk data & code” result is similar to the published result. For the “Our data & DeMaSk code” result, we used our own data and published code, which shows a median performance around 0.35. This is probably because many more DMS results are included in our data. The similarity of results achieved using “Our data & code” demonstrates the correctness of our reimplementation. (Whiskers show the full value range.)

**Supplementary Fig. S4**. Performance comparison between predictors with or without AS data. The Spearman's *ρ* between DMS scores and predicted scores for each DMS and AS data pair are shown as box plots. Different approaches to filtering the data are shown on the x-axis: “All AS data” used all available data; “Compatibility filtered” used only data of high assay compatibility; “Correlation matched” used only data with the highest regularized correlation for each DMS dataset. The figure does not include data without available AS scores. This means that the different results are not directly comparable since they are computed for different subsets of DMS/AS data pairs (for example, “All AS data” contains all DMS/AS data pairs, but “Compatibility filtered” contains only data pairs of high assay compatibility). Control results are shown as green boxes for predictions on the same residues without AS data as a feature. The underlying *ρ* for each data pair in the control results is the same, but the boxes are shifted due to data filtering. Results for data pairs with only 1 residue are not shown. *P* values were calculated using paired *t*-test and jointly corrected using Holm–Šidák (Methods), **P* < 0.05. Notches show the 95% confidence interval around the median, and whiskers show the full value range.

**Supplementary Fig. S5**. The change in prediction performance for using data of different assay compatibility levels. The change of prediction Spearman's *ρ* for each DMS and AS data pair is shown as box plots. A higher value represents higher prediction accuracy achieved for using AS data. Different data-filtering methods are shown on the x-axis. Results for data pairs with only 1 residue are not shown. *P* values were calculated using Welch's test and jointly corrected using Holm–Šidák (Methods), **P* < 0.05. Notches show the 95% confidence interval around the median, and whiskers show the full value range.

**Supplementary Fig. S6**. Prediction performance is improved while incorporating high-compatibility AS data into the Envision model. The Spearman's *ρ* between experiment DMS scores and predicted scores for each DMS/AS assay pair with high compatibility are shown as box plots. The x-axis shows the predictor used, either Envision or DeMaSk. Control results are shown as green boxes for predictions on the same residues without AS data as a feature. Results for data pairs with only 1 residue are not shown. *P* values were calculated using paired *t*-test and jointly corrected using Holm–Šidák (Methods), **P* < 0.05. Notches show the 95% confidence interval around the median, and whiskers show the full value range.

**Supplementary Fig. S7**. Performance improvement on thresholded correlation matching. The change of prediction *ρ* for each DMS and AS data pair is shown as box plots. Different approaches to filtering/matching the data are shown on the x-axis: “All AS data,” “Compatibility filtered,” and “Correlation matched” are the same results as previously discussed; while doing correlation matching, a further thresholding (0, 0.25, or 0.5) on the regularized DMS/AS correlation values (*ρ_r_*) was applied. Notches show the 95% confidence interval around the median, and whiskers show the full value range.

**Supplementary Fig. S8**. Performance improvement on averaged DMS/AS testing data. This figure shows model performance when we averaged variant scores for DMS or AS data that are (i) published in the same paper, (ii) targeting the same protein region, and (iii) measured by the same type of assays (Supplementary Table S1). The change of prediction *ρ* for each averaged DMS and AS data pair is shown. A higher value represents higher prediction accuracy achieved when using AS data. Different approaches to filtering/matching the data are shown on the x-axis: “All AS data” used all available data; “Compatibility filtered” used only data of high assay compatibility; “Correlation matched” used only data with the highest regularized correlation for each DMS dataset. Results for data pairs with only 1 residue are not shown. Notches show the 95% confidence interval around the median, and whiskers show the full value range.

**Supplementary Fig. S9**. Model performance on various variant effect predictors. The Spearman's *ρ* between DMS scores and predicted scores from different variant effect predictors for each DMS and AS pair are shown as box plots. Results are evaluated on different sets of variant data shown on the x-axis: “All AS data” used all available data; “Compatibility filtered” used only data of high assay compatibility; “Correlation matched” used only AS data with the highest regularized correlation for each DMS dataset. The figure does not include residues without available AS scores. Results for data pairs with only 1 residue are not shown. Notches show the 95% confidence interval around the median, and whiskers show the full value range.

**Supplementary Fig. S10**. Prediction performance change for using all AS data. Each dot represents a DMS/AS data pair. The vertical axis shows the change of prediction *ρ* by using AS data (larger means higher performance achieved by using AS data). The horizontal axis shows the DMS/AS score correlation for *all* variants on the matched residues rather than just alanine substitutions. The colors and shapes of the dots correspond to the target protein, and size indicates the number of variants in each data pair. Results for data pairs with only 1 residue are not shown.

**Supplementary Fig. S11**. Model performance for training with AS data-available residues. The predictors were trained only on variants that have AS data available. Panel A shows the performance visualized by prediction Spearman's *ρ* for DMS scores and predicted scores for each DMS and AS data pair. Different approaches to filtering the data are shown on the x-axis: “All AS data” used all available data; “Compatibility filtered” used only data of high assay compatibility; “Correlation matched” used only AS data with the highest regularized correlation for each DMS dataset. Control results are shown as green boxes for predictions on the same residues without AS data as a feature. Panel B shows change of prediction *ρ* for each DMS and AS data pair. A higher value indicates higher prediction accuracy achieved when using AS data. Different approaches to filtering the data are also shown on the x-axis as described. Notches show the 95% confidence interval around the median, and whiskers show the full value range.

**Supplementary Fig. S12**. Boosting setup shows similar performance as the main result. Each dot represents a filtered DMS/AS data pair of high assay compatibility. The vertical and horizontal axes show the prediction Spearman's *ρ* for either modeled with boosting or the 1-step (main result) setup. The colors and shapes of the dots correspond to the target protein, and size indicates the number of variants in each data pair.

**Supplementary Fig. S13**. Training with DMS scores of alanine substitutions shows similar performance as the main result. The vertical and horizontal axes show the prediction Spearman's *ρ* for predictors either trained with DMS score of alanine substitutions (DMS-Ala) or AS data of high assay compatibility (main result), yet all evaluated on high-compatibility AS data. The colors and shapes of the dots correspond to the target protein, and size indicates the number of variants in each data pair.

**Supplementary Fig. S14**. Count of variant entries for each wild-type or variant amino acid of high assay compatibility data. Neg., negatively; Pos., positively.

**Supplementary Fig. S15**. Relationship between amino acid similarity and model performance. For each amino acid, its similarity to alanine was computed by their DMS score correlation or using BLOSUM scores as shown on the x-axis. The performance improvement (Δρ) for each wild-type (left) or variant (right) amino acid while using AS data was computed as previously mentioned (Fig. 7), with their Spearman's correlation against the similarity measurements shown in the figure. The label for each amino acid is colored by the amino acid physicochemical property. Neg., negatively; Pos., positively.

**Supplementary Information**. Alternative baseline predictor and approaches to incoporate AS data.

## Abbreviations

AS: alanine scanning; DMS: deep mutational scanning.
