# Supplementary material for: Integrating deep mutational scanning and low-throughput mutagenesis data to predict the impact of amino acid variants
Source: Gigascience. 2023 Sep 18;12:giad073. doi: 10.1093/gigascience/giad073 (PMC10506130; doi:10.1093/gigascience/giad073)
Supplement: giad073_GIGA-D-23-00040_Revision_1 [file giad073_giga-d-23-00040_revision_1.pdf]

GigaScience

Integrating deep mutational scanning and low-throughput mutagenesis data to predict the impact of amino acid variants

--Manuscript Draft--

**Manuscript Number:**	GIGA-D-23-00040R1
**Full Title:**	Integrating deep mutational scanning and low-throughput mutagenesis data to predict the impact of amino acid variants
**Article Type:**	Research
**Funding Information:**	National Health and Medical Research Council (116955) — Professor Anthony Troy Papenfuss<br>National Human Genome Research Institute (RM1HG010461) — Dr Alan F. Rubin<br>National Human Genome Research Institute (UM1HG011969) — Dr Alan F. Rubin<br>Lorenzo and Pamela Galli Medical Research Trust — Professor Anthony Troy Papenfuss<br>Stafford Fox Medical Research Foundation — Professor Anthony Troy Papenfuss<br>Melbourne Research Scholarship — Mr Yunfan Fu
**Abstract:**	Background: Evaluating the impact of amino acid variants has been a critical challenge for studying protein function and interpreting genomic data. High-throughput experimental methods like deep mutational scanning (DMS) can measure the effect of large numbers of variants in a target protein, but because DMS studies have not been performed on all proteins, researchers also model DMS data computationally to estimate variant impacts by predictors.<br>Results: In this study, we extended a linear regression-based predictor to explore whether incorporating data from alanine scanning (AS), a widely-used low-throughput mutagenesis method, would improve prediction results. To evaluate our model, we collected 146 AS datasets, mapping to 54 DMS datasets across 22 distinct proteins.<br>Conclusions: We show that improved model performance depends on the compatibility of the DMS and AS assays, and the scale of improvement is closely related to the correlation between DMS and AS results.
**Corresponding Author:**	Alan F. Rubin, PhD<br>Walter and Eliza Hall Institute of Medical Research<br>Parkville, VIC AUSTRALIA
**Corresponding Author Secondary Information:**	
**Corresponding Author's Institution:**	Walter and Eliza Hall Institute of Medical Research
**Corresponding Author's Secondary Institution:**	
**First Author:**	Yunfan Fu
**First Author Secondary Information:**	
**Order of Authors:**	Yunfan Fu<br>Justin Bedő, PhD<br>Anthony Troy Papenfuss, BSc (Hons) PhD<br>Alan F. Rubin, PhD
**Order of Authors Secondary Information:**	
**Response to Reviewers:**	We thank the reviewers for their thoughtful comments, which helped us improve our

	study and the manuscript.<br><br>We have attached a detailed response to the reviews (Review_response_untracked.pdf) as well as a version of the updated manuscript with all changes from the original submission highlighted (diff_manuscript.pdf).
Additional Information:	
Question	Response
Are you submitting this manuscript to a special series or article collection?	No
**Experimental design and statistics**<br><br>Full details of the experimental design and statistical methods used should be given in the Methods section, as detailed in our Minimum Standards Reporting Checklist. Information essential to interpreting the data presented should be made available in the figure legends.<br><br>Have you included all the information requested in your manuscript?	Yes
**Resources**<br><br>A description of all resources used, including antibodies, cell lines, animals and software tools, with enough information to allow them to be uniquely identified, should be included in the Methods section. Authors are strongly encouraged to cite Research Resource Identifiers (RRIDs) for antibodies, model organisms and tools, where possible.<br><br>Have you included the information requested as detailed in our Minimum Standards Reporting Checklist?	Yes
**Availability of data and materials**<br><br>All datasets and code on which the conclusions of the paper rely must be either included in your submission or deposited in publicly available repositories (where available and ethically appropriate), referencing such data using	Yes

a unique identifier in the references and in the “Availability of Data and Materials” section of your manuscript.<br><br>Have you have met the above requirement as detailed in our Minimum Standards Reporting Checklist?	

Powered by Editorial Manager® and ProduXion Manager® from Aries Systems Corporation

Manuscript

Click here to access/download;Manuscript;AS_draft_v6.3_untracked.docx

Integrating deep mutational scanning and low-throughput mutagenesis data to predict the impact of amino acid variants

Authors:

Yunfan Fu[1,2], Justin Bedő[1,2,*], Anthony T. Papenfuss[1,2,3,*,**], Alan F. Rubin[1,2,*,**]

Affiliations:

[1]The Walter and Eliza Hall Institute of Medical Research, Parkville, VIC 3052, Australia.

[2]Department of Medical Biology, The University of Melbourne, Parkville, VIC 3010, Australia.

[3]Peter MacCallum Cancer Centre, Melbourne, VIC 3000, Australia.

* Contributed equally

** To whom correspondence should be addressed (papenfuss@wehi.edu.au & alan.rubin@wehi.edu.au)

Abstract

Background: Evaluating the impact of amino acid variants has been a critical challenge for studying protein function and interpreting genomic data. High-throughput experimental methods like deep mutational scanning (DMS) can measure the effect of large numbers of variants in a target protein, but because DMS studies have not been performed on all proteins, researchers also model DMS data computationally to estimate variant impacts by predictors.

Results: In this study, we extended a linear regression-based predictor to explore whether incorporating data from alanine scanning (AS), a widely used low-throughput mutagenesis

method, would improve prediction results. To evaluate our model, we collected 146 AS datasets, mapping to 54 DMS datasets across 22 distinct proteins.

Conclusions: We show that improved model performance depends on the compatibility of the DMS and AS assays, and the scale of improvement is closely related to the correlation between DMS and AS results.

Keywords: deep mutational scanning, alanine scanning, machine learning, predictor

1 Introduction

Deep mutational scanning (DMS) is a functional genomics method that can experimentally measure the impact of many thousands of protein variants by combining high-throughput sequencing with a functional assay [1]. In a typical DMS, a cDNA library of genetic variants of a target gene is generated, containing all possible single amino acid substitutions. This variant library is then expressed in a functional assay system where the DMS variants can be selected based on their properties. The change in variant frequency in the pre- and post-selection populations is determined by high-throughput sequencing which is then used to calculate a multiplexed functional score that captures the variant's impact [2–4]. The versatility of DMS assays makes it possible to measure variant impact on a wide range of protein properties, including protein binding affinity [5,6], protein abundance [7–9], enzyme activity [10,11] and cell survival [12–14]. So far, hundreds of DMS studies covering tens of thousands of nucleotides have been published [15], and experiments targeting over a hundred additional genes are underway according to MaveRegistry [16].

Computational studies have used DMS data to build predictive models of variant impact. These predictors use supervised or semi-supervised learning models trained on experimental DMS data and various protein features to make predictions [17–23]. Envision is one such method that used protein structural, physicochemical, and evolutionary features to predict variant effect scores and was trained on DMS data from 8 proteins using gradient boosting [17]. Another method, DeMaSk, predicted DMS scores by combining two evolutionary features (protein positional conservation and variant homologous frequency) with a DMS substitution matrix and was trained on data from 17 proteins using a linear model [19]. Deep learning algorithms have also been applied to build protein fitness predictors [18,20], which are usually based only on variant sequences. These variant effect predictors can also be benchmarked using DMS experimental results and assist in the interpretation of experimental data [20,24,25].

Low-throughput mutagenesis experiments that measure tens of variants at a time have also been used extensively to study diverse protein properties, including substrate binding affinity [26,27], protein stability [28,29], and protein-specific activities [30,31]. Alanine scanning (AS) is a widely-used low-throughput mutagenesis method [32,33], and AS data are available for many proteins. In this method, each targeted protein residue is substituted with alanine, and the impacts of these variants are measured by a functional assay [34]. AS experiments are typically used to identify functional hot spots or critical residues in the target protein [35,36] and have been used as a source of independent validation for DMS studies [31,37–39].

In this study, we explore whether a predictive model can be improved by incorporating low-throughput mutagenesis data (Fig 1). We find that AS data can increase prediction accuracy

and that the improvement is related to the similarity of the functional assays and the correlation of DMS and AS results.

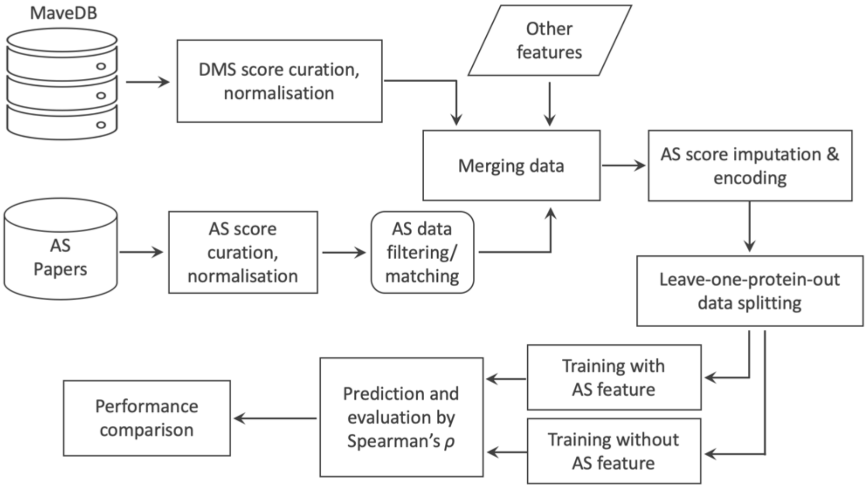

Fig 1. Workflow for model training and testing. DMS and AS datasets are collected from online resources and are normalized. DMS and AS datasets targeting the same protein are then matched, filtered and merged. Two predictors are constructed and tested: the first uses DMS data, AS data and other protein features, and the second uses only DMS data and the same other protein features.

2 Results

2.1 Overview of DMS and alanine scanning (AS) data

To build the predictive model, 130 DMS datasets were collected from MaveDB [40,41] (Supplementary table 1). We searched the literature and found 146 AS datasets targeting the same proteins as 54 of the DMS datasets. In total, we obtained both DMS and AS data for 22 different proteins: 17 human proteins, three yeast proteins, and two bacterial proteins. Most DMS experiments were highly complete, with a mean coverage of 95.0% of all possible single amino acid substitutions assayed in the target region, comprising 373,219 total protein variant measurements. AS data were only available on a small number of protein residues (Fig 2), and we

were able to curate 1,480 alanine substitution scores from the 146 studies. Variant scores from collected DMS and AS studies were linearly normalized to a common scale (see Methods) to make them comparable across datasets (Fig S1).

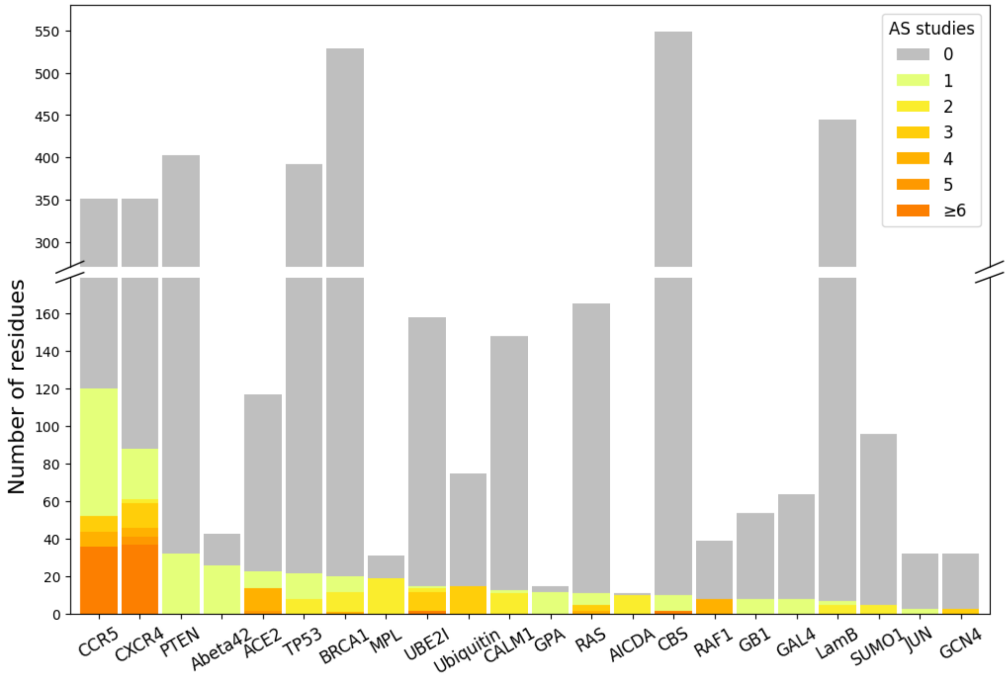

Fig 2. DMS data generally cover more protein residues than AS data. Each bar shows the number of residues assayed by DMS studies on given target proteins. Colour indicates the number of AS studies available for the DMS-tested residues.

2.2 The correlation of DMS and AS scores is related to assay compatibility

To evaluate the similarity of AS and DMS scores, we calculated Spearman's correlation (ρ) between the AS scores and DMS scores for the same alanine substitutions. Since each protein may have results from several AS and DMS experiments, we calculated ρ between each possible pair. The median ρ over DMS and AS data (DMS/AS) pairs was 0.2, indicating that the experimental scores were poorly correlated overall (Fig 3).

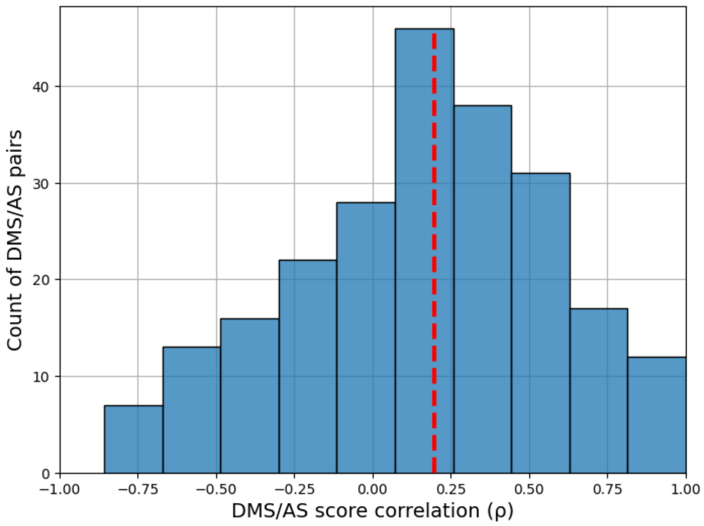

Fig 3. Correlation between DMS and AS data shows substantial variation. We calculated Spearman's ρ between alanine substitution scores in each pair of AS and DMS data. The results for pairs with less than three alanine substitutions are not shown. The red dashed line shows the median ρ.

We then considered if differences between AS and DMS assay designs might contribute to this low agreement between scores. To explore this, we developed a decision tree (Fig S2) to classify whether DMS/AS pairs had low, medium, or high assay compatibility, which we defined as a similarity measurement of the functional assays performed. For example, the DMS assay measuring the binding affinity of a cell surface protein, CXCR4, to its natural ligand [42] has high compatibility with the AS experiment also measuring this ligand binding but has low compatibility with the study on CXCR4's ability to facilitate virus infection [43]. A full assay compatibility table can be found in Supplementary Table 1 with the compatibility classifications and justification for each pair. We then compared DMS and AS score correlation for each compatibility class and found that score correlations were closely related to assay compatibility. Data from low compatibility assays had a median correlation of 0.15, rising to 0.19 for medium compatibility assays and 0.40 for high compatibility assays (Fig 4). This trend of increased correlation for high compatibility assay pairs holds across secondary structures (Table S1).

This link between assay compatibility and score correlation indicates that our decision tree approach was able to capture the similarity between assay systems.

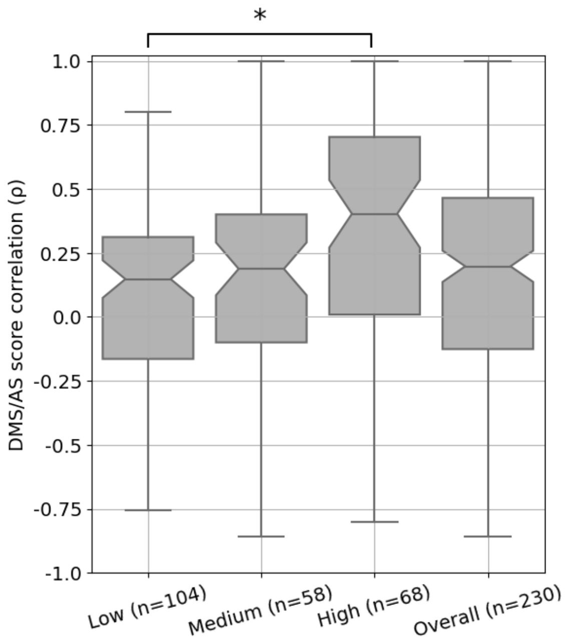

Fig 4. DMS and AS data pairs with high assay compatibility show a higher score correlation. Each box shows the Spearman's ρ between DMS and AS data pairs for each level of assay compatibility or overall. The correlation coefficients were calculated between alanine substitution scores in each pair of AS and DMS datasets. Results for pairs with less than three alanine substitutions were removed. P-values calculated using Welch's test and corrected using Holm-Šidák, *: $p<0.05$; notches show 95% confidence interval around median, and whiskers show the full value range.

2.3 Compatible AS data improve DMS score prediction accuracy

To test if incorporating AS data into DMS score models would improve prediction accuracy, we decided to build a new model based on DeMaSk [19]. We chose DeMaSk because it showed better performance compared to similar methods and was straightforward to modify. The published DeMaSk model predicts DMS scores using protein positional conservation, variant ho-

mologous frequency, and substitution score matrix, and we incorporated AS data as an additional feature. Our new predictor was modelled with all 130 DMS we collected and we applied a leave-one-protein-out cross-validation approach to training and testing, avoiding information leakage for variants of the same protein target [17]. Prediction performance was evaluated using the Spearman's correlation (ρ) between the experimentally-derived DMS scores and the predicted scores for each pair of DMS and AS studies. The performance of our DMS/AS model was compared with a model trained only on DMS data, equivalent to retrained DeMaSk (Fig S3), by calculating the change of prediction ρ (see Methods).

We trained our model with either all or a subset of AS data we collected (Fig 5, Table S2). We first integrated all 146 AS data collected for training and evaluation but observed only a modest improvement of prediction ρ (Fig 5 left box, and Fig S4). We then retrained and evaluated our model on filtered AS data with only high compatibility assays, and observed a median increase in prediction Spearman's ρ of 0.1 compared to the results with no AS data (Fig 5 middle box, and Fig S4). However, training with both high and medium compatibility pairs reduced the performance improvement (Fig S5). These results indicate that medium and low compatibility pairs might provide inconsistent training data, degrading model performance. We also evaluated the impact of including high compatibility AS data in an alternative model based on Envison [17], and found similar results (Fig S6). To differentiate between high assay compatibility and high DMS/AS score correlation, we trained the model using the most highly correlated AS result for each DMS dataset (see Methods). Although the upper quartile was high, the median performance change of this predictor was lower than the high assay compatibility model, suggesting that matching with the highest score correlation alone is insufficient (Fig 5 right box). However, when applying a stricter threshold, the correlation matched models still show

limited improvement (Fig S7). Additionally, to ensure the models performance is not biased by pseudo-replication of multiple datasets, we averaged DMS and AS scores that were part of the same study and type of assay, and saw similar results (Fig S8).

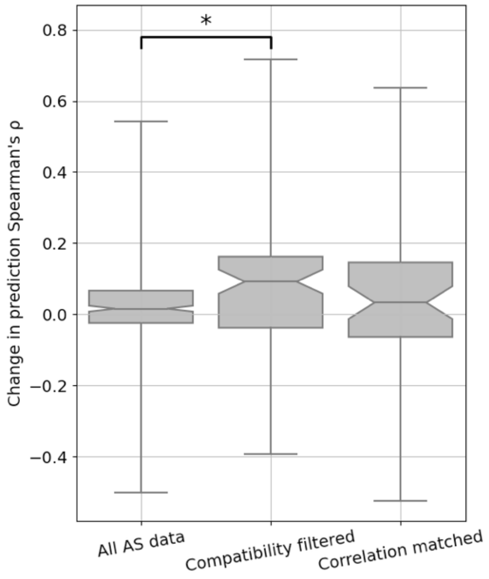

Fig 5. Performance of variant impact prediction is improved using AS data with high assay compatibility. The change in prediction ρ achieved by including the AS data feature for each DMS and AS data pair is shown as box plots. A higher value represents higher prediction accuracy achieved for using AS data. Different approaches to filtering/matching the data are shown on the x-axis: "All AS data" used all available data; "Compatibility filtered" used only data of high assay compatibility; "Correlation matched" used only data with the highest regularised correlation for each DMS dataset. Results for data pairs with only one residue are not shown. P-values were calculated using Welch's test and jointly corrected using Holm-Šidák (Methods), *: $p<0.05$. Notches show the 95% confidence interval around the median, and whiskers show the full value range.

Our compatibility-filtered predictor shows improved prediction accuracy for these regions compared to not only the baseline model, but other widely used predictors as well (Fig S9). To further explore the higher performance of this compatibility-filtered predictor, we examined the relationship between prediction ρ change and score correlation for each high compatibility

DMS/AS pair (Fig 6). For most pairs, prediction performance was improved by using AS data, and the scale of improvement was also related to the score correlation. This relationship could also be observed for multiple DMS/AS pairs from an individual protein, such as CXCR4 and CCR5. We saw the same trend in the predictor trained with all DMS/AS pairs but noted that the performance even of highly correlated pairs was worse, likely due to the influence of low compatibility training data on the model (Fig S10).

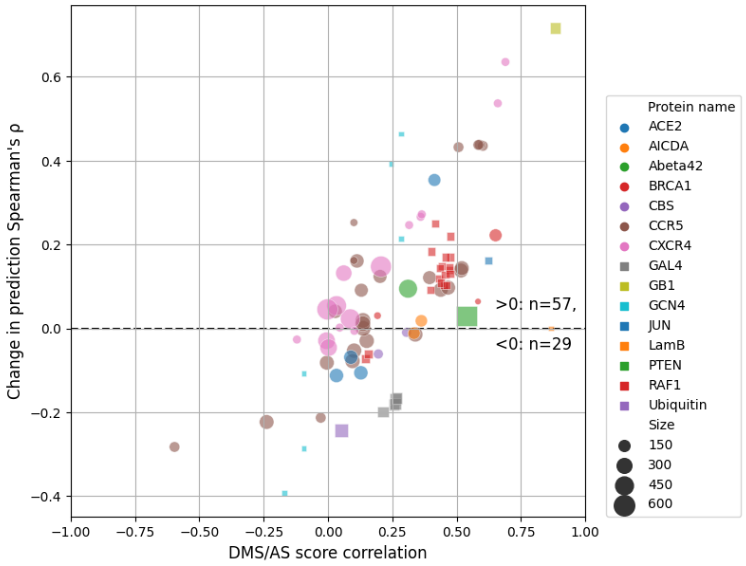

Fig 6. Prediction performance change is related to DMS and AS score correlation. Each dot represents a filtered DMS/AS data pair of high assay compatibility. The vertical axis shows the change of prediction ρ by using AS data (larger means higher performance achieved by using AS data). The horizontal axis shows the DMS/AS score correlation for *all* variants on the matched residues rather than just alanine substitutions. The colours and shapes of the dots correspond to the target protein, and size indicates the number of variants in each data pair. Results for data pairs with only one residue are not shown.

We also explored the consequences of the sparsity of AS data on our model in three ways: i) by training only with variants that have AS data available (Fig S11); ii) by using a boosting

approach that focuses only on residues with AS data (Fig S12) and iii) by using complete alanine substitution information from DMS as the AS feature (Fig S13). The first approach gave lower absolute prediction performance, presumably because the model was under-fitted due to the small number of variants. The last two approaches performed very similarly to the primary model constructed using high-compatibility DMS/AS data and simple mean score imputation.

To test the influence of amino acids on our predictor, we grouped the prediction results by either wild-type or variant amino acid and calculated the prediction improvement when AS data were included (Fig 7). We found that 14 of 19 wild-type amino acids performed better with the addition of AS data, with cysteine showing the largest improvement and performing worst in the model lacking AS data. 18 of 20 variant amino acids benefited from the inclusion of AS data, with marginal performance decrease on lysine and aspartic acid ($|\Delta\rho|<0.01$) (Fig 7). We also noticed that variants to alanine are not most improved, however we observed an overall trend showing higher improvement for amino acids that are physiochemically similar to alanine (Fig S15).

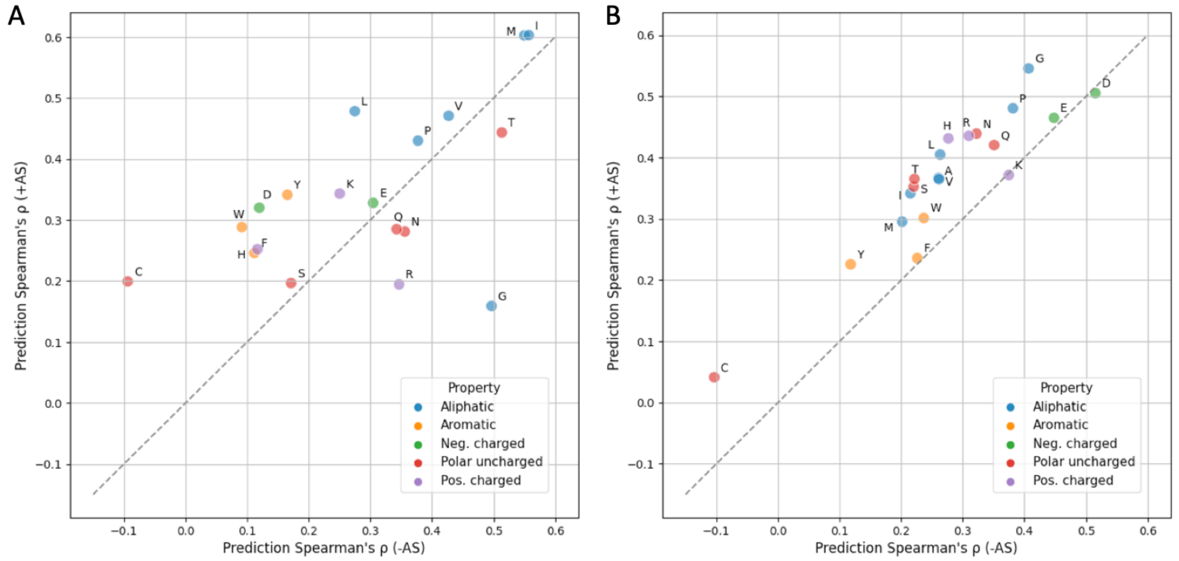

Fig 7. Model performance is generally improved for each wild-type and variant amino acid. Prediction Spearman's ρ when using (y-axis) or not using (x-axis) AS data on each wild-type (**A**) or variant (**B**) amino acid is shown in the scatter plots. The results are coloured according to the property of each amino acid type. Alanine (A) result is not applicable in the first figure since alanine scanning data are always missing when the wildtype is alanine itself. Absolute count for each amino acid can be found in Fig S14. (Neg.: negatively, Pos.: positively)

3 Discussion

In this study, we integrated alanine scanning (AS) data into deep mutational scanning (DMS) score prediction, leading to modest improvements in the accuracy of variant score prediction. We also explored the impact of the diversity of protein properties measured by DMS and AS. Filtering DMS and AS data based on our manual classification of assay type compatibility led to improved prediction performance.

A potential shortcoming of our current approach is that AS data were available for only a small proportion of the DMS data. Although most recent DMS studies can analyze variants of the whole protein, most AS experiments only cover a handful of residues in the target protein, leaving missing AS scores for the vast majority of residues. We explored this here and found that alternative methods for addressing the sparsity of AS data did not improve or degrade performance, but we anticipate further improved prediction accuracy if the low completeness and unevenness of AS data are appropriately handled before modelling.

In this study, we identified the importance of DMS/AS assay compatibility as a crucial factor for improving prediction accuracy. An issue with using this concept is that it further shrinks already sparse data. It also fails to take advantage of the fact that even for low compatible assays some fundamental information like protein abundance can still be mutually captured.

Instead of hard filtering, proper implementation of this underlying information may facilitate variant impact prediction in the future. Nonetheless, filtering on assay compatibility still leads to performance improvement. We also briefly explored whether the consistency of DMS and AS scores can be considered more directly by matching the best correlated AS data for each DMS dataset. Consistency is partially driven by assay compatibility but also reflects other features of the data, such as bias and noise.

The concepts of compatibility and data quality are also relevant to training any DMS-based predictors. DMS assays have been developed to measure variant impacts to distinct protein properties, and a variant can behave similarly to wildtype when measured by one assay yet show altered protein properties in other assay results, which are frequently found in regions with specific biochemical functions [25,52–56]. With more experimental assays to be applied, the diverse measurements may impede the progress of future DMS-based predictors unless this assay effect is properly addressed, for example, by building assay specific predictors. Measurement error is another source of DMS data heterogeneity that potentially affects the model performance. In our current study, DMS scores of protein variants are weighted equally while training. Adjustable weighting can be applied in future studies to adapt the distinct experimental error between individual variants and datasets, reducing the influence of low-confident data.

In summary, we conclude that the careful inclusion of low-throughput mutagenesis data improves the prediction of DMS scores, and the approaches described here can potentially be applied to other prediction methods.

4 Availability of supporting source code and requirements

Project name: DMS_with_Alanine_scan

Project home page: https://github.com/PapenfussLab/DMS_with_Alanine_scan

Operating system: Platform independent

Programming language: Python

Other requirements: Python 3.10 or higher

Licence: MIT Licence

5 List of abbreviations

DMS: deep mutational scanning

AS: alanine scanning

6 Supporting information

Supplementary Table 1: All candidate DMS and alanine scanning data with detailed dataset information.

Supplementary Table 2: Normalized DMS dataset with protein property features.

Supplementary Table 3: Normalized alanine scanning dataset.

7 Author contributions

YF developed the software and wrote the initial draft of the manuscript. AFR conceived the study. JB, AFR, and ATP oversaw the project. All authors reviewed, contributed to, and approved the manuscript.

8 Funding

YF is supported by Melbourne Research Scholarship. ATP was supported by an Australian National Health and Medical Research Council (NHMRC) Senior Research Fellowship (1116955). JB, AFR and ATP were supported by the Lorenzo and Pamela Galli Medical Research Trust. JB and ATP were supported by the Stafford Fox Medical Research Foundation. AFR was supported by the National Human Genome Research Institute of the NIH under award numbers RM1HG010461 and UM1HG011969. The research benefitted from support from the Victorian State Government Operational Infrastructure Support and Australian Government NHMRC Independent Research Institute Infrastructure Support.

9 Methods

9.1 DMS data collection

DMS data were downloaded from MaveDB [40,41] which were then filtered and curated. DMS experiments targeting antibody and virus proteins were removed because of their potentially unique functionality. We retrieved the UniProt accession ID of target proteins by searching the protein names or sequences in UniProt [57], and proteins lacking available UniProt ID were also excluded. Datasets that are computationally processed or their wildtype-like and nonsense-like scores (see Normalization) cannot be identified were also filtered out (Supplementary Table 1). All missense variants with only a single amino acid substitution were curated from the DMS studies for our analysis. A total of 130 DMS experiments from 53 studies [5,6,9–14,24,31,37–39,42,58–94] were collected for our analysis.

9.2 Collection of AS data and other features

The following process was used to search for candidate AS studies. Papers were identified by searching on PubMed and Google Scholar for the "alanine scan" or "alanine scanning" together with the name of candidate proteins. While searching in Google Scholar, we included the protein's UniProt ID rather than molecule name as the search term to reduce false positives. Appropriate AS data were collected from the search results. Western blot results were transformed to values by ImageJ if it was the only experimental data available in the study. A total 146 AS experiments were collected from 45 distinct studies [26–28,30,31,43–46,48,49,84,95–127]. Protein features of Shannon entropy and the logarithm of variant amino acid frequency were downloaded from the DeMaSk online toolkit [19]. The substitution score matrix feature was calculated from the mean of training DMS scores for each of the 380 possible amino acid substitutions before each iteration of cross-validation.

9.3 Normalization

DMS and AS datasets were normalized to a common scale using the following approach adapted from previous studies [17,47]. Let *D* denotes a protein study measuring scores s_i^D for a single variant *i*, s_{wt}^D denotes the scores for wildtype and s_{non}^D represents the score for nonsense-like variants. The normalized scores $s_i'^D$ are given by:

$$s_i'^D := \frac{s_i^D - s_{wt}^D}{s_{wt}^D - s_{non}^D} + 1$$

Wild-type scores were directly identified from the paper or the median score of synonymous variants. For DMS data, since not all DMS studies report score of nonsense variants, we defined the nonsense-like scores as the median DMS scores for the 1% missense variants with the strongest loss of function for each dataset. For AS data, nonsense-like scores were either defined according to the paper or using the extreme values (Supplementary Table 1).

9.4 AS data filtering and matching

AS data subsets were filtered/matched according to either assay compatibility or score correlation. For assay compatibility filtering, we first categorized each DMS or AS assay by the protein property or function using the following assay types: binding affinity, enzyme activity, protein abundance, cell survival, pathogen infection, drug response, ability to perform a novel function, or other protein-specific activities (e.g., transcription activity for transcription factors) (Supplementary Table 1). The DMS/AS assay pairs were then classified into three levels of compatibility based on these categories (Fig S2). For each DMS dataset, we first tried to use only AS data with high assay compatibility for further modelling, removing AS data of medium and low assay compatibility. We then also tried to model with AS data of both high and medium assay compatibility.

For score correlation matching, Spearman's correlation (ρ) is calculated between alanine substitution scores in each pair of AS and DMS data. To avoid influence from the size of AS datasets, we estimated the ρ value with the empirical copula, which is related to the standard estimator by a factor of (n-1)/(n+1) [128,129]:

$$\rho_r := \rho \times \frac{n-1}{n+1}$$

where ρ_r is the regularised correlation coefficient, and n is the number of alanine substitutions used for correlation calculation. For each DMS dataset, AS result with the highest ρ_r was picked for modelling.

9.5 AS data pre-processing

AS data were pre-processed prior to modelling. For variants without available (filtered/matched) AS data, their AS scores were imputed with the mean value of all available AS

scores across all studies. Then the AS data were encoded by the wild-type and variant amino acid type with one-hot-encoding. For each variant, the AS feature is expanded with two one-hot vectors. Each of the vectors has 19 zeros and one non-zero value which was the AS score, with the location of the non-zero value indicating the wild-type or variant amino acid type.

9.6 Training and evaluation of DMS score predictor

To build the predictors, we performed linear regression using the function `sklearn.linear_model.LinearRegression` from scikit-learn [130]. Training and validation data were separated with leave-one-protein-out cross-validation. In this process, data from one protein were withheld for subsequent validation, and the rest were used for training. This process was iterated over all proteins in the data. Variants were inversely weighted during the training process by the number of measurements available, thus compensating for some regions having greater coverage with DMS and AS assays. Predictors were trained on protein features, DMS data and (optionally) AS data using four different filtering or matching strategies: i) all DMS/AS data, ii) compatibility-filtered DMS/AS data, iii) correlation-matched DMS/AS data, and iv) a control, constructed using DMS data only.

In the evaluation process, let V be protein variants assayed by both DMS study D and AS study A. Variant scores are predicted by the previously mentioned predictors either using AS data ($\hat{s}_V^A$) or not ($\hat{s}_V$). Spearman's correlation (ρ) was calculated between the DMS scores s_V^D and each set of predicted scores. The difference of ρ was used to evaluate the performance change ($\Delta\rho_V$).

$$\rho_V^A = \text{Spearman's correlation}(\hat{s}_V^A, s_V^D)$$

$$\rho_V = \text{Spearman's correlation}(\hat{s}_V, s_V^D)$$

$$\Delta\rho_V = \rho_V^A - \rho_V$$

To evaluate, we iterated over variants from each pair of DMS/AS studies. Results were dropped for variants *V* with only one protein residue available during analysis and visualization. Model performance was compared using the following statistical tests. Results in Fig 5 & Fig S5 were tested with Welch's test, and results in Fig S4 & Fig S6 were tested with paired t-tests. The p-values were jointly corrected using the Holm–Šidák method. The 95% confidence interval of median values are calculated by Gaussian-based asymptotic approximation [131].

9.7 Prediction with other variant effect predictors

For PROVEAN [132] and SIFT [133], prediction results on target variants were directly downloaded from the pre-calculated database for PROVEAN. For PolyPhen-2 [134] and GEMME [135], variant scores were computed through their online toolkits, using the default settings. ESM-1v [136] was set up locally and run according to its examples and documentations. EVE [137] results were collected from their pre-calculated database and a benchmarking study [138].

10 References

1. Fowler DM, Fields S. Deep mutational scanning: a new style of protein science. *Nature Methods*. 2014; doi: 10.1038/nmeth.3027.

2. Findlay GM. Linking genome variants to disease: scalable approaches to test the functional impact of human mutations. *Human Molecular Genetics*. 2021; doi: 10.1093/hmg/ddab219.

3. Geck RC, Boyle G, Amorosi CJ, Fowler DM, Dunham MJ. Measuring Pharmacogene Variant Function at Scale Using Multiplexed Assays. *Annual Review of Pharmacology and Toxicology*. 2022; doi: 10.1146/annurev-pharmtox-032221-085807.

4. Weile J, Roth FP. Multiplexed assays of variant effects contribute to a growing genotype–phenotype atlas. *Hum Genet*. 2018; doi: 10.1007/s00439-018-1916-x.

5. Diss G, Lehner B. The genetic landscape of a physical interaction. *eLife*. 2018; doi: 10.7554/eLife.32472.

6. Fowler DM, Araya CL, Fleishman SJ, Kellogg EH, Stephany JJ, Baker D, et al.. High-resolution mapping of protein sequence-function relationships. *Nature Methods*. 2010; doi: 10.1038/nmeth.1492.

7. Amorosi CJ, Chiasson MA, McDonald MG, Wong LH, Sitko KA, Boyle G, et al.. Massively parallel characterization of CYP2C9 variant enzyme activity and abundance. *The American Journal of Human Genetics*. 2021; doi: 10.1016/j.ajhg.2021.07.001.

8. Faure AJ, Domingo J, Schmiedel JM, Hidalgo-Carcedo C, Diss G, Lehner B. Mapping the energetic and allosteric landscapes of protein binding domains. *Nature*. 2022; doi: 10.1038/s41586-022-04586-4.

9. Matreyek KA, Starita LM, Stephany JJ, Martin B, Chiasson MA, Gray VE, et al.. Multiplex assessment of protein variant abundance by massively parallel sequencing. *Nature Genetics*. 2018; doi: 10.1038/s41588-018-0122-z.

10. Mighell TL, Evans-Dutson S, O'Roak BJ. A Saturation Mutagenesis Approach to Understanding PTEN Lipid Phosphatase Activity and Genotype-Phenotype Relationships. *The American Journal of Human Genetics*. 2018; doi: 10.1016/j.ajhg.2018.03.018.

11. Stiffler MA, Hekstra DR, Ranganathan R. Evolvability as a Function of Purifying Selection in TEM-1 β-Lactamase. *Cell*. 2015; doi: 10.1016/j.cell.2015.01.035.

12. Ahler E, Register AC, Chakraborty S, Fang L, Dieter EM, Sitko KA, et al.. A Combined Approach Reveals a Regulatory Mechanism Coupling Src's Kinase Activity, Localization, and Phosphotransferase-Independent Functions. *Molecular Cell*. 2019; doi: 10.1016/j.molcel.2019.02.003.

13. Giacomelli AO, Yang X, Lintner RE, McFarland JM, Duby M, Kim J, et al.. Mutational processes shape the landscape of TP53 mutations in human cancer. *Nature Genetics*. Nature Publishing Group; 2018; doi: 10.1038/s41588-018-0204-y.

14. Roscoe BP, Thayer KM, Zeldovich KB, Fushman D, Bolon DNA. Analyses of the Effects of All Ubiquitin Point Mutants on Yeast Growth Rate. *Journal of Molecular Biology*. 2013; doi: 10.1016/j.jmb.2013.01.032.

15. Tabet D, Parikh V, Mali P, Roth FP, Claussnitzer M. Scalable Functional Assays for the Interpretation of Human Genetic Variation. *Annu Rev Genet*. 2022; doi: 10.1146/annurev-genet-072920-032107.

16. Kuang D, Weile J, Kishore N, Nguyen M, Rubin AF, Fields S, et al.. MaveRegistry: a collaboration platform for multiplexed assays of variant effect. Lu Z, editor. *Bioinformatics*. 2021; doi: 10.1093/bioinformatics/btab215.

17. Gray VE, Hause RJ, Luebeck J, Shendure J, Fowler DM. Quantitative Missense Variant Effect Prediction Using Large-Scale Mutagenesis Data. *Cell Systems*. 2018; doi: 10.1016/j.cels.2017.11.003.

18. Alley EC, Khimulya G, Biswas S, AlQuraishi M, Church GM. Unified rational protein engineering with sequence-based deep representation learning. *Nat Methods*. 2019; doi: 10.1038/s41592-019-0598-1.

19. Munro D, Singh M. DeMaSk: a deep mutational scanning substitution matrix and its use for variant impact prediction. Xu J, editor. *Bioinformatics*. 2020; doi: 10.1093/bioinformatics/btaa1030.

20. Biswas S, Khimulya G, Alley EC, Esvelt KM, Church GM. Low- N protein engineering with data-efficient deep learning. *Nature Methods*. Nature Publishing Group; 2021; doi: 10.1038/s41592-021-01100-y.

21. Høie MH, Cagiada M, Beck Frederiksen AH, Stein A, Lindorff-Larsen K. Predicting and interpreting large-scale mutagenesis data using analyses of protein stability and conservation. *Cell Reports*. 2022; doi: 10.1016/j.celrep.2021.110207.

22. Wu Y, Li R, Sun S, Weile J, Roth FP. Improved pathogenicity prediction for rare human missense variants. *The American Journal of Human Genetics*. 2021; doi: 10.1016/j.ajhg.2021.08.012.

23. Hsu C, Nisonoff H, Fannjiang C, Listgarten J. Learning protein fitness models from evolutionary and assay-labeled data. *Nat Biotechnol*. 2022; doi: 10.1038/s41587-021-01146-5.

24. Findlay GM, Daza RM, Martin B, Zhang MD, Leith AP, Gasperini M, et al.. Accurate classification of BRCA1 variants with saturation genome editing. *Nature*. 2018; doi: 10.1038/s41586-018-0461-z.

25. Cagiada M, Bottaro S, Lindemose S, Schenstrøm SM, Stein A, Hartmann-Petersen R, et al.. Discovering functionally important sites in proteins. bioRxiv;

26. Block C, Janknecht R, Herrmann C, Nassar N, Wittinghofer A. Quantitative structure-activity analysis correlating Ras/Raf interaction in vitro to Raf activation in vivo. *Nature Structural Biology*. Nature Publishing Group; 1996; doi: 10.1038/nsb0396-244.

27. Sloan DJ, Hellinga HW. Dissection of the protein G B1 domain binding site for human IgG Fc fragment. *Protein Science*. 1999; doi: 10.1110/ps.8.8.1643.

28. Fleming KG, Engelman DM. Specificity in transmembrane helix–helix interactions can define a hierarchy of stability for sequence variants. *PNAS*. National Academy of Sciences; 2001; doi: 10.1073/pnas.251367498.

29. Shibata Y, White JF, Serrano-Vega MJ, Magnani F, Aloia AL, Grisshammer R, et al.. Thermostabilization of the Neurotensin Receptor NTS1. *Journal of Molecular Biology*. 2009; doi: 10.1016/j.jmb.2009.04.068.

30. Brzovic PS, Heikaus CC, Kisselev L, Vernon R, Herbig E, Pacheco D, et al.. The Acidic Transcription Activator Gcn4 Binds the Mediator Subunit Gal11/Med15 Using a Simple Protein Interface Forming a Fuzzy Complex. *Molecular Cell*. 2011; doi: 10.1016/j.molcel.2011.11.008.

31. Gajula KS, Huwe PJ, Mo CY, Crawford DJ, Stivers JT, Radhakrishnan R, et al.. High-throughput mutagenesis reveals functional determinants for DNA targeting by activation-induced deaminase. *Nucleic Acids Research*. 2014; doi: 10.1093/nar/gku689.

32. Kortemme T, Kim DE, Baker D. Computational Alanine Scanning of Protein-Protein Interfaces. *Science's STKE*. American Association for the Advancement of Science; 2004; doi: 10.1126/stke.2192004pl2.

33. Morrison KL, Weiss GA. Combinatorial alanine-scanning. *Current Opinion in Chemical Biology*. 2001; doi: 10.1016/S1367-5931(00)00206-4.

34. Cunningham BC, Wells JA. High-resolution epitope mapping of hGH-receptor interactions by alanine-scanning mutagenesis. *Science*. American Association for the Advancement of Science; 1989; doi: 10.1126/science.2471267.

35. DeLano WL. Unraveling hot spots in binding interfaces: progress and challenges. *Current Opinion in Structural Biology*. 2002; doi: 10.1016/S0959-440X(02)00283-X.

36. Eustache S, Leprince J, Tufféry P. Progress with peptide scanning to study structure-activity relationships: the implications for drug discovery. *Expert Opinion on Drug Discovery*. 2016; doi: 10.1080/17460441.2016.1201058.

37. Olson CA, Wu NC, Sun R. A Comprehensive Biophysical Description of Pairwise Epistasis throughout an Entire Protein Domain. *Current Biology*. 2014; doi: 10.1016/j.cub.2014.09.072.

38. Staller MV, Holehouse AS, Swain-Lenz D, Das RK, Pappu RV, Cohen BA. A High-Throughput Mutational Scan of an Intrinsically Disordered Acidic Transcriptional Activation Domain. *Cell Systems*. 2018; doi: 10.1016/j.cels.2018.01.015.

39. Gray VE, Sitko K, Kameni FZN, Williamson M, Stephany JJ, Hasle N, et al.. Elucidating the Molecular Determinants of Aβ Aggregation with Deep Mutational Scanning. *G3 (Bethesda)*. 2019; doi: 10.1534/g3.119.400535.

40. Esposito D, Weile J, Shendure J, Starita LM, Papenfuss AT, Roth FP, et al.. MaveDB: an open-source platform to distribute and interpret data from multiplexed assays of variant effect. *Genome Biol*. 2019; doi: 10.1186/s13059-019-1845-6.

41. Rubin AF, Min JK, Rollins NJ, Da EY, Esposito D, Harrington M, et al.. MaveDB v2: a curated community database with over three million variant effects from multiplexed functional assays. bioRxiv;

42. Heredia JD, Park J, Brubaker RJ, Szymanski SK, Gill KS, Procko E. Mapping Interaction Sites on Human Chemokine Receptors by Deep Mutational Scanning. *The Journal of Immunology*. American Association of Immunologists; 2018; doi: 10.4049/jimmunol.1800343.

43. Tian S, Choi W-T, Liu D, Pesavento J, Wang Y, An J, et al.. Distinct Functional Sites for Human Immunodeficiency Virus Type 1 and Stromal Cell-Derived Factor 1α on CXCR4 Transmembrane Helical Domains. *JVI*. 2005; doi: 10.1128/JVI.79.20.12667-12673.2005.

44. Chabot DJ, Zhang P-F, Quinnan GV, Broder CC. Mutagenesis of CXCR4 Identifies Important Domains for Human Immunodeficiency Virus Type 1 X4 Isolate Envelope-Mediated Membrane Fusion and Virus Entry and Reveals Cryptic Coreceptor Activity for R5 Isolates. *J Virol*. 1999; doi: 10.1128/JVI.73.8.6598-6609.1999.

45. Han DP, Penn-Nicholson A, Cho MW. Identification of critical determinants on ACE2 for SARS-CoV entry and development of a potent entry inhibitor. *Virology*. 2006; doi: 10.1016/j.virol.2006.01.029.

46. Fujita–Yoshigaki J, Shirouzu M, Ito Y, Hattori S, Furuyama S, Nishimura S, et al.. A Constitutive Effector Region on the C-terminal Side of Switch I of the Ras Protein. *J Biol Chem*. American Society for Biochemistry and Molecular Biology; 1995; doi: 10.1074/jbc.270.9.4661.

47. Gray VE, Hause RJ, Fowler DM. Analysis of Large-Scale Mutagenesis Data To Assess the Impact of Single Amino Acid Substitutions. *Genetics*. 2017; doi: 10.1534/genetics.117.300064.

48. Hidalgo P, Ansari AZ, Schmidt P, Hare B, Simkovich N, Farrell S, et al.. Recruitment of the transcriptional machinery through GAL11P: structure and interactions of the GAL4 dimerization domain. *Genes Dev*. 2001; doi: 10.1101/gad.873901.

49. Rodríguez-Escudero I, Oliver MD, Andrés-Pons A, Molina M, Cid VJ, Pulido R. A comprehensive functional analysis of PTEN mutations: implications in tumor- and autism-related syndromes. *Human Molecular Genetics*. 2011; doi: 10.1093/hmg/ddr337.

50. Schröter C, Günther R, Rhiel L, Becker S, Toleikis L, Doerner A, et al.. A generic approach to engineer antibody pH-switches using combinatorial histidine scanning libraries and yeast display. *mAbs*. 2015; doi: 10.4161/19420862.2014.985993.

51. Starace DM, Bezanilla F. Histidine Scanning Mutagenesis of Basic Residues of the S4 Segment of the Shaker K+ Channel. *J Gen Physiol*. 117:469–902001;

52. Cagiada M, Johansson KE, Valanciute A, Nielsen SV, Hartmann-Petersen R, Yang JJ, et al.. Understanding the Origins of Loss of Protein Function by Analyzing the Effects of Thousands of Variants on Activity and Abundance. Ozkan B, editor. *Molecular Biology and Evolution*. 2021; doi: 10.1093/molbev/msab095.

53. Jepsen MM, Fowler DM, Hartmann-Petersen R, Stein A, Lindorff-Larsen K. Chapter 5 - Classifying disease-associated variants using measures of protein activity and stability. In: Pey AL, editor. *Protein Homeostasis Diseases*. Academic Press;

54. Matreyek KA, Stephany JJ, Ahler E, Fowler DM. Integrating thousands of PTEN variant activity and abundance measurements reveals variant subgroups and new dominant negatives in cancers. *Genome Med*. 2021; doi: 10.1186/s13073-021-00984-x.

55. Mighell TL, Thacker S, Fombonne E, Eng C, O'Roak BJ. An Integrated Deep-Mutational-Scanning Approach Provides Clinical Insights on PTEN Genotype-Phenotype Relationships. *The American Journal of Human Genetics*. 2020; doi: 10.1016/j.ajhg.2020.04.014.

56. Nielsen SV, Hartmann-Petersen R, Stein A, Lindorff-Larsen K. Multiplexed assays reveal effects of missense variants in MSH2 and cancer predisposition. *PLOS Genetics*. Public Library of Science; 2021; doi: 10.1371/journal.pgen.1009496.

57. The UniProt Consortium, Bateman A, Martin M-J, Orchard S, Magrane M, Agivetova R, et al.. UniProt: the universal protein knowledgebase in 2021. *Nucleic Acids Research*. 2021; doi: 10.1093/nar/gkaa1100.

58. Andrews B, Fields S. Distinct patterns of mutational sensitivity for λ resistance and maltodextrin transport in Escherichia coli LamB. *Microb Genom*. 2020; doi: 10.1099/mgen.0.000364.

59. Bandaru P, Shah NH, Bhattacharyya M, Barton JP, Kondo Y, Cofsky JC, et al.. Deconstruction of the Ras switching cycle through saturation mutagenesis. *eLife*. 2017; doi: 10.7554/eLife.27810.

60. Bolognesi B, Faure AJ, Seuma M, Schmiedel JM, Tartaglia GG, Lehner B. The mutational landscape of a prion-like domain. *Nat Commun*. 2019; doi: 10.1038/s41467-019-12101-z.

61. Bridgford JL, Lee SM, Lee CMM, Guglielmelli P, Rumi E, Pietra D, et al.. Novel drivers and modifiers of MPL-dependent oncogenic transformation identified by deep mutational scanning. *Blood*. American Society of Hematology; 2020; doi: 10.1182/blood.2019002561.

62. Chan KK, Dorosky D, Sharma P, Abbasi SA, Dye JM, Kranz DM, et al.. Engineering human ACE2 to optimize binding to the spike protein of SARS coronavirus 2. *Science*. American Association for the Advancement of Science; 2020; doi: 10.1126/science.abc0870.

63. Chiasson MA, Rollins NJ, Stephany JJ, Sitko KA, Matreyek KA, Verby M, et al.. Multiplexed measurement of variant abundance and activity reveals VKOR topology, active site and human variant impact. *Elife*. 2020; doi: 10.7554/eLife.58026.

64. Elazar A, Weinstein J, Biran I, Fridman Y, Bibi E, Fleishman SJ. Mutational scanning reveals the determinants of protein insertion and association energetics in the plasma membrane. Shan Y, editor. *eLife*. eLife Sciences Publications, Ltd; 2016; doi: 10.7554/eLife.12125.

65. Firnberg E, Labonte JW, Gray JJ, Ostermeier M. A Comprehensive, High-Resolution Map of a Gene's Fitness Landscape. *Mol Biol Evol*. 2014; doi: 10.1093/molbev/msu081.

66. Hietpas RT, Jensen JD, Bolon DNA. Experimental illumination of a fitness landscape. *Proceedings of the National Academy of Sciences*. 2011; doi: 10.1073/pnas.1016024108.

67. Hietpas RT, Bank C, Jensen JD, Bolon DNA. Shifting fitness landscapes in response to altered environments. *Evolution*. 2013; doi: 10.1111/evo.12207.

68. Jiang L, Mishra P, Hietpas RT, Zeldovich KB, Bolon DNA. Latent Effects of Hsp90 Mutants Revealed at Reduced Expression Levels. *PLOS Genetics*. Public Library of Science; 2013; doi: 10.1371/journal.pgen.1003600.

69. Jiang RJ. Exhaustive Mapping of Missense Variation in Coronary Heart Disease-related Genes [Thesis]. University of Toronto;

70. Keskin A, Akdoğan E, Dunn CD. Evidence for Amino Acid Snorkeling from a High-Resolution, *In Vivo* Analysis of Fis1 Tail-Anchor Insertion at the Mitochondrial Outer Membrane. *Genetics*. 2017; doi: 10.1534/genetics.116.196428.

71. Kitzman JO, Starita LM, Lo RS, Fields S, Shendure J. Massively parallel single-amino-acid mutagenesis. *Nat Methods*. 2015; doi: 10.1038/nmeth.3223.

72. Kotler E, Shani O, Goldfeld G, Lotan-Pompan M, Tarcic O, Gershoni A, et al.. A Systematic p53 Mutation Library Links Differential Functional Impact to Cancer Mutation Pattern and Evolutionary Conservation. *Molecular Cell*. Elsevier; 2018; doi: 10.1016/j.molcel.2018.06.012.

73. Kowalsky CA, Whitehead TA. Determination of binding affinity upon mutation for type I dockerin–cohesin complexes from Clostridium thermocellum and Clostridium cellulolyticum using deep sequencing. *Proteins: Structure, Function, and Bioinformatics*. 2016; doi: 10.1002/prot.25175.

74. McLaughlin Jr RN, Poelwijk FJ, Raman A, Gosal WS, Ranganathan R. The spatial architecture of protein function and adaptation. *Nature*. 2012; doi: 10.1038/nature11500.

75. Melamed D, Young DL, Gamble CE, Miller CR, Fields S. Deep mutational scanning of an RRM domain of the Saccharomyces cerevisiae poly(A)-binding protein. *RNA*. 2013; doi: 10.1261/rna.040709.113.

76. Mishra P, Flynn JM, Starr TN, Bolon DNA. Systematic Mutant Analyses Elucidate General and Client-Specific Aspects of Hsp90 Function. *Cell Reports*. 2016; doi: 10.1016/j.celrep.2016.03.046.

77. Nedrud D, Coyote-Maestas W, Schmidt D. A large-scale survey of pairwise epistasis reveals a mechanism for evolutionary expansion and specialization of PDZ domains. *Proteins: Structure, Function, and Bioinformatics*. 2021; doi: 10.1002/prot.26067.

78. Newberry RW, Arhar T, Costello J, Hartoularos GC, Maxwell AM, Naing ZZC, et al.. Robust Sequence Determinants of α-Synuclein Toxicity in Yeast Implicate Membrane Binding. *ACS Chem Biol*. 2020; doi: 10.1021/acschembio.0c00339.

79. Newberry RW, Leong JT, Chow ED, Kampmann M, DeGrado WF. Deep mutational scanning reveals the structural basis for α-synuclein activity. *Nat Chem Biol*. 2020; doi: 10.1038/s41589-020-0480-6.

80. Roscoe BP, Bolon DNA. Systematic Exploration of Ubiquitin Sequence, E1 Activation Efficiency, and Experimental Fitness in Yeast. *Journal of Molecular Biology*. 2014; doi: 10.1016/j.jmb.2014.05.019.

81. Sarkisyan KS, Bolotin DA, Meer MV, Usmanova DR, Mishin AS, Sharonov GV, et al.. Local fitness landscape of the green fluorescent protein. *Nature*. Nature Publishing Group; 2016; doi: 10.1038/nature17995.

82. Silverstein RA, Sun S, Verby M, Weile J, Wu Y, Roth FP. A systematic genotype-phenotype map for missense variants in the human intellectual disability-associated gene GDI1. bioRxiv;

83. Starita LM, Pruneda JN, Lo RS, Fowler DM, Kim HJ, Hiatt JB, et al.. Activity-enhancing mutations in an E3 ubiquitin ligase identified by high-throughput mutagenesis. *PNAS*. 2013; doi: 10.1073/pnas.1303309110.

84. Starita LM, Young DL, Islam M, Kitzman JO, Gullingsrud J, Hause RJ, et al.. Massively Parallel Functional Analysis of BRCA1 RING Domain Variants. *Genetics*. 2015; doi: 10.1534/genetics.115.175802.

85. Starita LM, Islam MM, Banerjee T, Adamovich AI, Gullingsrud J, Fields S, et al.. A Multiplex Homology-Directed DNA Repair Assay Reveals the Impact of More Than 1,000 BRCA1 Missense Substitution Variants on Protein Function. *The American Journal of Human Genetics*. 2018; doi: 10.1016/j.ajhg.2018.07.016.

86. Suiter CC, Moriyama T, Matreyek KA, Yang W, Scaletti ER, Nishii R, et al.. Massively parallel variant characterization identifies *NUDT15* alleles associated with thiopurine toxicity. *Proc Natl Acad Sci USA*. 2020; doi: 10.1073/pnas.1915680117.

87. Sun S, Weile J, Verby M, Wu Y, Wang Y, Cote AG, et al.. A proactive genotype-to-patient-phenotype map for cystathionine beta-synthase. *Genome Med*. 2020; doi: 10.1186/s13073-020-0711-1.

88. Thompson S, Zhang Y, Ingle C, Reynolds KA, Kortemme T. Altered expression of a quality control protease in E. coli reshapes the in vivo mutational landscape of a model enzyme. *eLife*. 2020; doi: 10.7554/eLife.53476.

89. Trenker R, Wu X, Nguyen JV, Wilcox S, Rubin AF, Call ME, et al.. Human and viral membrane–associated E3 ubiquitin ligases MARCH1 and MIR2 recognize different features of CD86 to downregulate surface expression. *Journal of Biological Chemistry*. Elsevier; 2021; doi: 10.1016/j.jbc.2021.100900.

90. Weile J, Sun S, Cote AG, Knapp J, Verby M, Mellor JC, et al.. A framework for exhaustively mapping functional missense variants. *Mol Syst Biol*. 2017; doi: 10.15252/msb.20177908.

91. Weile J, Kishore N, Sun S, Maaieh R, Verby M, Li R, et al.. Shifting landscapes of human MTHFR missense-variant effects. *The American Journal of Human Genetics*. Elsevier; 2021; doi: 10.1016/j.ajhg.2021.05.009.

92. Wrenbeck EE, Bedewitz MA, Klesmith JR, Noshin S, Barry CS, Whitehead TA. An Automated Data-Driven Pipeline for Improving Heterologous Enzyme Expression. *ACS Synth Biol*. American Chemical Society; 2019; doi: 10.1021/acssynbio.8b00486.

93. Zhang L, Sarangi V, Moon I, Yu J, Liu D, Devarajan S, et al.. CYP2C9 and CYP2C19: Deep Mutational Scanning and Functional Characterization of Genomic Missense Variants. *Clinical and Translational Science*. 2020; doi: https://doi.org/10.1111/cts.12758.

94. Zinkus-Boltz J, DeValk C, Dickinson BC. A Phage-Assisted Continuous Selection Approach for Deep Mutational Scanning of Protein–Protein Interactions. *ACS Chem Biol*. American Chemical Society; 2019; doi: 10.1021/acschembio.9b00669.

95. Bernier-Villamor V, Sampson DA, Matunis MJ, Lima CD. Structural Basis for E2-Mediated SUMO Conjugation Revealed by a Complex between Ubiquitin-Conjugating Enzyme Ubc9 and RanGAP. *Cell*. 108:122002;

96. Blanpain C, Doranz BJ, Vakili J, Rucker J, Govaerts C, Baik SSW, et al.. Multiple Charged and Aromatic Residues in CCR5 Amino-terminal Domain Are Involved in High Affinity Binding of Both Chemokines and HIV-1 Env Protein. *J Biol Chem*. 1999; doi: 10.1074/jbc.274.49.34719.

97. Brzovic PS, Keeffe JR, Nishikawa H, Miyamoto K, Fox D, Fukuda M, et al.. Binding and recognition in the assembly of an active BRCA1/BARD1 ubiquitin-ligase complex. *Proceedings of the National Academy of Sciences*. 2003; doi: 10.1073/pnas.0836054100.

98. Chen S, Wu J, Zhong S, Li Y, Zhang P, Ma J, et al.. iASPP mediates p53 selectivity through a modular mechanism fine-tuning DNA recognition. *Proc Natl Acad Sci USA*. 2019; doi: 10.1073/pnas.1909393116.

99. Chupreta S, Holmstrom S, Subramanian L, Iñiguez-Lluhí JA. A Small Conserved Surface in SUMO Is the Critical Structural Determinant of Its Transcriptional Inhibitory Properties. *MCB*. 2005; doi: 10.1128/MCB.25.10.4272-4282.2005.

100. Cobb JA, Roberts DM. Structural Requirements for N-Trimethylation of Lysine 115 of Calmodulin. *Journal of Biological Chemistry*. 2000; doi: 10.1074/jbc.M002332200.

101. Coyne RS, McDonald HB, Edgemon K, Brody LC. Functional Characterization of BRCA1 Sequence Variants using a Yeast Small Colony Phenotype Assay. *Cancer Biology & Therapy*. 2004; doi: 10.4161/cbt.3.5.809.

102. Denker K, Orlik F, Schiffler B, Benz R. Site-directed Mutagenesis of the Greasy Slide Aromatic Residues Within the LamB (Maltoporin) Channel of Escherichia coli: Effect on Ion and Maltopentaose Transport. *Journal of Molecular Biology*. 2005; doi: 10.1016/j.jmb.2005.07.025.

103. Dragic T, Trkola A, Lin SW, Nagashima KA, Kajumo F, Zhao L, et al.. Amino-Terminal Substitutions in the CCR5 Coreceptor Impair gp120 Binding and Human Immunodeficiency Virus Type 1 Entry. *J Virol*. 1998; doi: 10.1128/JVI.72.1.279-285.1998.

104. Dragic T, Trkola A, Thompson DAD, Cormier EG, Kajumo FA, Maxwell E, et al.. A binding pocket for a small molecule inhibitor of HIV-1 entry within the transmembrane helices of CCR5. *Proceedings of the National Academy of Sciences*. 2000; doi: 10.1073/pnas.090576697.

105. Ecsédi P, Gógl G, Hóf H, Kiss B, Harmat V, Nyitray L. Structure Determination of the Transactivation Domain of p53 in Complex with S100A4 Using Annexin A2 as a Crystallization Chaperone. *Structure*. 2020; doi: 10.1016/j.str.2020.05.001.

106. Kopecká J, Krijt J, Raková K, Kožich V. Restoring assembly and activity of cystathionine β-synthase mutants by ligands and chemical chaperones. *Journal of Inherited Metabolic Disease*. 2011; doi: 10.1007/s10545-010-9087-5.

107. Kožich V, Sokolová J, Klatovská V, Krijt J, Janošík M, Jelínek K, et al.. Cystathionine β-synthase mutations: effect of mutation topology on folding and activity. *Hum Mutat*. 2010; doi: 10.1002/humu.21273.

108. Kruger W d., Wang L, Jhee K h., Singh R h., Elsas II L j.. Cystathionine β-synthase deficiency in Georgia (USA): Correlation of clinical and biochemical phenotype with genotype. *Human Mutation*. 2003; doi: 10.1002/humu.10290.

109. Lee SY, Pullen L, Virgil DJ, Castañeda CA, Abeykoon D, Bolon DNA, et al.. Alanine Scan of Core Positions in Ubiquitin Reveals Links between Dynamics, Stability, and Function. *Journal of Molecular Biology*. 2014; doi: 10.1016/j.jmb.2013.10.042.

110. Li W, Zhang C, Sui J, Kuhn JH, Moore MJ, Luo S, et al.. Receptor and viral determinants of SARS-coronavirus adaptation to human ACE2. *EMBO J*. 2005; doi: 10.1038/sj.emboj.7600640.

111. Lin G, Baribaud F, Romano J, Doms RW, Hoxie JA. Identification of gp120 Binding Sites on CXCR4 by Using CD4-Independent Human Immunodeficiency Virus Type 2 Env Proteins. *JVI*. 2003; doi: 10.1128/JVI.77.2.931-942.2003.

112. Mascle XH, Lussier-Price M, Cappadocia L, Estephan P, Raiola L, Omichinski JG, et al.. Identification of a Non-covalent Ternary Complex Formed by PIAS1, SUMO1, and UBC9 Proteins Involved in Transcriptional Regulation. *Journal of Biological Chemistry*. 2013; doi: 10.1074/jbc.M113.486845.

113. Matthews EE, Thévenin D, Rogers JM, Gotow L, Lira PD, Reiter LA, et al.. Thrombopoietin receptor activation: transmembrane helix dimerization, rotation, and allosteric modulation. *The FASEB Journal*. 2011; doi: https://doi.org/10.1096/fj.10-178673.

114. Mayfield JA, Davies MW, Dimster-Denk D, Pleskac N, McCarthy S, Boydston EA, et al.. Surrogate Genetics and Metabolic Profiling for Characterization of Human Disease Alleles. *Genetics*. 2012; doi: 10.1534/genetics.111.137471.

115. Navenot J-M, Wang Z, Trent JO, Murray JL, Hu Q, DeLeeuw L, et al.. Molecular anatomy of CCR5 engagement by physiologic and viral chemokines and HIV-1 envelope glycoproteins: differences in primary structural requirements for RANTES, MIP-1α, and vMIP-II binding11Edited by P. E. Wright. *Journal of Molecular Biology*. 2001; doi: 10.1006/jmbi.2001.5086.

116. Peng L, Damschroder MM, Cook KE, Wu H, Dall'Acqua WF. Molecular basis for the antagonistic activity of an anti-CXCR4 antibody. *mAbs*. 2016; doi: 10.1080/19420862.2015.1113359.

117. Peterson BR, Sun LJ, Verdine GL. A critical arginine residue mediates cooperativity in the contact interface between transcription factors NFAT and AP-1. *Proceedings of the National Academy of Sciences*. 1996; doi: 10.1073/pnas.93.24.13671.

118. Rabut GEE, Konner JA, Kajumo F, Moore JP, Dragic T. Alanine Substitutions of Polar and Nonpolar Residues in the Amino-Terminal Domain of CCR5 Differently Impair Entry of Macrophage- and Dualtropic Isolates of Human Immunodeficiency Virus Type 1. *J Virol*. 1998; doi: 10.1128/JVI.72.4.3464-3468.1998.

119. Ransburgh DJR, Chiba N, Ishioka C, Toland AE, Parvin JD. Identification of Breast Tumor Mutations in *BRCA1* That Abolish Its Function in Homologous DNA Recombination. *Cancer Res*. 2010; doi: 10.1158/0008-5472.CAN-09-2850.

120. Tan Y, Tong P, Wang J, Zhao L, Li J, Yu Y, et al.. The Membrane-Proximal Region of C–C Chemokine Receptor Type 5 Participates in the Infection of HIV-1. *Front Immunol*. 2017; doi: 10.3389/fimmu.2017.00478.

121. Towler WI, Zhang J, Ransburgh DJR, Toland AE, Ishioka C, Chiba N, et al.. Analysis of BRCA1 Variants in Double-Strand Break Repair by Homologous Recombination and Single-Strand Annealing. *Human Mutation*. 2013; doi: 10.1002/humu.22251.

122. Trent JO, Wang Z, Murray JL, Shao W, Tamamura H, Fujii N, et al.. Lipid Bilayer Simulations of CXCR4 with Inverse Agonists and Weak Partial Agonists. *J Biol Chem*. 2003; doi: 10.1074/jbc.M307850200.

123. Van Gelder P, Dumas F, Bartoldus I, Saint N, Prilipov A, Winterhalter M, et al.. Sugar Transport through Maltoporin of *Escherichia coli* : Role of the Greasy Slide. *J Bacteriol*. 2002; doi: 10.1128/JB.184.11.2994-2999.2002.

124. VanBerkum MF, Means AR. Three amino acid substitutions in domain I of calmodulin prevent the activation of chicken smooth muscle myosin light chain kinase. *J Biol Chem*. American Society for Biochemistry and Molecular Biology; 266:21488–951991;

125. Wei Q, Wang L, Wang Q, Kruger WD, Dunbrack RL. Testing computational prediction of missense mutation phenotypes: Functional characterization of 204 mutations of human cystathionine beta synthase. *Proteins: Structure, Function, and Bioinformatics*. 2010; doi: 10.1002/prot.22722.

126. Williams AD, Shivaprasad S, Wetzel R. Alanine Scanning Mutagenesis of Aβ(1-40) Amyloid Fibril Stability. *Journal of Molecular Biology*. 2006; doi: 10.1016/j.jmb.2006.01.041.

127. Zhang J, Rao E, Dioszegi M, Kondru R, DeRosier A, Chan E, et al.. The Second Extracellular Loop of CCR5 Contains the Dominant Epitopes for Highly Potent Anti-Human Immunodeficiency Virus Monoclonal Antibodies. *AAC*. 2007; doi: 10.1128/AAC.01302-06.

128. Nelsen RB. An introduction to copulas. 2nd ed. New York: Springer;

129. Bedő J, Ong CS. Multivariate Spearman's rho for aggregating ranks using copulas. *Journal of Machine Learning Research*. arXiv; 2016; doi: 10.48550/ARXIV.1410.4391.

130. Pedregosa F, Varoquaux G, Gramfort A, Michel V, Thirion B, Grisel O, et al.. Scikit-learn: Machine Learning in Python. *Journal of machine Learning research*. :2825–30 2011;

131. Hunter JD. Matplotlib: A 2D Graphics Environment. *Computing in Science & Engineering*. 2007; doi: 10.1109/MCSE.2007.55.

132. Choi Y, Sims GE, Murphy S, Miller JR, Chan AP. Predicting the Functional Effect of Amino Acid Substitutions and Indels. de Brevern AG, editor. *PLoS ONE*. 2012; doi: 10.1371/journal.pone.0046688.

133. Vaser et al.. SIFT missense predictions for genomes. *Nature Protocols*. 2016;

134. Adzhubei IA, Schmidt S, Peshkin L, Ramensky VE, Gerasimova A, Bork P, et al.. A method and server for predicting damaging missense mutations. *Nature Methods*. 2010; doi: 10.1038/nmeth0410-248.

135. Laine E, Karami Y, Carbone A. GEMME: A Simple and Fast Global Epistatic Model Predicting Mutational Effects. *Molecular Biology and Evolution*. 2019; doi: 10.1093/molbev/msz179.

136. Meier J, Rao R, Verkuil R, Liu J, Sercu T, Rives A. Language models enable zero-shot prediction of the effects of mutations on protein function. bioRxiv;

137. Frazer J, Notin P, Dias M, Gomez A, Min JK, Brock K, et al.. Disease variant prediction with deep generative models of evolutionary data. *Nature*. 2021; doi: 10.1038/s41586-021-04043-8.

138. Livesey BJ, Marsh JA. Updated benchmarking of variant effect predictors using deep mutational scanning. bioRxiv;

139. González J, Dai Z, Hennig P, Lawrence ND. Batch Bayesian Optimization via Local Penalization. arXiv;

Supplementary material

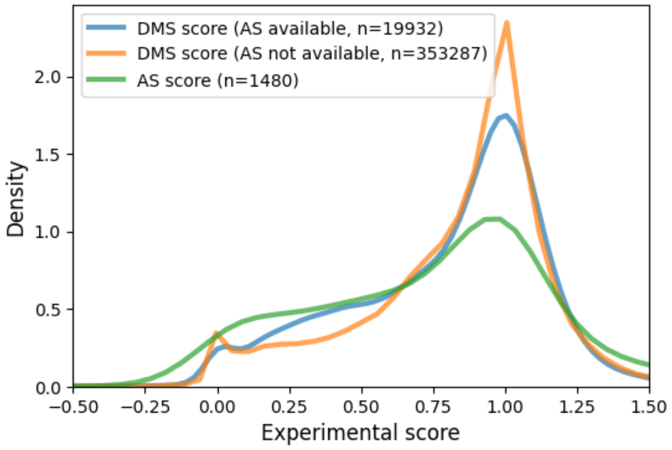

Fig S1. DMS and AS score distribution. The figure shows the kernel estimated density of normalized AS scores and DMS scores for variants with or without available AS data.

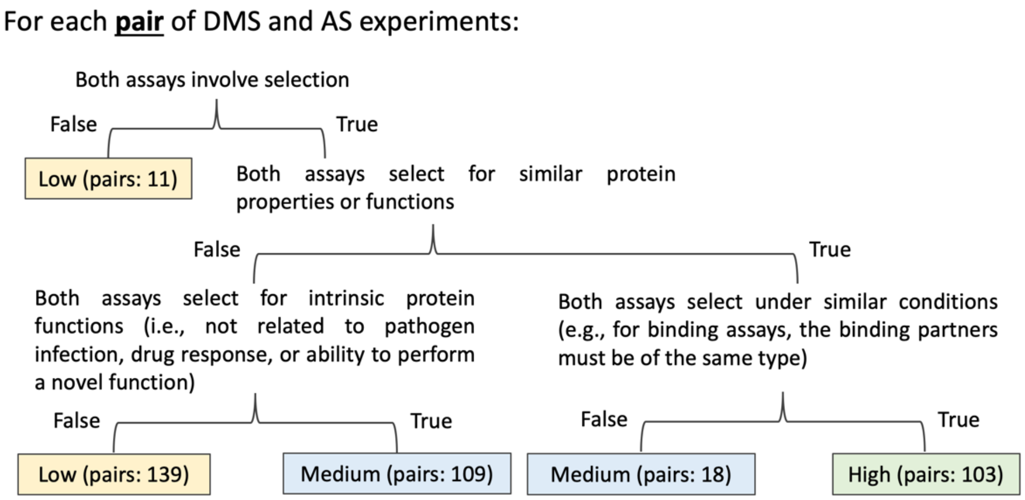

Fig S2. Decision tree for classifying DMS and AS assay compatibility. The similarity of DMS and AS assays are compared (Methods) and the DMS/AS assay pairs are classified using three levels of compatibility (low, medium, high). The leaf-node text and color show the classified assay compatibility. The number indicates the count of assay pairs for each compatibility level.

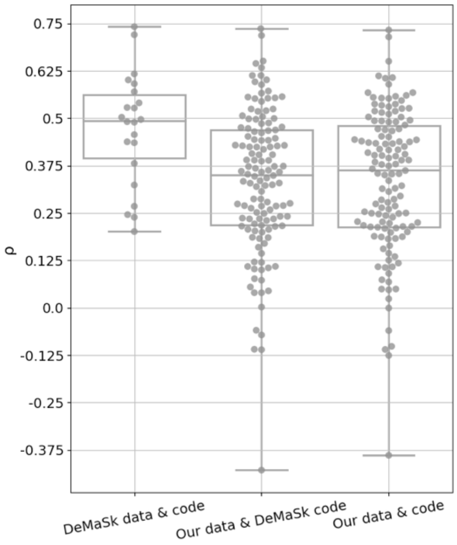

Fig S3. Comparison between published and re-implemented predictors. The plot shows leave-one-protein-out cross-validation performance on predictors built from the published DeMaSk code or our code. The predictors were trained and evaluated on DMS data either provided by the DeMaSk study or curated by our own. The "DeMaSk data & code" result is similar to the published result. For the "Our data & DeMaSk code" result, we used our own data and published code which shows a median performance around 0.35. This is probably because many more DMS results are included in our data. The similarity of results achieved using "Our data & code" demonstrates the correctness of our re-implementation. (Whiskers show the full value range)

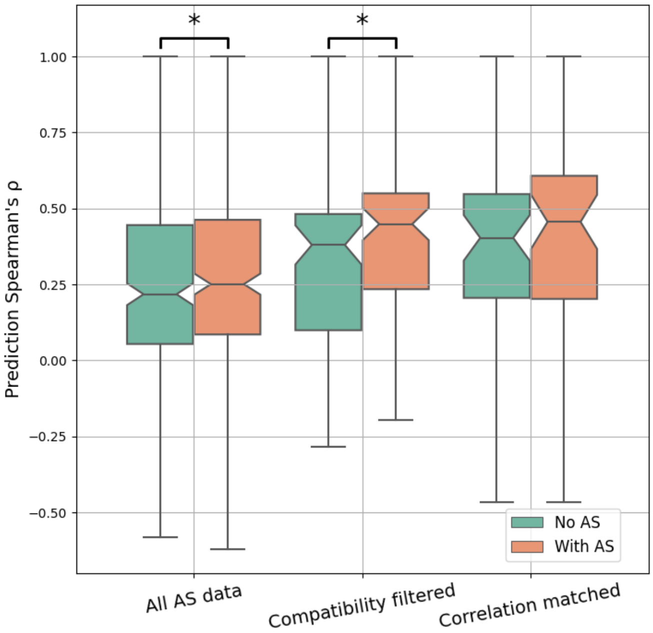

Fig S4. Performance comparison between predictors with or without AS data. The Spearman's ρ between DMS scores and predicted scores for each DMS and AS data pair are shown as box plots. Different approaches to filtering the data are shown on the x-axis: "All AS data" used all available data; "Compatibility filtered" used only data of high assay compatibility; "Correlation matched" used only data with the highest regularised correlation for each DMS dataset. The figure does not include data without available AS scores. This means that the different results are not directly comparable since they are computed for different subsets of DMS/AS data pairs (for example, "All AS data" contains all DMS/AS data pairs, but "Compatibility filtered" contains only data pairs of high assay compatibility). Control results are shown as green boxes for predictions on the same residues without AS data as a feature. The underlying ρ for each data pair in the control results is the same, but the boxes are shifted due to data filtering. Results for data pairs with only one residue are not shown. P-values were calculated using paired t-test and jointly corrected using Holm-Šidák (Methods), *: $p<0.05$. Notches show the 95% confidence interval around the median, and whiskers show the full value range.

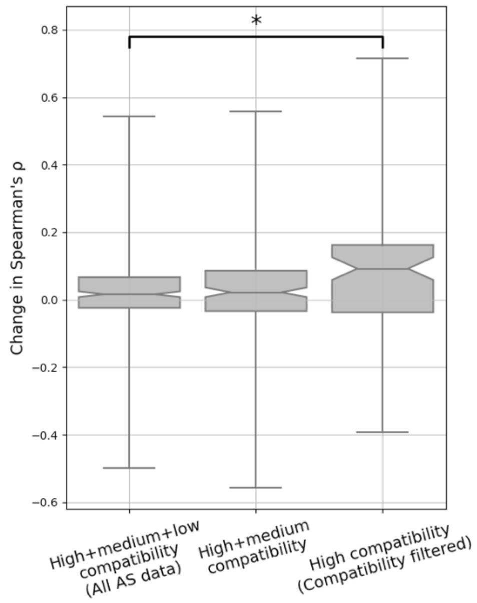

Fig S5. The change in prediction performance for using data of different assay compatibility levels. The change of prediction Spearman's ρ for each DMS and AS data pair is shown as box plots. A higher value represents higher prediction accuracy achieved for using AS data. Different data filtering methods are shown on the x-axis. Results for data pairs with only one residue are not shown. P-values were calculated using Welch's test and jointly

corrected using Holm-Šidák (Methods), *: p<0.05. Notches show the 95% confidence interval around the median, and whiskers show the full value range.

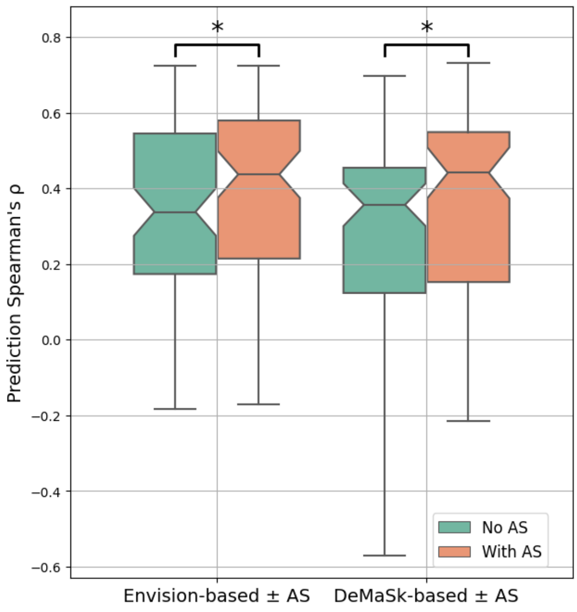

Fig S6. Prediction performance is improved while incorporating high compatibility AS data into the Envision model. The Spearman's ρ between experiment DMS scores and predicted scores for each DMS/AS assay pair with high compatibility are shown as box plots. The x-axis shows the predictor used, either Envision or DeMaSk. Control results are shown as green boxes for predictions on the same residues without AS data as a feature. Results for data pairs with only one residue are not shown. P-values were calculated using paired t-test and jointly corrected using Holm-Šidák (Methods), *: p<0.05. Notches show the 95% confidence interval around the median, and whiskers show the full value range.

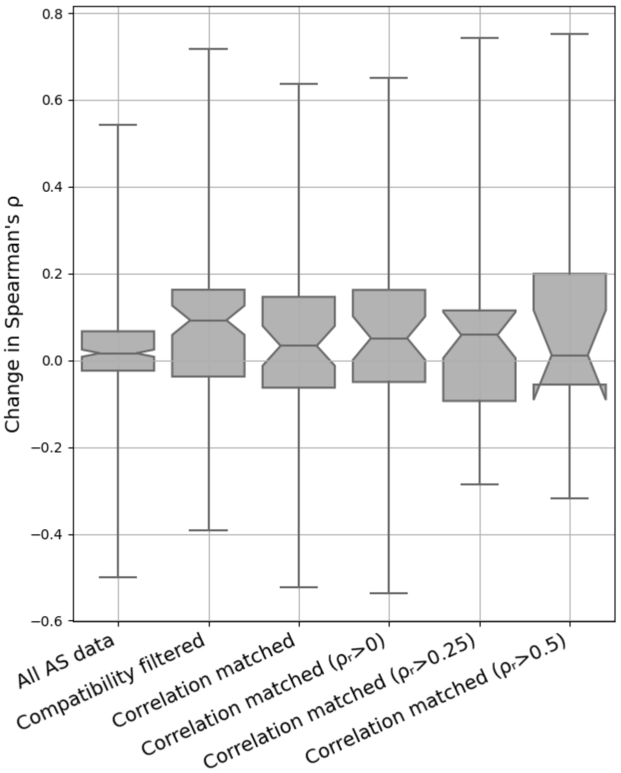

Fig S7. Performance improvement on thresholded correlation matching. The change of prediction ρ for each DMS and AS data pair is shown as box plots. Different approaches to filtering/matching the data are shown on the x-axis: "All AS data", "Compatibility filtered" and "Correlation matched" are the same results as previously discussed; while doing correlation matching, a further thresholding (0, 0.25 or 0.5) on the regularized DMS/AS correlation values (ρ_r) was applied. Notches show the 95% confidence interval around the median, and whiskers show the full value range.

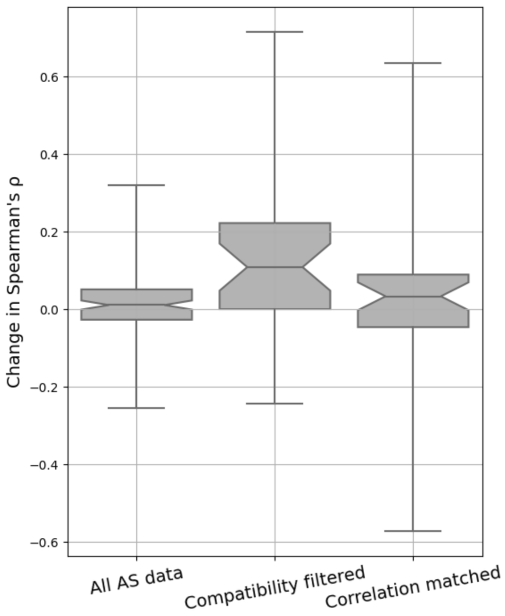

Fig S8. Performance improvement on averaged DMS/AS testing data. This figure shows model performance when we averaged variant scores for DMS or AS data that are: i) published in the same paper; ii) targeting the same protein region; iii) measured by the same type of assays (Supplementary Table 1). The change of prediction ρ for each averaged DMS and AS data pair is shown. A higher value represents higher prediction accuracy achieved when using AS data. Different approaches to filtering/matching the data are shown on the x-axis: "All AS data" used all available data; "Compatibility filtered" used only data of high assay compatibility; "Correlation matched" used only data with the highest regularised correlation for each DMS dataset. Results for data pairs with only one residue are not shown. Notches show the 95% confidence interval around the median, and whiskers show the full value range.

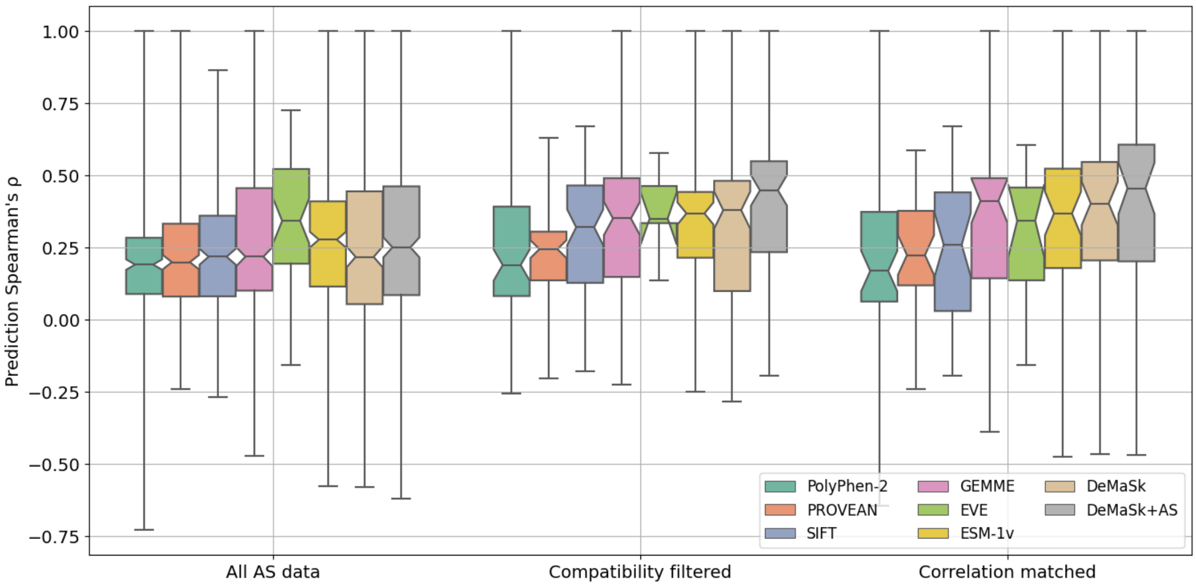

Fig S9. Model performance on various variant effect predictors. The Spearman's ρ between DMS scores and predicted scores from different variant effect predictors for each DMS and AS pair are shown as box plots. Results are evaluated on different sets of variant data shown on the x-axis: "All AS data" used all available data; "Compatibility filtered" used only data of high assay compatibility; "Correlation matched" used only AS data with the highest regularised correlation for each DMS dataset. The figure does not include residues without available AS scores. Results for data pairs with only one residue are not shown. Notches show the 95% confidence interval around the median, and whiskers show the full value range.

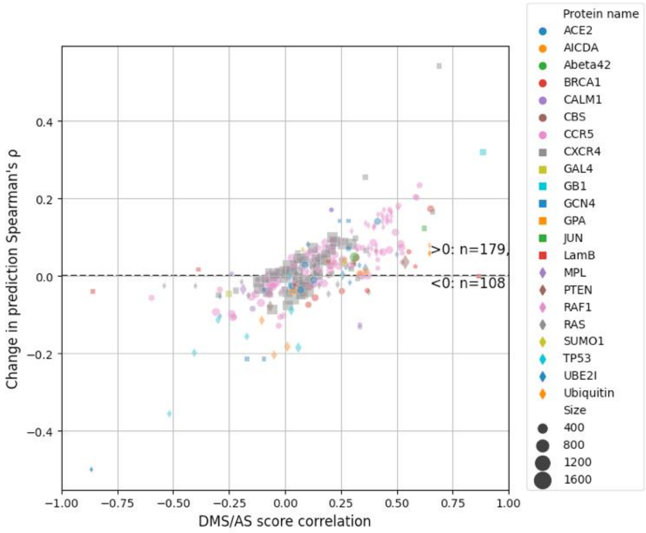

Fig S10. Prediction performance change for using all AS data. Each dot represents a DMS/AS data pair. The vertical axis shows the change of prediction ρ by using AS data (larger means higher performance achieved by

using AS data). The horizontal axis shows the DMS/AS score correlation for *all* variants on the matched residues rather than just alanine substitutions. The colours and shapes of the dots correspond to the target protein, and size indicates the number of variants in each data pair. Results for data pairs with only one residue are not shown.

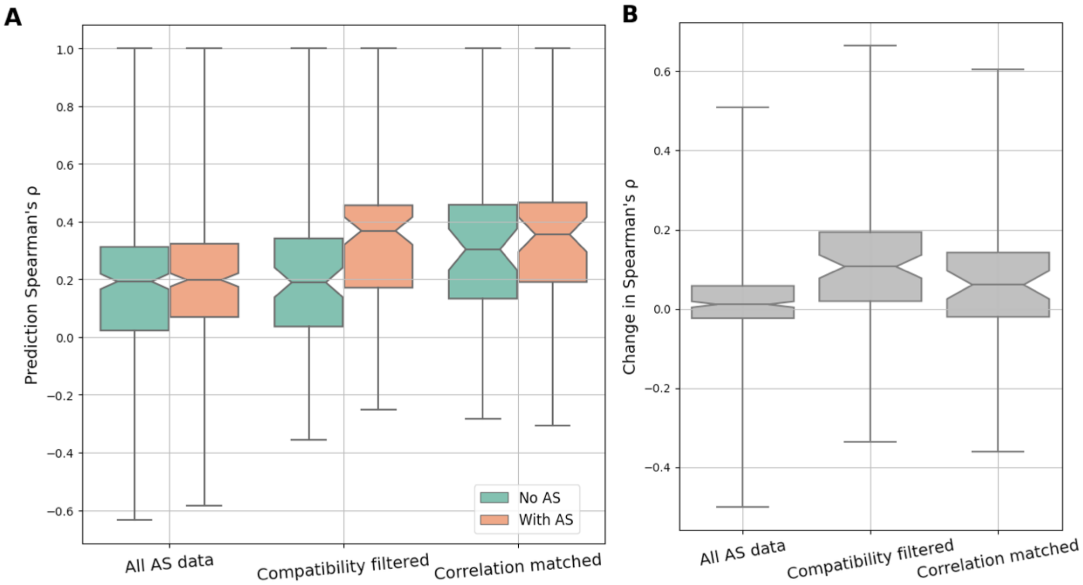

Fig S11. Model performance for training with AS-data-available-residues. The predictors were trained only on variants that have AS data available. Panel A shows the performance visualized by prediction Spearman's ρ for DMS scores and predicted scores for each DMS and AS data pair. Different approaches to filtering the data are shown on the x-axis: "All AS data" used all available data; "Compatibility filtered" used only data of high assay compatibility; "Correlation matched" used only AS data with the highest regularised correlation for each DMS dataset. Control results are shown as green boxes for predictions on the same residues without AS data as a feature. Panel B shows change of prediction ρ for each DMS and AS data pair. A higher value indicates higher prediction accuracy achieved when using AS data. Different approaches to filtering the data are also shown on the x-axis as described. Notches show the 95% confidence interval around the median, and whiskers show the full value range.

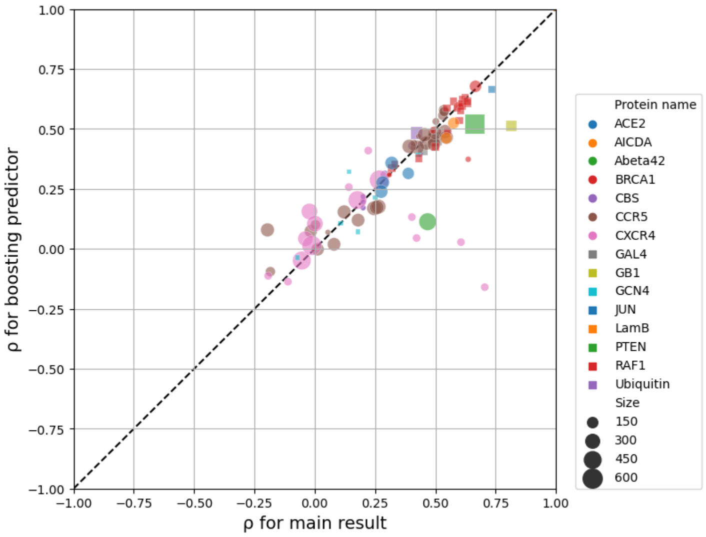

Fig S12. Boosting setup shows similar performance as the main result. Each dot represents a filtered DMS/AS data pair of high assay compatibility. The vertical and horizontal axes show the prediction Spearman's ρ for either modelled with boosting or the one-step (main result) setup. The colours and shapes of the dots correspond to the target protein, and size indicates the number of variants in each data pair.

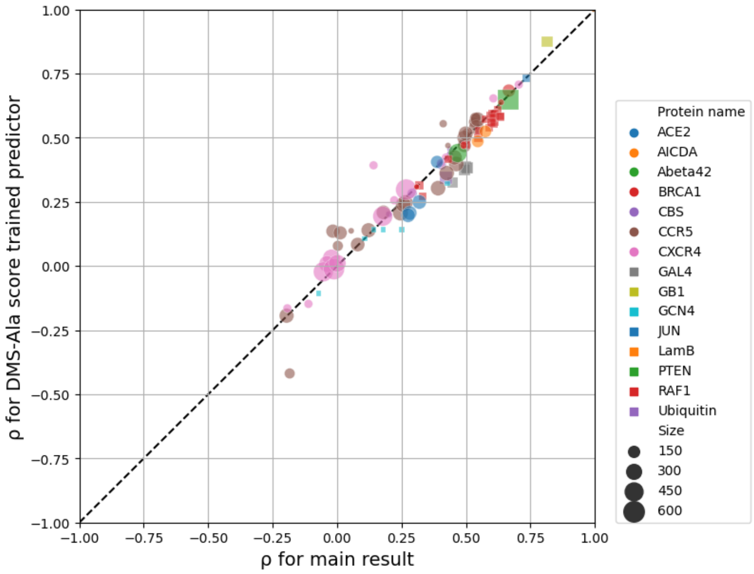

Fig S13. Training with DMS scores of alanine substitutions shows similar performance as the main result. The vertical and horizontal axes show the prediction Spearman's ρ for predictors either trained with DMS score of alanine substitutions (DMS-Ala) or AS data of high assay compatibility (main result), yet all evaluated on high

compatibility AS data. The colours and shapes of the dots correspond to the target protein, and size indicates the number of variants in each data pair.

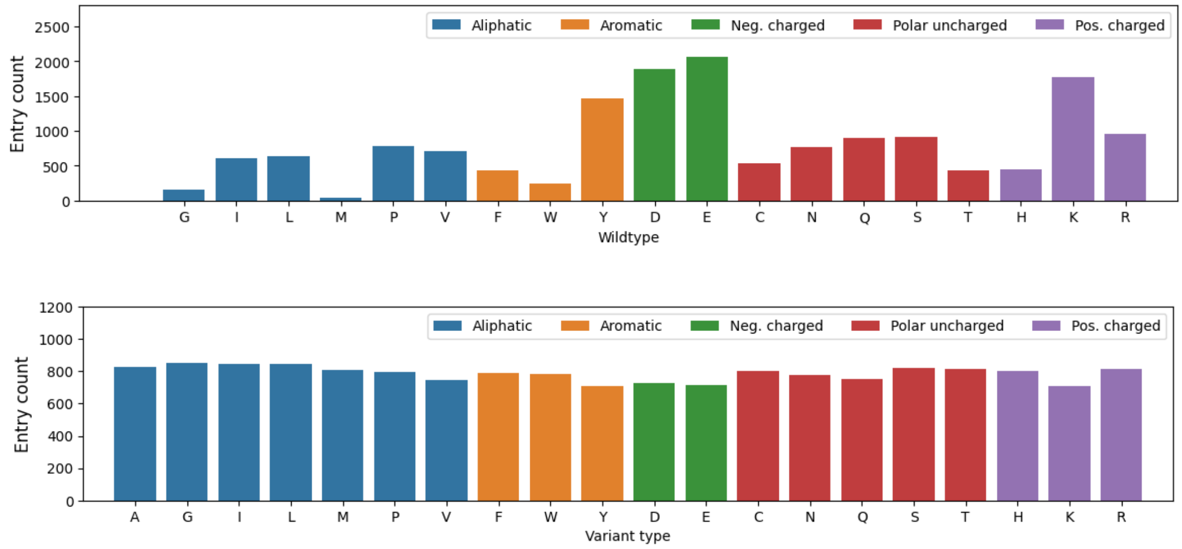

Fig S14. Count of variant entries for each wild-type or variant amino acid of high assay compatibility data. (Neg.: negatively, Pos.: positively)

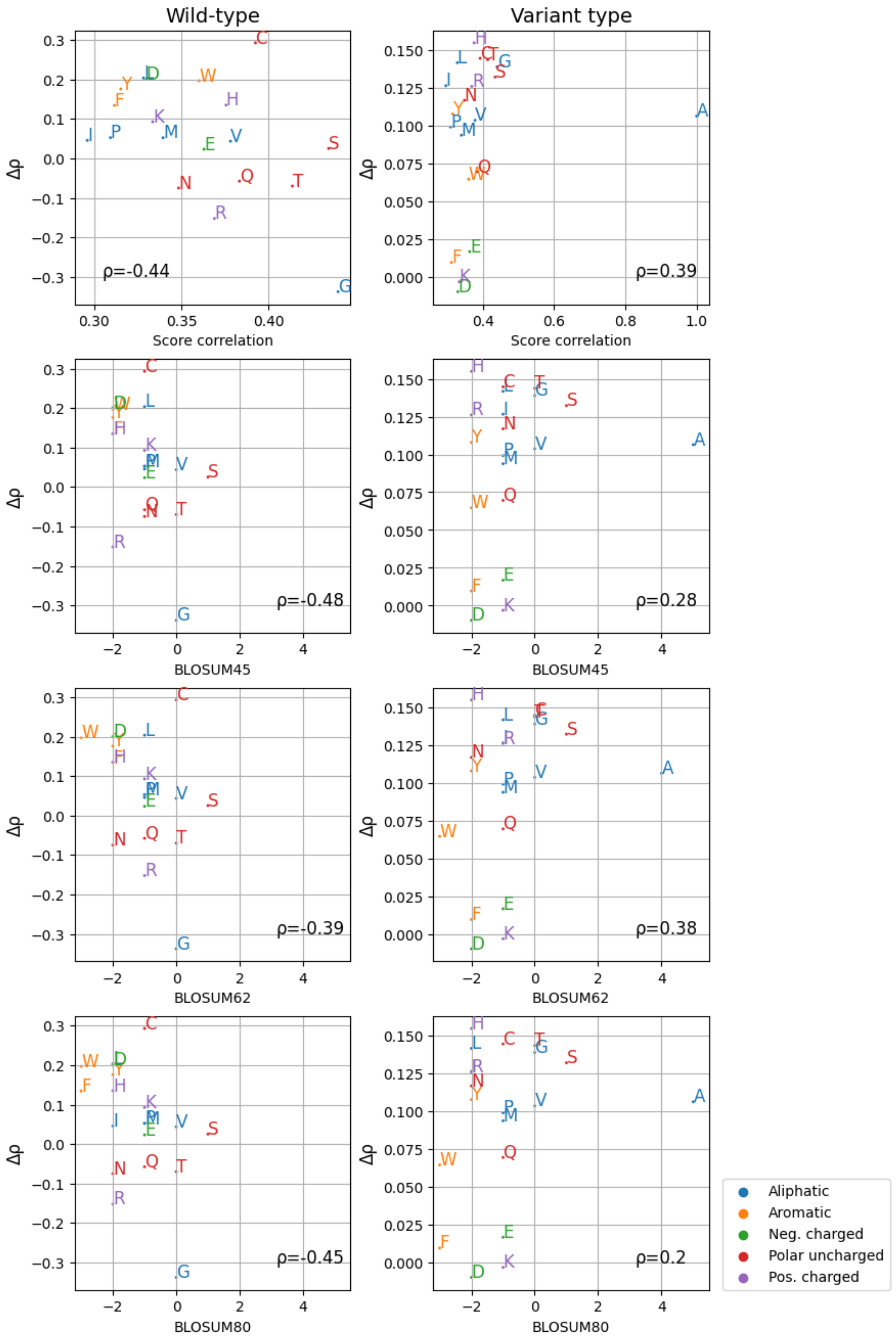

Wild-type
Variant type
Δρ
Score correlation
BLOSUM45
BLOSUM62
BLOSUM80
ρ=-0.44
ρ=0.39
ρ=-0.48
ρ=0.28
ρ=-0.39
ρ=0.38
ρ=-0.45
ρ=0.2
Aliphatic
Aromatic
Neg. charged
Polar uncharged
Pos. charged

Fig S15. Relationship between amino acid similarity and model performance. For each amino acid, its similarity to alanine was computed by their DMS score correlation or using BLOSUM scores as shown on the x-axis. The performance improvement (Δρ) for each wild-type (left) or variant (right) amino acid while using AS data were computed as previously mentioned (Fig 7), with their Spearman's correlation against the similarity measurements shown on the figure. The label for each amino acid is coloured by the amino acid physicochemical property. (Neg.: negatively; Pos.: positively)

Table S1. DMS/AS correlation on each secondary structural region. The secondary structure of each variant is determined by UniProt annotations. The Spearman's correlation between DMS and all or high compatibility AS data on each structural region is computed, with the number of protein residues involved shown in parenthesis.

ρ (n_residues)	HELIX	STRAND	TURN
All AS	0.13 (233)	0.13 (83)	0.17 (22)
AS of high compatibility	0.28 (115)	0.26 (56)	0.41 (15)

Table S2. Amount of data with AS scores available

Data composition	Protein	DMS dataset	AS dataset[1]	Variant entries[2]
All AS	22	54	146	70446
Compatibility filtered	15	35	60	15739
High+medium assay compatibility	21	51	105	28380
Correlation matched	22	54	32	7940

1. This column shows how many unique AS datasets are included.
2. Include duplicated variants caused by multiple experiments targeting the same protein variant.

Supplementary information

Applying AS data to Envision method

We re-implemented a predictor based on Envision [17] to incorporate AS data. Features used in Envision were downloaded from its online toolkit. All Envision features are used for modelling except for substitution type (wt_mut) which has low importance according to the published result and our pilot studies yet is computationally expensive in our setup. Protein data were excluded if their features were not available online. DMS and AS data pairs with high assay compatibility were used for modelling. Missing feature values were imputed by the mean values for numerical features or the most frequent values for categorical features. Categorical features are encoded with the one-hot encoder. We used `sklearn.ensemble.GradientBoostingRegressor` from scikit-learn package [130] to build the predictor, and hyperparameters were tuned by Bayesian Optimization [139] with Group K-Fold (protein-30-fold) cross-validation. The training and evaluation process were similar to that previously described. For comparison, we repeated the DeMaSk-based analysis on the same subset of data.

Boosting with AS data

To deal with the sparsity of AS data, we tested a variant impact predictor based on boosting. A first linear regression predictor was trained with all training DMS data using the three DeMaSk features without AS data, which was the same as the control predictor mentioned previously. We then calculated the prediction error by subtracting the predicted scores from DMS scores, and a second linear regression predictor was trained to predict the error. The second predictor was trained only on DMS/AS data of high assay compatibility and used both protein features and the encoded AS scores. The final prediction result was the sum of the outputs from these two predictors.

Replacing AS data with DMS scores of alanine substitutions

We investigated another potential approach to overcome the sparsity of AS data by replacing the AS feature with the DMS scores of alanine substitutions (DMS-Ala). The intention of this study is to model the scenario of ideal AS data, which perfectly matches the DMS-Ala data during training. To do this, for all DMS datasets we collected, their AS feature values, regardless of availability, were replaced by the DMS-Ala scores on the same residue. Missing scores were imputed by the mean value of all DMS-Ala scores. A regression model was trained and evaluated as previously described, using the three DeMaSk features as well as the DMS-Ala scores. The AS data of high assay compatibility are still used for the testing process.

 Click here to access/download;Manuscript;Review_response_untracked.pdf

Reviewer 1 - Joseph Ng

This manuscript explored whether low-throughput alanine scanning (AS) experimental data could complement deep mutational scanning (DMS) to classify the impact of amino acid substitutions in a range of protein systems. The analysis partially confirms this hypothesis in that it only applies when the functional readout being measured in the two assays are compatible with one another.
In my opinion this is an insight that should be highlighted in a publication and therefore I believe this manuscript deserved to be published. I just wish the authors could clarify & further explore the points below better in their manuscript before recommending for acceptance:

1. In my opinion the most important bit of data curation is the classification of DMS/AS pairs as high/medium/low etc. compatible, and this is the key towards the authors' insight that assay compatibility is an important determinant of whether signals in the two datasets could be cross-matched for analysis. The criteria behind this classification are listed in Figure S2 but I feel the wording needs to be more specific. For example, in Figure S2, the authors wrote 'Both assays select for similar protein properties and under similar conditions' - what exactly does this mean? What does the authors consider to be 'similar protein properties'? I could not find more detailed explanation of this in the Methods section. The authors gave reasons in the spreadsheet in Supp. Table 1 for the labels they give to each pairs of assays, but I'm still not exactly sure what they consider to be 'similar'. Is there are more specific classification scheme which is more explicit in defining these 'similarities', e.g. by defining a scoring grid explicitly listing the different levels of 'similarities' of measurable properties, e.g. both thermal stability - score of 3; thermal stability vs protein abundance - 2; thermal stability vs cell survival - 1 (or equivalent, I think the key issue is to provide the reader with a clear guide so they can readily assess the compatibility of the datasets by themselves)?

Response:
Thank you for the comment. In the Methods section, we added a detailed explanation about which assays are similar (line 335):

> *We categorized each DMS or AS assay by the protein property or function using the following assay types: binding affinity, enzyme activity, protein abundance, cell survival, pathogen infection, drug response, ability to perform a novel function, or other protein-specific*

activities (e.g., transcription activity for transcription factors) (Supplementary Table 1).

We also refined Fig S2 and its legend (also shown below) corresponding to the updated Methods. In the Supp. Table 1, we added the DMS and AS categories beside the reasons of compatibility classifications.

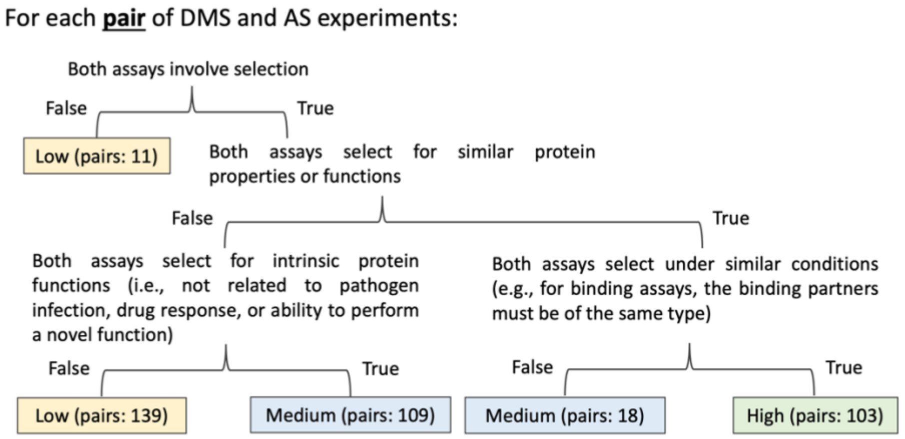

Fig S2: Decision tree for classifying DMS and AS assay compatibility. *The similarity of DMS and AS assays are compared (Methods) and the DMS/AS assay pairs are classified using three levels of compatibility (low, medium, high).* The leaf –node text and color show the classified assay compatibility. The number indicates the count of assay pairs for each compatibility level.

2. I would have thought discrepancy between the DMS and AS scores to be different across different structural regions of the protein, e.g. the discrepancy would be larger in ordered region compared to disorder as the protein fold would constrain the types of amino acids tolerable within the ordered segment of the protein. Is this the case in the authors' collection of datasets? If so, does the compatibility of assays modulate this discrepancy?

Response:

Thank you for this suggestion. To explore this, we annotated the structural regions for our variants using UniProt. We found only one protein in our dataset that has alanine scanning data available in a disordered protein region. This makes it impractical for us to analyse the ordered/disordered score discrepancy.

We next grouped variants by secondary structure (HELIX, STRAND and TURN) and calculated the Spearman's ρ between all DMS scores and AS scores as shown in Table S1 (also below). We found that TURN regions tended to have high score correlation compared to HELIX and STRAND, and this trend was stronger for DMS/AS data with high assay compatibility. We added a description of this result in the main text (line 120):

This trend of increased correlation for high compatibility assay pairs holds across secondary structures (Table S1).

ρ (n_residues)	HELIX	STRAND	TURN
All AS	0.13 (233)	0.13 (83)	0.17 (22)
AS of high compatibility	0.28 (115)	0.26 (56)	0.41 (15)

Table S1. DMS/AS correlation on each secondary structural region. The secondary structure of each variant is determined by UniProt annotations. The Spearman's correlation between DMS and all or high compatibility AS data on each structural region is computed, with the number of protein residues involved shown in parenthesis.

Reviewer 2 - Leopold Parts

Summary
Fu et al. explore utilising low-throughput mutational fitness measurements to predict the results of high-throughput deep mutational scanning experiments. They demonstrate that adding alanine scanning results to predictive models improves performance, as long as the alanine scan used a sufficiently similar evaluation approach to a deeper experiment. The findings make intuitive sense, and will be useful for the community to internalize.

While we have several comments about the methods used, and requests to fortify the claims with more characterization, we do not expect addressing any of them will change the core findings. One can argue that direct application of AS boosted predictions is likely to be limited due to the number of scans available and the speed at which DMS experiments are now being performed, so it would also be useful to discuss the context of these results in the evolution of the field, and we make specific suggestions for this. Regardless, the presented results are a useful demonstration of a more general use case of low-throughput or partial mutagenesis data for improving fitness prediction and imputation.

Response:
Thank you for the feedback. We have added extra context information about related research, strengthening the motivation and contribution of this study. Details can be found in the responses to the first minor comment below.

Major Comments

*	There are many other computational variant effect predictors beyond Envision and DeMaSk. It would be very useful to see how their prediction results compare to some others, particularly the best performing and common models that are also straightforward to download and run (e.g. EVE, ESM1v, SIFT, PolyPhen2). This would be important context to see how impactful the addition of AS data is to DeMaSk/Envision. Please run additional prediction tools for reference of absolute performance; there is no need to incorporate AS data into them.

Response:

Thank you for this comment. We avoided doing this initially because our primary motivation was to investigate if AS data can improve predictors, leading us to focus on the improvement rather than absolute performance achieved.

We agree that benchmarking other predictors is useful to help readers contextualize performance and improvement. As suggested, we ran several of these predictors locally or online and compared with our results as shown in Fig S9 (also shown below). These predictors were run with default settings. We found it hard to run EVE locally since it requires substantial GPU resources. Instead, we collected pre-calculated EVE prediction results (details in Method section, line 387). A brief explanation of this result can be found in the main text (line 176). Specifically:

> *Our compatibility-filtered predictor shows improved prediction accuracy for these regions compared to not only the baseline model, but other widely used predictors as well.*

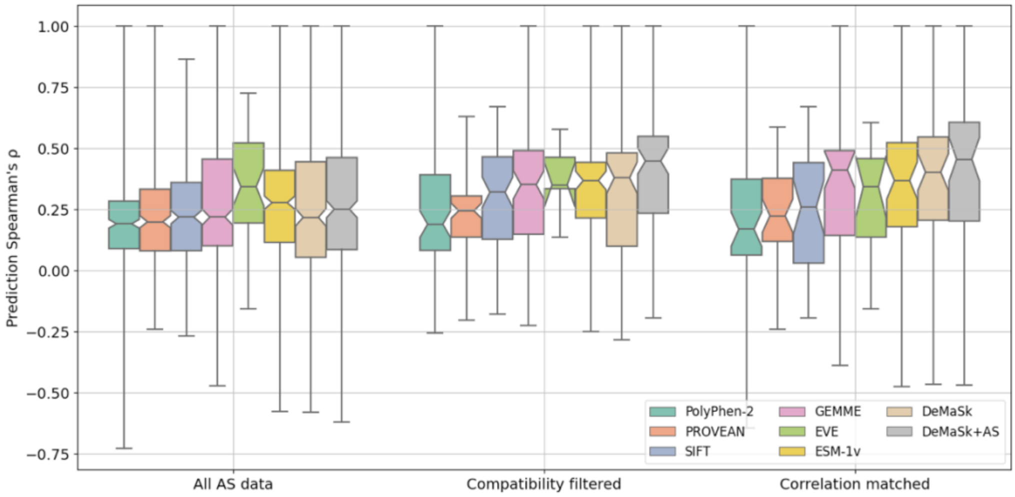

Fig S9. Model performance on various variant effect predictors. The Spearman's ρ between DMS scores and predicted scores from different variant effect predictors for each DMS and AS pair are shown as box plots. Results are evaluated on different sets of variant data shown on the x-axis: "All AS data" used all available data; "Compatibility filtered" used only data of high

assay compatibility; "Correlation matched" used only AS data with the highest regularised correlation for each DMS dataset. The figure does not include residues without available AS scores. Results for data pairs with only one residue are not shown. Notches show the 95% confidence interval around the median, and whiskers show the full value range.

* Several proteins have a very small number of AS residues (Figure 2), and from our reading of the methods, other residue scores are imputed with the mean AS value for that protein. (As an aside, it would be good to clarify if this average is across studies or within study). If this reading is correct, the majority of residues for each proteins will have imputed AS results (e.g. in case of PTEN, over 90%), which can be problematic for training and prediction. Please clarify if our interpretation of the imputation approach is correct, and if so, please also provide results for a model trained without imputation, on many fewer residues. If the boosting model has already implemented this, please integrate the Supplementary methods into the main methods, and reference these and the results when describing the imputation approach to avoid such concerns.

Response:
This interpretation is correct. The missing AS values are imputed by the average score across all studies. We have emphasized this statement by adding (line 356):

> AS scores were imputed with the mean value of all available AS scores *across all studies*.

The boosting model is related to this yet not directly answering the question at hand. So, here we add the modelling results for training without imputed variants, i.e., just on the AS-available residues, as shown in Fig S11 (also shown below). The models show similar pattern of improvement (Fig S11 B) compared to the main results in Fig 5 (also shown below). However, the absolute prediction performance (Fig S11 A) is worse than the main results in Fig S4 (also shown below), likely because the models are trained on a much smaller number of variants. We added several sentences in the manuscript referring to this result (line 195-200):

> We also explored the consequences of the sparsity of AS data on our model in three ways: *i) by training only with variants that have AS data available; … The first approach gave lower absolute prediction performance, presumably because the model was under-fitted due to the small number of variants*.

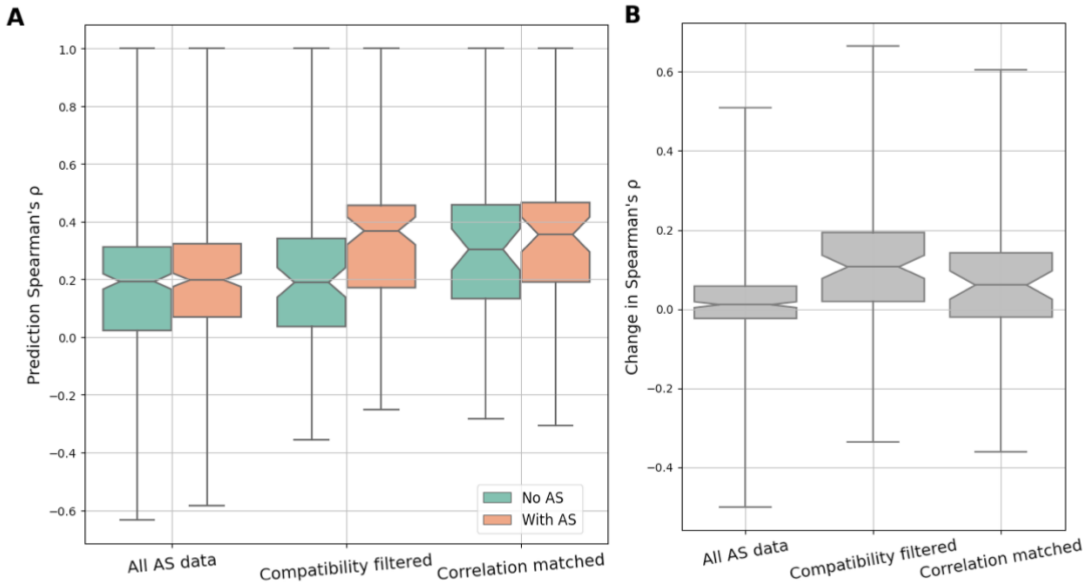

Fig S11. Model performance for training with AS-data-available-residues. The predictors were trained only on variants that have AS data available. Panel A shows the performance visualized by prediction Spearman's ρ for DMS scores and predicted scores for each DMS and AS data pair. Different approaches to filtering the data are shown on the x-axis: "All AS data" used all available data; "Compatibility filtered" used only data of high assay compatibility; "Correlation matched" used only AS data with the highest regularised correlation for each DMS dataset. Control results are shown as green boxes for predictions on the same residues without AS data as a feature. Panel B shows change of prediction ρ for each DMS and AS data pair. A higher value indicates higher prediction accuracy achieved when using AS data. Different approaches to filtering the data are also shown on the x-axis as described. Notches show the 95% confidence interval around the median, and whiskers show the full value range.

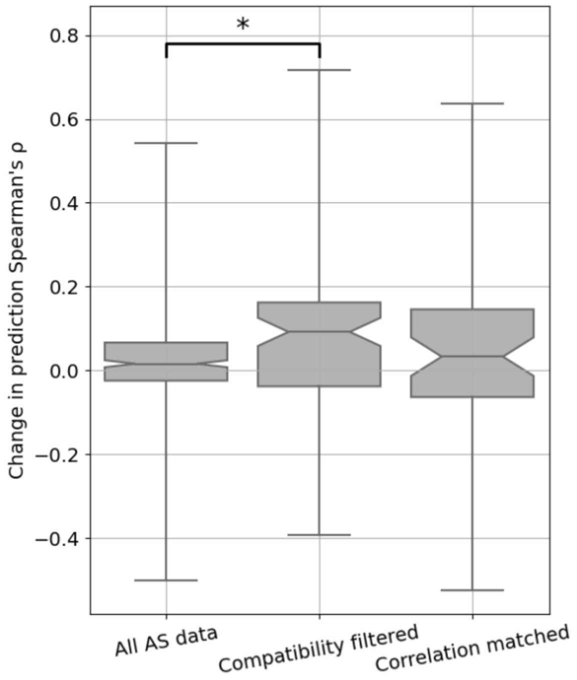

Fig 5. Performance of variant impact prediction is improved using AS data with high assay compatibility. The change in prediction ρ achieved by including the AS data feature for each DMS and AS data pair is shown as box plots. A higher

value represents higher prediction accuracy achieved for using AS data. Different approaches to filtering/matching the data are shown on the x-axis: “All AS data” used all available data; “Compatibility filtered” used only data of high assay compatibility; “Correlation matched” used only data with the highest regularised correlation for each DMS dataset. Results for data pairs with only one residue are not shown. P-values were calculated using Welch’s test and jointly corrected using Holm-Šidák (Methods), *: p<0.05. Notches show the 95% confidence interval around the median, and whiskers show the full value range.

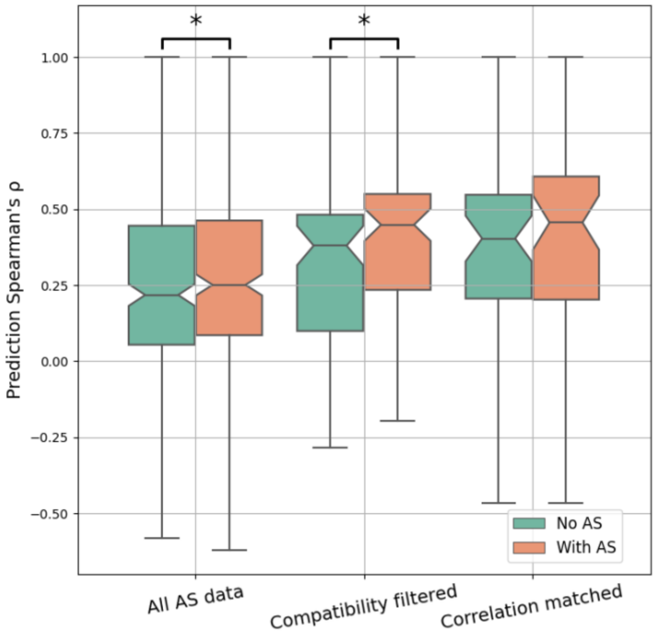

Fig S4. Performance comparison between predictors with or without AS data. The Spearman’s ρ between DMS scores and predicted scores for each DMS and AS data pair are shown as box plots. Different approaches to filtering the data are shown on the x-axis: “All AS data” used all available data; “Compatibility filtered” used only data of high assay compatibility; “Correlation matched” used only data with the highest regularised correlation for each DMS dataset. The figure does not include data without available AS scores. This means that the different results are not directly comparable since they are computed for different subsets of DMS/AS data pairs (for example, “All AS data” contains all DMS/AS data pairs, but “Compatibility filtered” contains only data pairs of high assay compatibility). Control results are shown as green boxes for predictions on the same residues without AS data as a feature. The underlying ρ for each data pair in the control results is the same, but the boxes are shifted due to data filtering. Results for data pairs with only one residue are not shown. P-values were calculated using paired t-test and jointly corrected using Holm-Šidák (Methods), *: p<0.05. Notches show the 95% confidence interval around the median, and whiskers show the full value range.

* It is not clear how significant/impactful the increases in performance are in figures 4, 5, S4, S5 & S6. Please use a reasonable analytical test, or training data randomization to evaluate the improvement against a null model.

Response:

We initially used notched box plots to indicate the confidence interval (95%) of the median and now emphasize this in the legend for each box plot with extra description in Methods (line 384):

> *The 95% confidence interval of median values are calculated by Gaussian-based asymptotic approximation.*

We have also added statistical tests to these figures (also shown below). For Figure 4, we used Welch's test to compare the mean values between each compatibility group (High vs. Medium, High vs. Low, High vs. Overall, Medium vs. Low, …), and corrected for multiple testing using the Holm–Šidák method. This is now described in the figure legend (see below).

For Figures 5 and S5, we also used Welch's test to compare the mean improvement between each pair of models. For Figures S4 and S6, we used paired t-tests to compare if using AS data improves the prediction correlation for each model. Since results in these four figures are highly related, their statistical results are corrected jointly using the Holm–Šidák method. These figures have been updated in the manuscript (summarized below) with brief explanations added to the figure legends. A description of the statistical tests was added to the Methods (line 381):

> *Model performance was compared using the following statistical tests. Results in Fig 5 & S5 were tested with Welch's test, and results in Fig S4 & S6 were tested with paired t-tests. The p-values were jointly corrected using the Holm–Šidák method.*

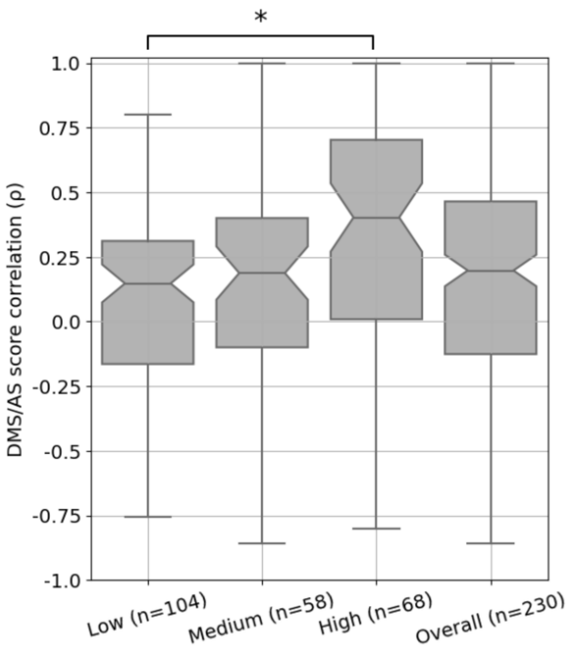

Fig 4. DMS and AS data pairs with high assay compatibility show a higher score correlation. Each box shows the Spearman's ρ between DMS and AS data pairs for each level of assay compatibility or overall. The correlation coefficients

were calculated between alanine substitution scores in each pair of AS and DMS datasets. Results for pairs with less than three alanine substitutions were removed. *P-values calculated using Welch's test and corrected using Holm-Šidák, *: p<0.05; notches show 95% confidence interval around median, and whiskers show the full value range.*

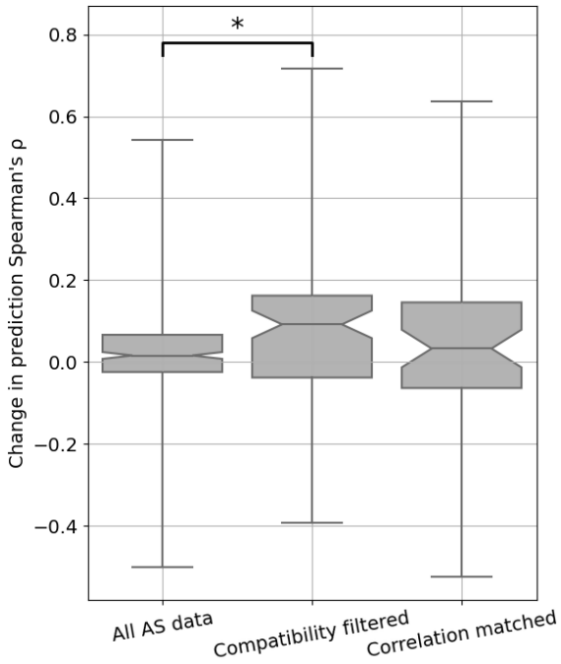

Fig 5. Performance of variant impact prediction is improved using AS data with high assay compatibility. The change in prediction ρ achieved by including the AS data feature for each DMS and AS data pair is shown as box plots. A higher value represents higher prediction accuracy achieved for using AS data. Different approaches to filtering/matching the data are shown on the x-axis: "All AS data" used all available data; "Compatibility filtered" used only data of high assay compatibility; "Correlation matched" used only data with the highest regularised correlation for each DMS dataset. Results for data pairs with only one residue are not shown. *P-values were calculated using Welch's test and jointly corrected using Holm-Šidák (Methods), *: p<0.05. Notches show the 95% confidence interval around the median, and whiskers show the full value range.*

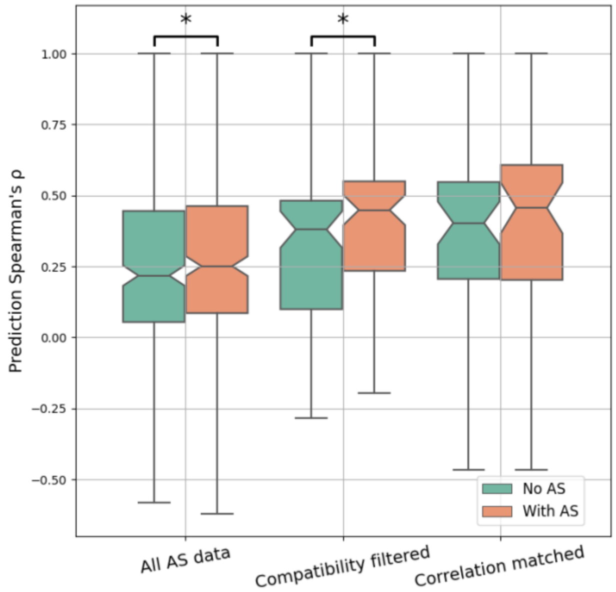

Fig S4. Performance comparison between predictors with or without AS data. The Spearman's ρ between DMS scores and predicted scores for each DMS and AS data pair are shown as box plots. Different approaches to filtering the data are shown on the x-axis: "All AS data" used all available data; "Compatibility filtered" used only data of high assay compatibility; "Correlation matched" used only data with the highest regularised correlation for each DMS dataset. The figure does not include data without available AS scores. This means that the different results are not directly comparable since they are computed for different subsets of DMS/AS data pairs (for example, "All AS data" contains all DMS/AS data pairs, but "Compatibility filtered" contains only data pairs of high assay compatibility). Control results are shown as green boxes for predictions on the same residues without AS data as a feature. The underlying ρ for each data pair in the control results is the same, but the boxes are shifted due to data filtering. Results for data pairs with only one residue are not shown. *P-values were calculated using paired t-test and jointly corrected using Holm-Šidák (Methods), *: p<0.05. Notches show the 95% confidence interval around the median, and whiskers show the full value range.*

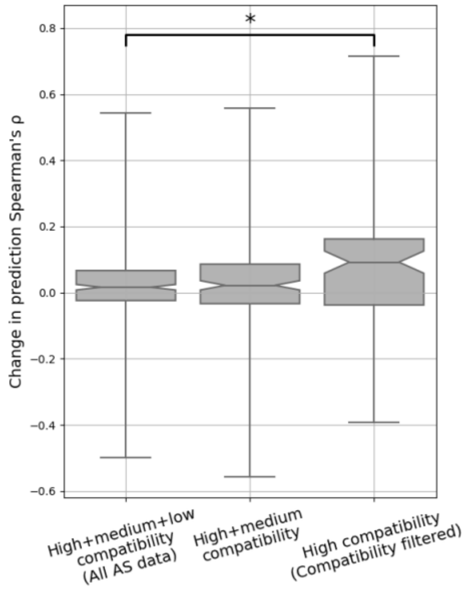

Fig S5. The change in prediction performance for using data of different assay compatibility levels. The change of prediction Spearman's ρ for each DMS

and AS data pair is shown as box plots. A higher value represents higher prediction accuracy achieved for using AS data. Different data filtering methods are shown on the x-axis. Results for data pairs with only one residue are not shown. *P-values were calculated using paired t-test and jointly corrected using Holm-Šidák (Methods), *: p<0.05. Notches show the 95% confidence interval around the median, and whiskers show the full value range.*

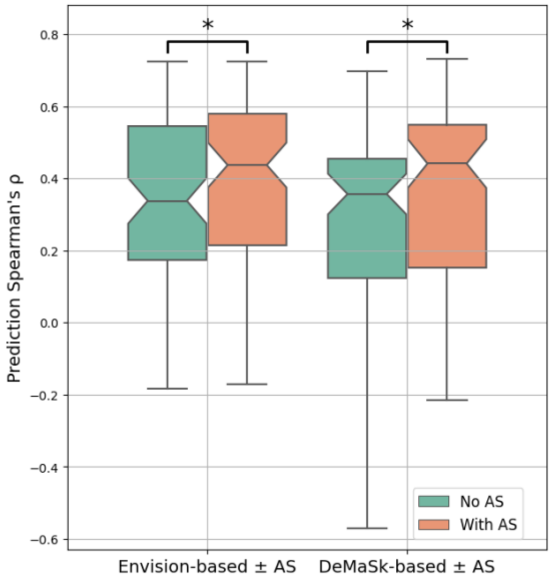

Fig S6. Prediction performance is improved while incorporating high compatibility AS data into the Envision model. The Spearman's ρ between experiment DMS scores and predicted scores for each DMS/AS assay pair with high compatibility are shown as box plots. The x-axis shows the predictor used, either Envision or DeMaSk. Control results are shown as green boxes for predictions on the same residues without AS data as a feature. Results for data pairs with only one residue are not shown. *P-values were calculated using paired t-test and jointly corrected using Holm-Šidák (Methods), *: p<0.05. Notches show the 95% confidence interval around the median, and whiskers show the full value range.*

* There are quite a few proteins with repeated DMS/AS measurements. In our experience these correlate from moderately to very highly. Including multiple highly correlated studies could lead to pseudo-replication and biasing the model performance results. Please present a version of the results where the repeats are averaged first to test whether that bias exists.

Response:

We indeed observed multiple cases where one protein might have several DMS or AS experiments available. While training the models, to avoid potential bias, we weighted each protein variant equally to compensate for certain regions having greater coverage with DMS and AS assays. This process was equivalent to averaging variant scores in the training data.

But during the evaluation process, we agree that these pseudo-replicates may still exist.

Here we averaged DMS and AS results from experiments that: 1. Were published in the same paper; 2. Targeted the same protein region; 3. Used the same type of assays (binding affinity, enzyme activity, protein abundance, cell survival, etc.). Then we evaluated the model performance on averaged datasets, as shown in Fig S8 (also shown below). We observe a similar pattern of improvement to the main result (Fig. 5 also shown below) and have added this result to the main text (line 162):

> *Additionally, to ensure the models performance is not biased by pseudo-replication of multiple datasets, we averaged DMS and AS scores that were part of the same study and type of assay, and saw similar results (Fig S8)*.

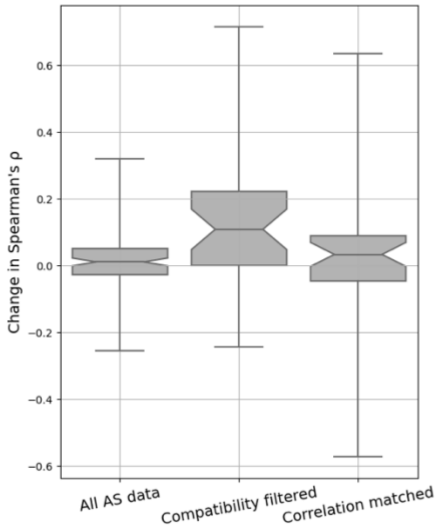

Fig S8. Performance improvement on averaged DMS/AS testing data. This figure shows model performance when we averaged variant scores for DMS or AS data that are: i) published in the same paper; ii) targeting the same protein region; iii) measured by the same type of assays (Supplementary Table 1). The change of prediction ρ for each averaged DMS and AS data pair is shown. A higher value represents higher prediction accuracy achieved when using AS data. Different approaches to filtering/matching the data are shown on the x-axis: "All AS data" used all available data; "Compatibility filtered" used only data of high assay compatibility; "Correlation matched" used only data with the highest regularised correlation for each DMS dataset. Results for data pairs with only one residue are not shown. Notches show the 95% confidence interval around the median, and whiskers show the full value range.

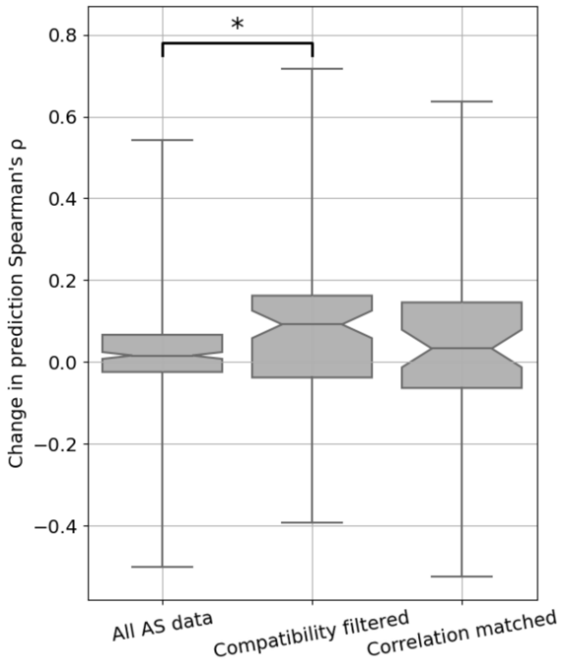

Fig 5. Performance of variant impact prediction is improved using AS data with high assay compatibility. The change in prediction ρ achieved by including the AS data feature for each DMS and AS data pair is shown as box plots. A higher value represents higher prediction accuracy achieved for using AS data. Different approaches to filtering/matching the data are shown on the x-axis: "All AS data" used all available data; "Compatibility filtered" used only data of high assay compatibility; "Correlation matched" used only data with the highest regularised correlation for each DMS dataset. Results for data pairs with only one residue are not shown. P-values were calculated using Welch's test and jointly corrected using Holm-Šidák (Methods), *: $p<0.05$. Notches show the 95% confidence interval around the median, and whiskers show the full value range.

Minor Comments [suggestions only; no analyses required from us]

* A short discussion about the number of available alanine scans, particularly for proteins without DMS results, would help put the work in context. For example, it would be good to know how many proteins would benefit from improved de-novo predictions (e.g. no DMS data) and how many could have improved imputation (incomplete DMS data). Similarly the rate and cost of DMS data generation is important to understand the utility of their results. I think a short discussion of how useful models of this sort are in practice now and in future would be helpful to the reader. This seems most natural as part of the end of the discussion, but could also fit in the introduction.

To our knowledge, there is no well-established database for AS datasets, so, it is challenging to determine how many proteins and which regions have AS data available. But we did find some papers talking about DMS data availability and we added a sentence in the introduction (line 43):

So far, hundreds of DMS studies covering tens of thousands of nucleotides have been published <PMID 36055970>, and experiments targeting over a hundred additional genes are underway according to MaveRegistry <PMID 33774657>.

To discuss the utility of variant effect predictors, we also added the following sentence (line 56):

These variant effect predictors can also be benchmarked using DMS experimental results and to assist the interpretation of experimental data.

* Figure 2 is missing y axis label. We also softly suggest log scale axis, to not obscure the degree to which some proteins have more residues covered and the proportion of residues covered by AS.

We added y axis label to Figure 2. We also tried to use log scale as shown below. However, the purpose of this figure is to demonstrate the proportion of residues that have AS data available, which we think is not best demonstrated on log scales.

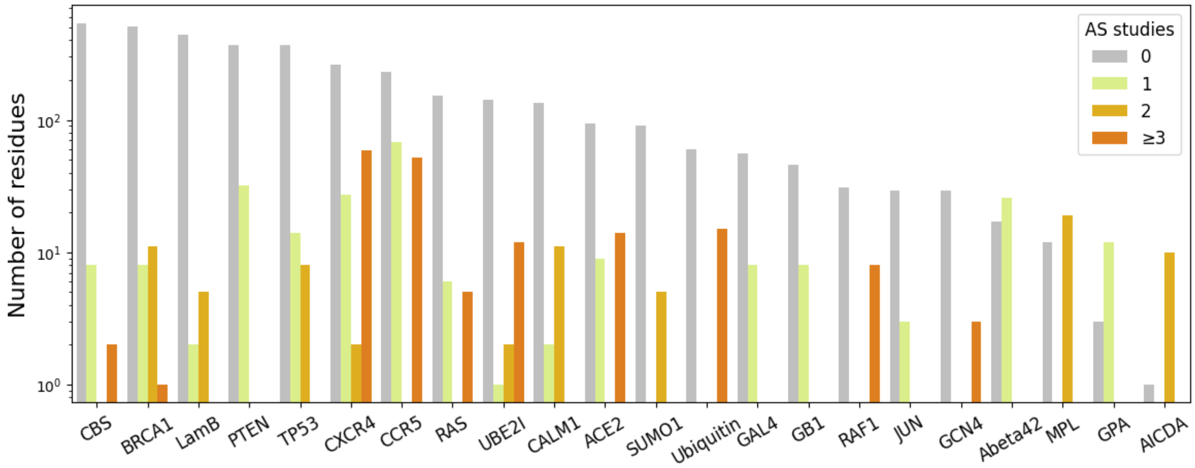

AS data coverage on DMS scanned region (log-scaled). The horizontal shows different proteins. Each bar represents the number of residues assayed by a given number of AS studies.

* Figure 3 includes DMS/AS study pairs with at least three alanine substitutions to compare - we think this is a low cut-off, particularly with the regularisation applied. I think something like 10+ would be more informative.

We explored setting the threshold to 10 (left) or 15 (middle) and the result is shown below. It seems quite similar to Fig 3 in the main text, shown below (right), with similar distribution and median value but indeed with less extreme values. Since, the purpose of this figure is to

give a general idea of how the datasets are correlated, we are inclined to include more datasets in the analysis.

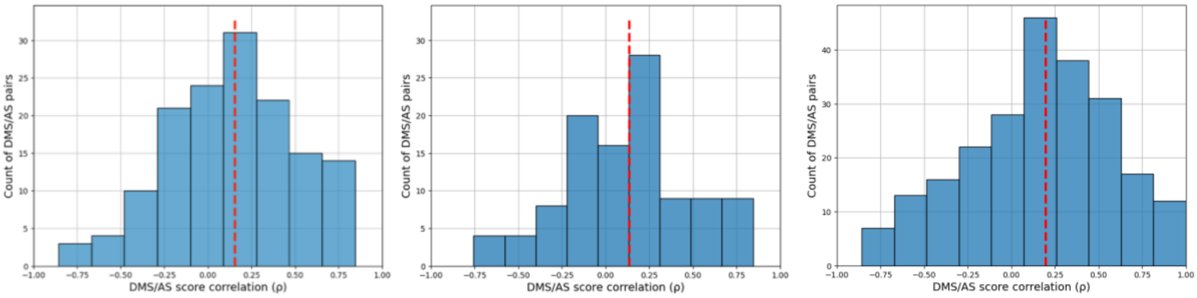

Correlation between DMS and AS data shows substantial variation. We calculated Spearman's ρ between alanine substitution scores in each pair of AS and DMS data. The results for pairs with less than 10 (left), 15 (middle) or 3 (right) alanine substitutions are not shown. The red dashed line shows the median ρ.

*	I think their cross-validation scheme leaves out an entire protein at a time, as opposed to one study each iteration. I agree this is the better way to do it. However, I initially read it as the latter, which would lead to leakage between train/validation data since the same residue would be included in both if a protein had multiple datasets. It might be useful to be more explicit to prevent other readers doing the same.

Thank you for the comment. We have now clarified this in the text (line 140):

> We applied a leave-one-protein-out cross-validation approach to training and testing, *avoiding information leakage for variants of the same protein target.*

*	L231 In the discussion they mention fitting a model only using studies with a minimum DMS/AS correlation. This occurred to me as well while reading the relevant part of the results. Is there a good reason not to do this? It doesn't seem like a large amount of work and conceptually seems a good way to assess a model that says what a DMS might look like is it had the same selection criteria as a given AS.

While doing the correlation matching, we additionally set thresholds for the regularized correlation values of 0, 0.25, 0.5. The result, together with the original ones, are shown in Fig S7 (also shown below), indicating that constraining the regularized correlation to be larger than 0 and 0.25 will give higher median improvement compared to the original correlation matching result. However, performance drops when using threshold of 0.5. The likely explanation of this is that many datasets are discarded. We added one sentence in the main text referring to this result (line 161):

However, when applying a stricter threshold, the correlation matched models still show limited improvement.

And the original sentence in L231 was deleted.

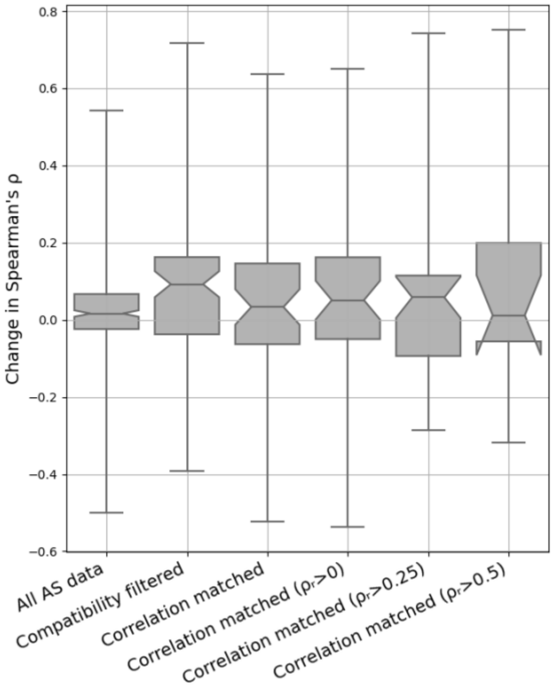

Fig S7. Performance improvement on thresholded correlation matching. The change of prediction ρ for each DMS and AS data pair is shown as box plots. Different approaches to filtering/matching the data are shown on the x-axis: "All AS data", "Compatibility filtered" and "Correlation matched" are the same results as previously discussed; while doing correlation matching, a further thresholding (0, 0.25 or 0.5) on the regularized DMS/AS correlation values (ρ_r) was applied. Notches show the 95% confidence interval around the median, and whiskers show the full value range.

* L154 Similarly, a correlation cut-off as well as choosing the most corelated study seems like it would be a fairer comparison in figure 5. Just because an AS is the most correlated doesn't necessarily mean it is well correlated.

Please see the response above.

* It would be interesting to see if the improvement results in figure 7 correlate with substitution matrices (e.g. Blosum) or DMS variant fitness correlations (e.g. correlation between A and C, A and D, etc.). Intuitively it feels like they should.

Here we tried to demonstrate how amino acid similarity between alanine and a certain amino acid (measured by DMS score correlation or BLOSUM scores) conforms with the improvement of using AS data (Δρ), when the wild-type or variant type is the amino acid. For DMS score correlation,

the correlation between DMS score of alanine (A) substitutions against all other amino acids (C, D, …) were computed ($\rho_{A,C}$, $\rho_{A,D}$, …). For BLOSUM scores, we used BLOSUM 45, 62 and 80 for alanine versus other amino acids ($\beta_{A,C}$, $\beta_{A,D}$, …). The performance improvement for each wild-type or variant amino acid while using AS data were taken from the data underlying Figure 7.

These results are visualized in Fig S15 (also shown below). These similarity metrics show negative correlation with $\Delta\rho$ on each wildtype amino acid (left), indicating if the wildtype amino acid is similar to alanine, then AS is not helpful probably because it gives less information. On the other hand, if the variant amino acid is similar to alanine, then AS helps to improve prediction accuracy (right).

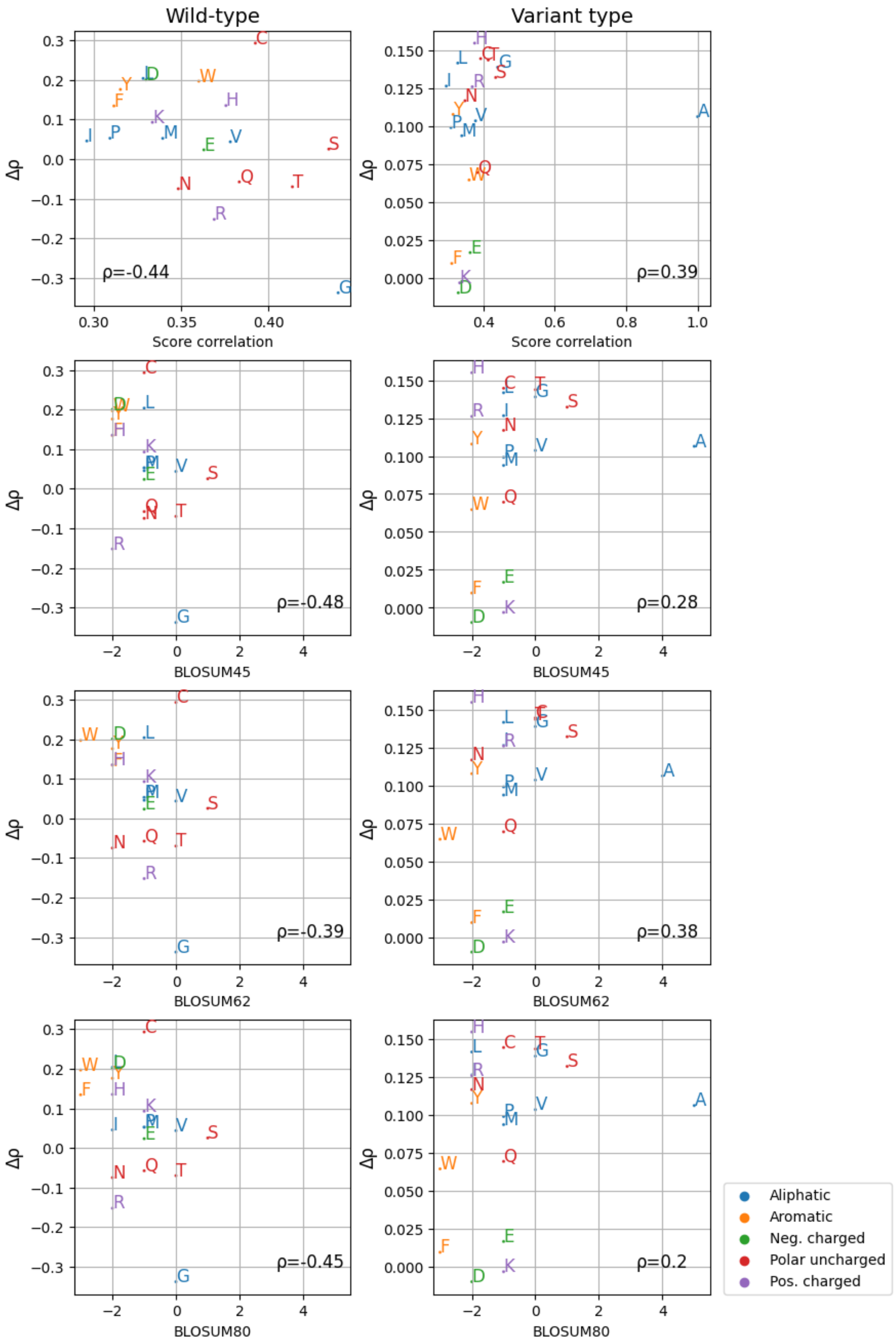

Fig S15. Relationship between amino acid similarity and model performance. For each amino acid, its similarity to alanine was computed by their DMS score correlation or using BLOSUM scores as shown on the x-axis. The performance improvement (Δρ) for each wild-type (left) or variant (right) amino acid while using AS data were computed as previously mentioned (Fig 7), with their Spearman's correlation against the similarity measurements shown on the figure.

The label for each amino acid is coloured by the amino acid physicochemical property. (Neg.: negatively; Pos.: positively)

* It would be nice to label panels in figure 7.

Thank you for pointing out this oversight. We have added panel labels to Figure 7.

* It also seems notable that predicting alanine substitutions is not the most improved - a brief comment on why would be interesting.

We added a sentence on line 209, saying:

> *We also noticed that variants to alanine are not most improved, however we observed an overall trend showing higher improvement for amino acids that are physiochemically similar to alanine (Fig S15).*

* The AS model adds 2x20 parameters to the model for encoding, which is a lot if CCR5 is held out, as there are only a few hundred total independent residues evaluated. While the performance on held out proteins is a good standard, it would be interesting to evaluate the increase from model selection perspective (BIC/AIC or similar) if possible.

Thank you for this suggestion. It's an interesting way to determine if the extra AS data contributes to better predictor performance. However, there are some difficulties for applying this method to our analysis. First, AIC/BIC analysis considers all data at the same time and does not perform cross-validation, meaning we would have to re-run all our models. Second, as a model selection approach, it is more reasonable to compare AIC/BIC values for models without each protein feature rather than AS data alone, which is beyond the scope of this work. Finally, because of the sparsity of AS data, many protein residues have no AS data available, and we anticipate that AIC/BIC analysis will lose power in this case.
But overall, we agree that this an innovative way to construct an analysis and think this is worth a future study to fully explore the applications of model selection techniques to this kind of data.

* L217 The statement doesn't seem logical to me - if such advanced imputation methods were available surely they would be better used to impute all substitutions than just model alanine then use linear regression to model the rest?

This statement was referring to some previously published models for creating computationally predicted alanine scanning data. We have now removed this sentence to improve clarity.

* L331-332 The formula used for regularising Spearman's rho makes sense, and can likely be interpreted as a regularizing prior, but we found it hard to understand its provenance and meaning from the reference. A sentence on its content (not just describing that it shrinks estimates) and a more specific reference would be useful for interested readers like ourselves.

We further explained the meaning of this formula by stating that (line 346):

> We estimated the ρ value with the empirical copula, *which is related to the standard estimator by a factor of (n-1)/(n+1).*

We also added a more specific reference for this statement (Bedő and Ong, 2016).

* L364 It says correlation results were dropped when only one residue was available whereas in figure legends it says results with less than three residues were dropped. Notwithstanding thinking three is maybe too low a cutoff, these should be consistent or clarified slightly if I've misunderstood the meaning.

Thank you for bringing this up. The three-residue-threshold is only applied to the score correlation analysis (Fig 3&4), because if there are only two residues available the correlation between AS and DMS score of alanine substitutions can only be either 1 or -1.
For model evaluation, as you mentioned, we dropped data with only one residue, because in this case, we will usually have more than 20 variants to evaluate the model performance. To emphasize this, at the end of Fig 5, 6, S4, S5, S6, we explained that:

> *Results for data pairs with only one residue are not shown*.

* It would be nice to have a bit more comment on the purpose of the final supplementary section (Replacing AS data with DMS scores of alanine substitutions) - if you have DMS alanine results it seems likely you will have the other measurements anyway.

Thank you for this comment. We added a sentence to clarify the purpose of this experiment (line 954):

> We investigated another potential approach to overcome the sparsity of AS data by replacing the AS feature with the DMS scores of alanine substitutions (DMS-Ala). *The intention of this study is to model the scenario of ideal AS data, which perfectly matches the DMS-Ala data during training*.

Manuscript with all changes highlighted
Click here to access/download;Manuscript;diff_manuscript.pdf

Integrating deep mutational scanning and low-throughput mutagenesis data to predict the impact of amino acid variants

Authors:

Yunfan Fu[1,2], Justin Bedő[1,~~3~~2,*], Anthony T. Papenfuss[1,2,~~4~~3,*,**], Alan F. Rubin[1,2,*,**]

Affiliations:

[1]The Walter and Eliza Hall Institute of Medical Research, Parkville, VIC 3052~~, Victoria~~, Australia.

[2]Department of Medical Biology, The University of Melbourne, ~~Melbourne~~Parkville, VIC 3010, Australia.

~~[3]School of Computing and Information Systems, The University of Melbourne, Melbourne, VIC 3010, Australia.~~

[~~4~~3]Peter MacCallum Cancer Centre, Melbourne, VIC 3000, Australia.

* Contributed equally

** To whom correspondence should be addressed (papenfuss@wehi.edu.au & alan.rubin@wehi.edu.au)

Abstract

Background: Evaluating the impact of amino acid variants has been a critical challenge for studying protein function and interpreting genomic data. High-throughput experimental methods like deep mutational scanning (DMS) can measure the effect of large numbers of variants in a target protein, but because DMS studies have not been performed on all proteins, researchers also model DMS data computationally to estimate variant impacts by predictors.

Results: In this study, we extended a linear regression-based predictor to explore whether incorporating data from alanine scanning (AS), a widely- used low-throughput mutagenesis method, would improve prediction results. To evaluate our model, we collected 146 AS datasets, mapping to 54 DMS datasets across 22 distinct proteins.

Conclusions: We show that improved model performance depends on the compatibility of the DMS and AS assays, and the scale of improvement is closely related to the correlation between DMS and AS results.

Keywords: deep mutational scanning, alanine scanning, machine learning, predictor

1 Introduction

Deep mutational scanning (DMS) is a functional genomics method that can experimentally measure the impact of many thousands of protein variants by combining high-throughput sequencing with a functional assay [1]. In a typical DMS, a cDNA library of genetic variants of a target gene is generated, containing all possible single amino acid substitutions. This variant library is then expressed in a functional assay system where the DMS variants can be selected based on their properties. The change in variant frequency in the pre- and post-selection populations is determined by high-throughput sequencing which is then used to calculate a multiplexed functional score that captures the variant's impact [2–4]. The versatility of DMS assays makes it possible to measure variant impact on a wide range of protein properties, including protein binding affinity [5,6], protein abundance [7–9], ~~catalytic~~enzyme activity [10,11] and cell ~~growth rate~~survival [12–14]. So far, hundreds of DMS studies covering tens of thousands of nucleotides have been published [15], and experiments targeting over a hundred additional genes are underway according to MaveRegistry [16].

Computational studies have used DMS data to build predictive models of variant impact. These predictors use supervised or semi-supervised learning models trained on experimental DMS data and various protein features to make predictions ~~[15-21]~~[17–23]. Envision is one such method that used protein structural, physicochemical, and evolutionary features to predict variant effect scores and was trained on DMS data from 8 proteins using gradient boosting ~~[15]~~[17]. Another method, DeMaSk, predicted DMS scores by combining two evolutionary features (protein positional conservation and variant homologous frequency) with a DMS substitution matrix and was trained on data from 17 proteins using a linear model ~~[17]~~[19]. Deep learning algorithms have also been applied to build protein fitness predictors ~~[16,18]~~[18,20], which are usually based only on variant sequences. These variant effect predictors can also be benchmarked using DMS experimental results and assist in the interpretation of experimental data [20,24,25].

Low-throughput mutagenesis experiments that measure tens of variants at a time have also been used extensively to study diverse protein properties, including substrate binding affinity ~~[22,23]~~[26,27], protein stability ~~[24,25]~~[28,29], and protein-specific ~~activity~~activities ~~[26,27]~~[30,31]. Alanine scanning (AS) is a widely-used low-throughput mutagenesis method ~~[28,29]~~[32,33], and AS data are available for many proteins. In this method, each targeted protein residue is substituted with alanine, and the impacts of these variants are measured by a functional assay ~~[30]~~[34]. AS experiments are typically used to identify functional hot spots or critical residues in the target protein ~~[31,32]~~[35,36] and have been used as a source of independent validation for DMS studies ~~[27,33-35]~~[31,37–39].

In this study, we explore whether a predictive model can be improved by incorporating low-throughput mutagenesis data (Fig 1). We find that AS data can increase prediction accuracy

and that the improvement is related to the similarity of the functional assays and the correlation of DMS and AS results.

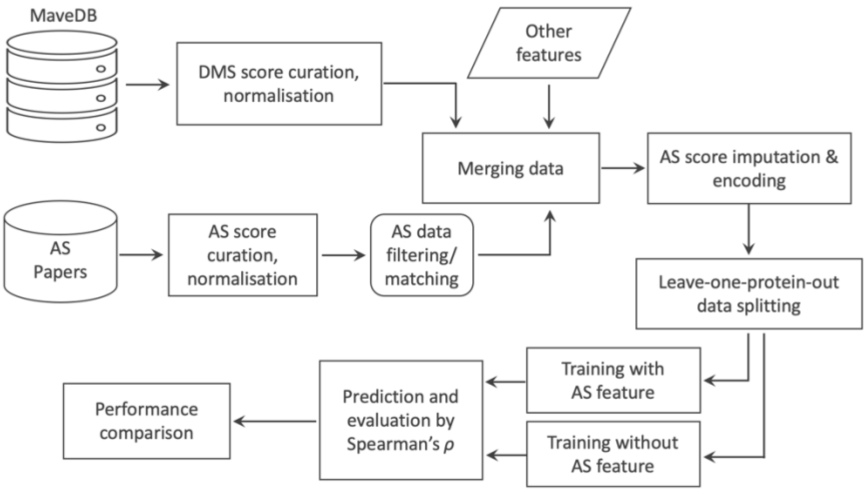

Fig 1. Workflow for model training and testing. DMS and AS datasets are collected from online resources and are normalized. DMS and AS datasets targeting the same protein are then matched, filtered and merged. Two predictors are constructed and tested: the first uses DMS data, AS data and other protein features, and the second uses only DMS data and the same other protein features.

2 Results

2.1 Overview of DMS and alanine scanning (AS) data

To build the predictive model, 130 DMS datasets were collected from MaveDB ~~[36,37]~~[40,41] (Supplementary table 1). We searched the literature and found 146 AS datasets targeting the same proteins as 54 of the DMS datasets. In total, we obtained both DMS and AS data for 22 different proteins: 17 human proteins, three yeast proteins, and two bacterial proteins. Most DMS experiments were highly complete, with a mean coverage of 95.0% of all possible single amino acid substitutions assayed in the target region, comprising 373,219 total protein variant measurements. AS data were only available on a small number of protein residues (Fig 2), and we were able to curate 1,480 alanine substitution scores from the 146 studies. Variant scores

from collected DMS and AS studies were linearly normalized to a common scale (see Methods) to make them comparable across datasets (Fig S1).

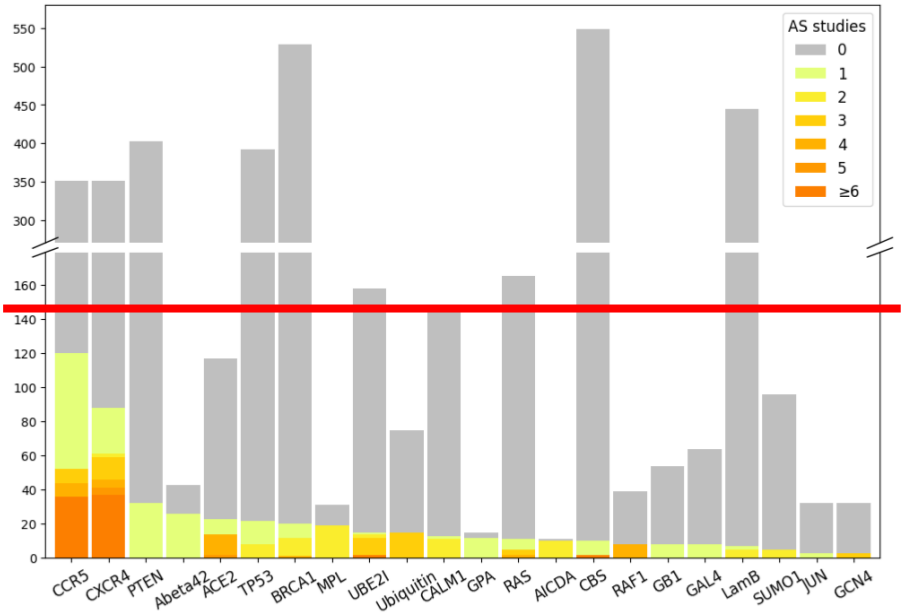

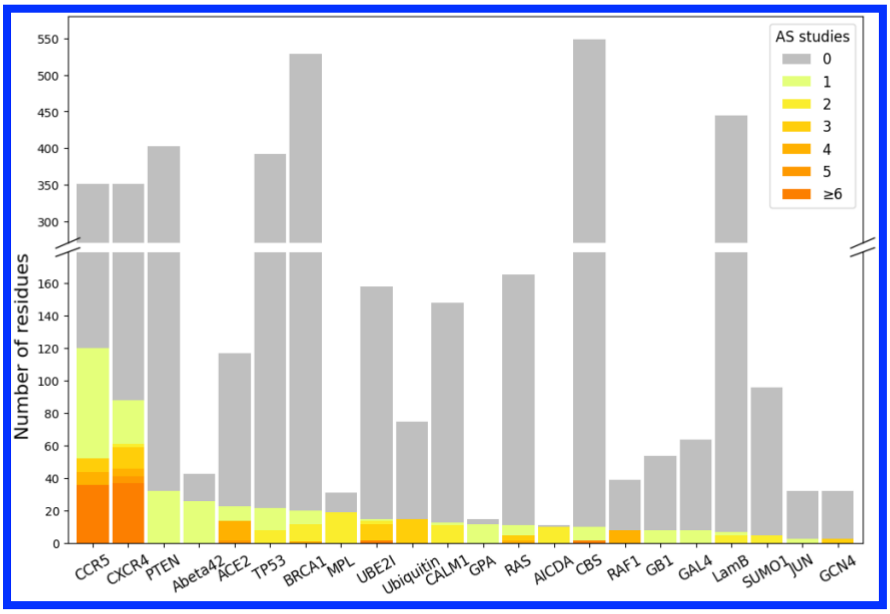

Fig 2. DMS data generally cover more protein residues than AS data. Each bar shows the number of residues assayed by DMS studies on given target proteins. Colour indicates the number of AS studies available for the DMS-tested residues.

2.2 The correlation of DMS and AS scores is related to assay compatibility

To evaluate the similarity of AS and DMS scores, we calculated Spearman's correlation (ρ) between the AS scores and DMS scores for the same alanine substitutions. Since each protein may have results from several AS and DMS experiments, we calculated ρ between each possible pair. The median ρ over DMS and AS data (DMS/AS) pairs was 0.2, indicating that the experimental scores were poorly correlated overall (Fig 3).

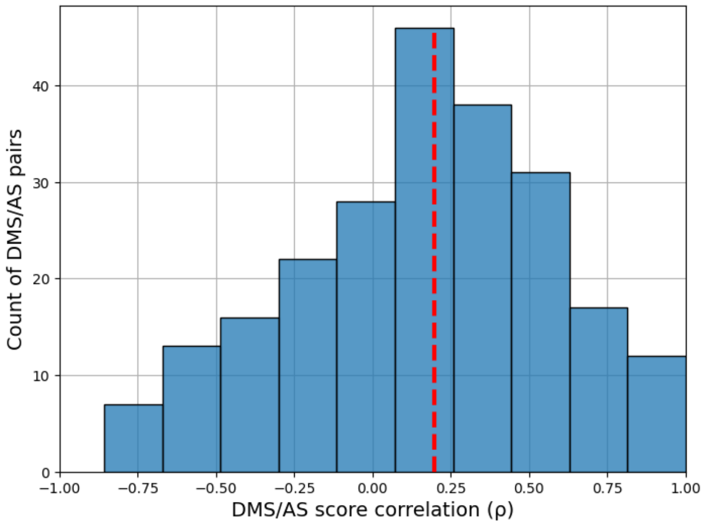

Fig 3. Correlation between DMS and AS data shows substantial variation. We calculated Spearman's ρ between alanine substitution scores in each pair of AS and DMS data. The results for pairs with less than three alanine substitutions are ~~removed~~not shown. The red dashed line shows the median ρ.

We then considered if differences between AS and DMS assay designs might contribute to this low agreement between scores. To explore this, we developed a decision tree (Fig S2) to classify whether DMS/AS pairs had low, medium, or high assay compatibility, which we defined as a similarity measurement of the functional assays performed. For example, the DMS assay measuring the binding affinity of a cell surface protein, CXCR4, to its natural ligand ~~[38]~~[42] has high compatibility with the AS experiment also measuring this ligand binding but

has low compatibility with the study on CXCR4's ability to facilitate virus infection ~~[39]~~[43]. A full assay compatibility table can be found in Supplementary Table 1 with the compatibility classifications and justification for each pair. We then compared DMS and AS score correlation for each compatibility class and found that score correlations were closely related to assay compatibility. Data from low compatibility assays had a median correlation of 0.15, rising to 0.19 for medium compatibility assays and 0.40 for high compatibility assays (Fig 4). This trend of increased correlation for high compatibility assay pairs holds across secondary structures (Table S1). This link between assay compatibility and score correlation indicates that our decision tree approach was able to capture the similarity between assay systems.

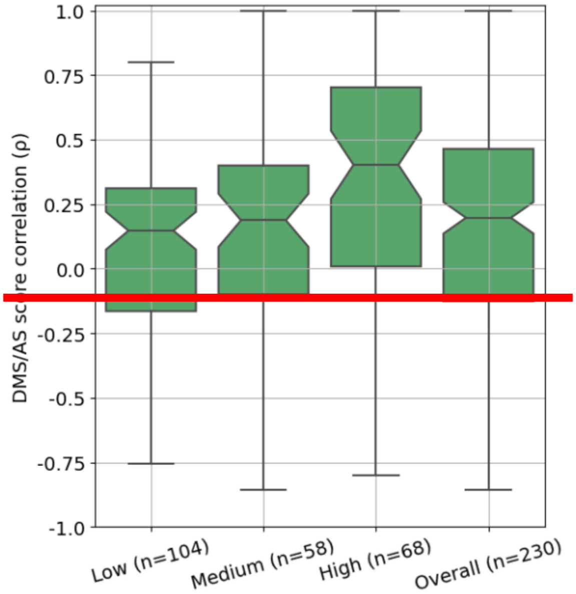

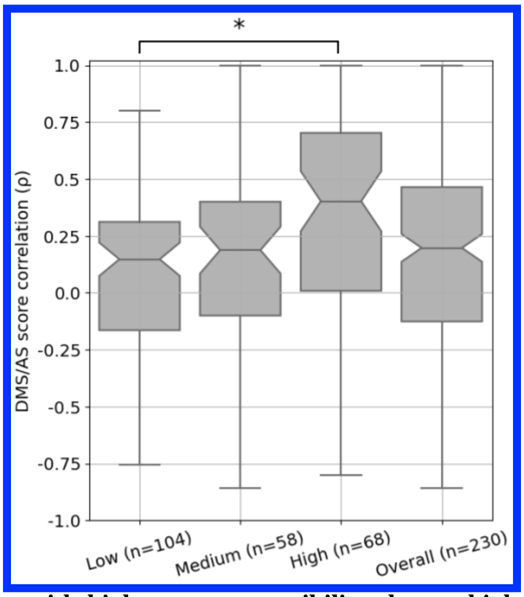

Fig 4. DMS and AS data pairs with high assay compatibility show a higher score correlation. Each box ~~represents~~shows the Spearman's ρ between DMS and AS data pairs for each level of ~~classified~~ assay compatibility or ~~the~~ overall ~~result~~. The correlation coefficients ~~are~~were calculated between alanine substitution scores in each pair of AS and DMS datasets. Results for ~~data~~ pairs with less than three alanine substitutions ~~are~~were removed. P-values calculated using Welch's test and corrected using Holm-Šidák, *: $p<0.05$; notches show 95% confidence interval around median, and whiskers show the full value range.

2.3 Compatible AS data improve DMS score prediction accuracy

To test if incorporating AS data into DMS score models would improve prediction accuracy, we decided to build a new model based on DeMaSk ~~[17]~~[19]. We chose DeMaSk because it showed better performance compared to similar methods and was straightforward to modify. The published DeMaSk model predicts DMS scores using protein positional conservation, variant homologous frequency, and substitution score matrix, and we incorporated AS data as an additional feature. Our new predictor was modelled with all 130 DMS we collected and we applied a leave-one-protein-out cross-validation approach to training and testing, avoiding information leakage for variants of the same protein target ~~[15]~~[17]. Prediction performance was evaluated using the Spearman's correlation (ρ) between the experimentally-derived DMS scores and the predicted scores for each pair of DMS and AS studies. The performance of our DMS/AS model was compared with a model trained only on DMS data, equivalent to retrained DeMaSk (Fig S3), by calculating the change of prediction ρ (see Methods).

We trained our model with either all or a subset of AS data we collected (Fig 5, Table S2~~S1~~). We first integrated all 146 AS data collected for training and evaluation but observed only a modest improvement of prediction ρ (Fig 5 left box, and Fig S4). We then retrained and evaluated our model on filtered AS data with only high compatibility assays, and observed a median increase in prediction Spearman's ρ of 0.1 compared to the results with no AS data (Fig 5 middle box, and Fig S4). However, training with both high and medium compatibility pairs reduced the performance improvement (Fig S5). These results indicate that medium and low compatibility pairs might provide inconsistent training data, degrading model performance. We also evaluated the impact of including high compatibility AS data in an alternative model based on Envison ~~[15]~~[17], and found similar results (Fig S6). To differentiate between high assay compatibility and high DMS/AS score correlation, we trained the model using the most

highly correlated AS result for each DMS dataset (see Methods). Although the upper quartile was high, the median performance change of this predictor was lower than the high assay compatibility model, suggesting that matching with the highest score correlation alone is insufficient (Fig 5 right box). However, when applying a stricter threshold, the correlation matched models still show limited improvement (Fig S7). Additionally, to ensure the models performance is not biased by pseudo-replication of multiple datasets, we averaged DMS and AS scores that were part of the same study and type of assay, and saw similar results (Fig S8).

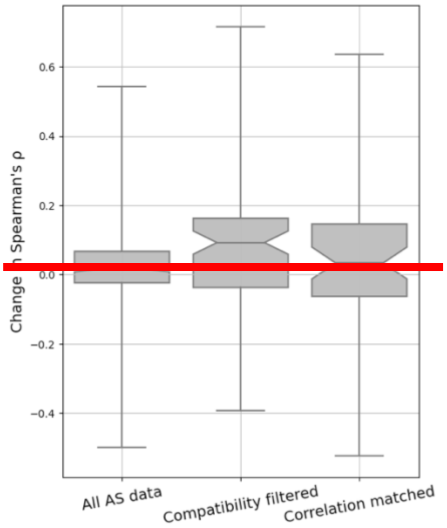

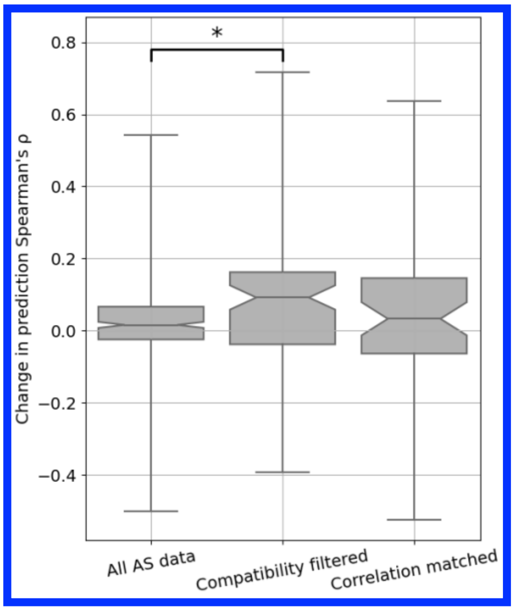

Fig 5. Performance of variant impact prediction is improved using AS data with high assay compatibility. The change ~~of~~in prediction ρ achieved by including the AS data feature for each DMS and AS data pair is shown as box plots. A higher value represents higher prediction accuracy achieved for using AS data. Different approaches to filtering/matching the data are shown on the x-axis: "All AS data" used all available data; "Compatibility filtered" used only data of high assay compatibility; "Correlation matched" used only data with the highest regularised correlation for each DMS dataset. Results for data pairs with only one residue are not shown. P-values were calculated using Welch's test and jointly corrected using Holm-Šidák (Methods), *: $p<0.05$. Notches show the 95% confidence interval around the median, and whiskers show the full value range.

Our compatibility-filtered predictor shows improved prediction accuracy for these regions compared to not only the baseline model, but other widely used predictors as well (Fig S9). To further explore the higher performance of this compatibility-filtered predictor, we examined the relationship between prediction ρ change and score correlation for each high compatibility DMS/AS pair (Fig 6). For most pairs, prediction performance was improved by using AS data, and the scale of improvement was also related to the score correlation. This relationship could also be observed for multiple DMS/AS pairs from an individual protein, such as CXCR4 and CCR5. We saw the same trend in the predictor trained with all DMS/AS pairs but noted that the performance even of highly correlated pairs was worse, likely due to the influence of low compatibility training data on the model (Fig S10~~S7~~).

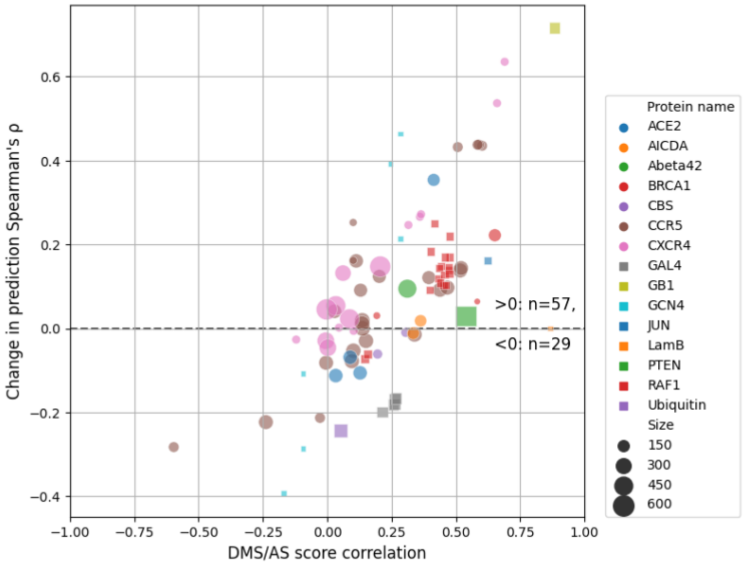

Fig 6. Prediction performance change is related to DMS and AS score correlation. Each dot represents a filtered DMS/AS data pair of high assay compatibility. The vertical axis shows the change of prediction ρ by using AS data (larger means higher performance achieved by using AS data). The horizontal axis shows the DMS/AS score correlation for *all* variants on the matched residues rather than just alanine substitutions. The colours and shapes of the dots correspond to the target protein, and size indicates the number of variants in each data pair. Results for data pairs with only one residue are not shown.

We also explored the consequences of the sparsity of AS data on our model in ~~two~~three ways: i) by training only with variants that have AS data available (Fig S11); ii) by using a boosting approach that focuses only on residues with AS data (Fig S12~~S8~~) and iii) by using complete alanine substitution information from DMS as the AS feature (Fig S13~~S9~~). ~~Both of these~~The first approach gave lower absolute prediction performance, presumably because the model was under-fitted due to the small number of variants. The last two approaches performed very similarly to the primary model constructed using high-compatibility DMS/AS data and simple mean score imputation.

To test the influence of amino acids on our predictor, we grouped the prediction results by either wild-type or variant amino acid and calculated the prediction improvement when AS data were included (Fig 7). We found that 14 of 19 wild-type amino acids performed better with the addition of AS data, with cysteine showing the largest improvement and performing worst in the model lacking AS data. 18 of 20 variant amino acids benefited from the inclusion of AS data, with marginal performance decrease on lysine and aspartic acid ($|\Delta\rho|<0.01$) (Fig 7). We also noticed that variants to alanine are not most improved, however we observed an overall trend showing higher improvement for amino acids that are physiochemically similar to alanine (Fig S15).

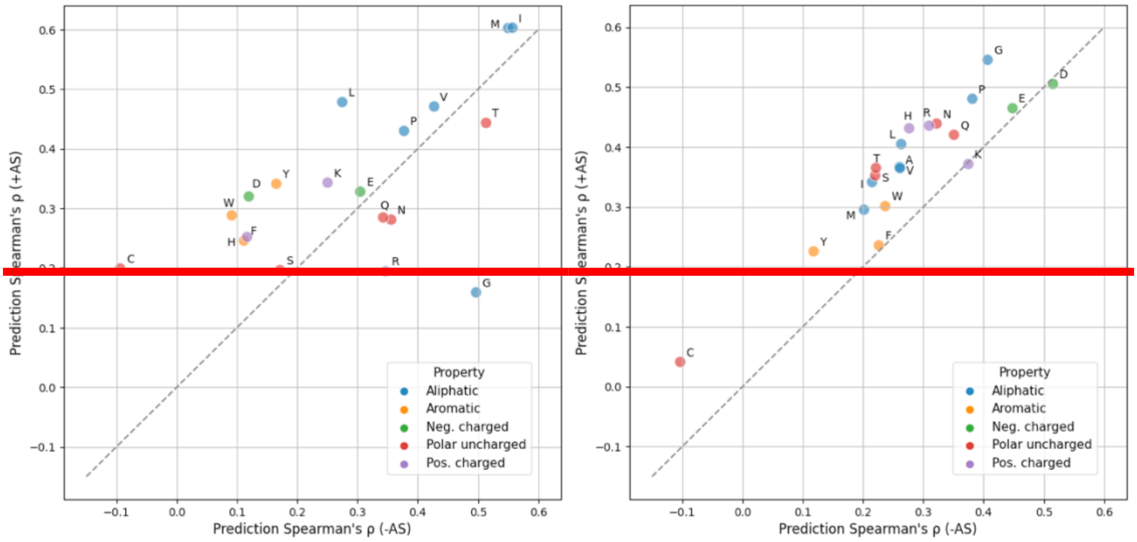

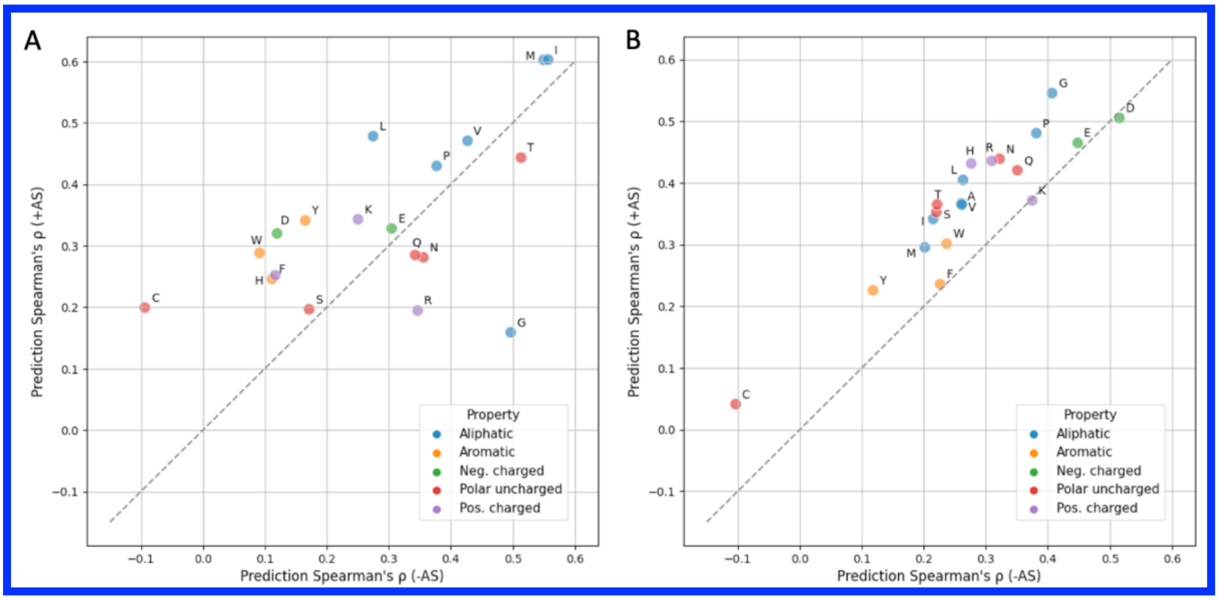

Fig 7. Model ~~perfomance~~performance is generally improved for each wild-type and variant amino acid. Prediction Spearman's ρ when using (y-axis) or not using (x-axis) AS data on each wild-type (~~left~~**A**) or variant (~~right~~**B**) amino acid is shown in the scatter plots. The results are coloured according to the property of each amino acid type. Alanine (A) result is not applicable in the first figure since alanine scanning data are always missing when the wildtype is alanine itself. Absolute count for each amino acid can be found in Fig S14~~S10~~. (Neg.: negatively, Pos.: positively)

3 Discussion

In this study, we integrated alanine scanning (AS) data into deep mutational scanning (DMS) score prediction, leading to modest improvements in the accuracy of variant score prediction. We also explored the impact of the diversity of protein properties measured by DMS and AS. Filtering DMS and AS data based on our manual classification of assay type compatibility led to improved prediction performance.

A potential shortcoming of our current approach is that AS data were available for only a small proportion of the DMS data. Although most recent DMS studies can analyze variants of the whole protein, most AS experiments only cover a handful of residues in the target protein, leaving missing AS scores for the vast majority of residues. We explored this here and found that alternative methods for addressing the sparsity of AS data did not improve or degrade performance, but we anticipate further improved prediction accuracy if the low completeness and unevenness of AS data are appropriately handled before modelling~~, such as by advanced imputation methods [48,49]~~.

In this study, we identified the importance of DMS/AS assay compatibility as a crucial factor for improving prediction accuracy. An issue with using this concept is that it further shrinks already sparse data. It also fails to take advantage of the fact that even for low compatible assays some fundamental information like protein ~~stability~~abundance can still be mutually captured. Instead of hard filtering, proper implementation of this underlying information may facilitate variant impact prediction in the future. Nonetheless, filtering on assay compatibility still leads to performance improvement. We also briefly explored whether the consistency of DMS and AS scores can be considered more directly by matching the best correlated AS data for each DMS dataset. Consistency is partially driven by assay compatibility but also reflects

other features of the data, such as bias and noise. ~~While we picked the most correlated pair for each DMS, we did not threshold the correlation, potentially including data pairs that were poor matches.~~

The concepts of compatibility and data quality are also relevant to training any DMS-based predictors. DMS assays have been developed to measure variant impacts to distinct protein properties, and a variant can behave similarly to wildtype when measured by one assay yet show altered protein properties in other assay results, which are frequently found in regions with specific biochemical functions ~~[50-55]~~[25,52–56]. With more experimental assays to be applied, the diverse measurements may impede the progress of future DMS-based predictors unless this assay effect is properly addressed, for example, by building assay specific predictors. Measurement error is another source of DMS data heterogeneity that potentially affects the model performance. In our current study, DMS scores of protein variants are weighted equally while training. Adjustable weighting can be applied in future studies to adapt the distinct experimental error between individual variants and datasets, reducing the influence of low-confident data.

In summary, we conclude that the careful inclusion of low-throughput mutagenesis data improves the prediction of DMS scores, and the approaches described here can potentially be applied to other prediction methods.

4 Availability of supporting source code and requirements

Project name: DMS_with_Alanine_scan

Project home page: https://github.com/PapenfussLab/DMS_with_Alanine_scan

Operating system: Platform independent

Programming language: Python

Other requirements: Python 3.10~~.6~~ or higher

Licence: MIT Licence

5 List of abbreviations

DMS: deep mutational scanning

AS: alanine scanning

6 Supporting information

Supplementary Table 1: All candidate DMS and alanine scanning data with detailed dataset information.

Supplementary Table 2: Normalized DMS dataset with protein property features.

Supplementary Table 3: Normalized alanine scanning dataset.

7 Author contributions

YF developed the software and wrote the initial draft of the manuscript. AFR conceived the study. JB, AFR, and ATP oversaw the project. All authors reviewed, contributed to, and approved the manuscript.

8 Funding

YF is supported by Melbourne Research Scholarship. ATP was supported by an Australian National Health and Medical Research Council (NHMRC) Senior Research Fellowship (1116955). JB, AFR and ATP were supported by the Lorenzo and Pamela Galli Medical Research Trust. JB and ATP were supported by the Stafford Fox Medical Research

Foundation. AFR was supported by the National Human Genome Research Institute of the NIH under award numbers RM1HG010461 and UM1HG011969. The research benefitted from support from the Victorian State Government Operational Infrastructure Support and Australian Government NHMRC Independent Research Institute Infrastructure Support.

9 Methods

9.1 DMS data collection

DMS data were downloaded from MaveDB ~~[36,37]~~[40,41] which were then filtered and curated. DMS experiments targeting antibody and virus proteins were removed because of their potentially unique functionality. We retrieved the UniProt accession ID of target proteins by searching the protein names or sequences in UniProt ~~[56]~~[57], and proteins lacking available UniProt ID were also excluded. Datasets that are computationally processed or their wildtype-like and nonsense-like scores (see Normalization) cannot be identified were also filtered out (Supplementary Table 1). All missense variants with only a single amino acid substitution were curated from the DMS studies for our analysis. A total of 130 DMS experiments from 53 studies ~~[5,6,9–14,27,33–35,38,57–94]~~[5,6,9–14,24,31,37–39,42,58–94] were collected for our analysis.

9.2 Collection of AS data and other features

The following process was used to search for candidate AS studies. Papers were identified by searching on PubMed and Google Scholar for the “alanine scan” or “alanine scanning” together with the name of candidate proteins. While searching in Google Scholar, we included the protein’s UniProt ID rather than molecule name as the search term to reduce false positives. Appropriate AS data were collected from the search results. Western blot results were transformed to values by ImageJ if it was the only experimental data available in the study. A

total 146 AS experiments were collected from 45 distinct studies ~~[22–24,26,27,39–42,44,45,84,95–127]~~[26–28,30,31,43–46,48,49,84,95–127].

Protein features of Shannon entropy and the logarithm of variant amino acid frequency were downloaded from the DeMaSk online toolkit ~~[17]~~[19]. The substitution score matrix feature was calculated from the mean of training DMS scores for each of the 380 possible amino acid substitutions before each iteration of cross-validation.

9.3 Normalization

DMS and AS datasets were normalized to a common scale using the following approach adapted from previous studies ~~[15,43]~~[17,47]. Let *D* denotes a protein study measuring scores s_i^D for a single variant *i*, s_{wt}^D denotes the scores for wildtype and s_{non}^D represents the score for nonsense-like variants. The normalized scores $s_i'^D$ are given by:

$$s_i'^D := \frac{s_i^D - s_{wt}^D}{s_{wt}^D - s_{non}^D} + 1$$

Wild-type scores were directly identified from the paper or the median score of synonymous variants. For DMS data, since not all DMS studies report score of nonsense variants, we defined the nonsense-like scores as the median DMS scores for the 1% missense variants with the strongest loss of function for each dataset. For AS data, nonsense-like scores were either defined according to the paper or using the extreme values (Supplementary Table 1).

9.4 AS data filtering and matching

AS data subsets were filtered/matched according to either assay compatibility or score correlation. For assay compatibility filtering, we first categorized each DMS ~~and~~or AS assay by the protein property or function using the following assay types: binding affinity, enzyme activity, protein abundance, cell survival, pathogen infection, drug response, ability to perform

a novel function, or other protein-specific activities (e.g., transcription activity for transcription factors) (Supplementary Table 1). The DMS/AS assay pairs were ~~first~~then classified into three levels of compatibility based on these categories (Fig S2). For each DMS dataset, we first tried to use only AS data with high assay compatibility for further modelling, removing AS data of medium and low assay compatibility. We then also tried to model with AS data of both high and medium assay compatibility.

For score correlation matching, Spearman's correlation (ρ) is calculated between alanine substitution scores in each pair of AS and DMS data. To avoid influence from the size of AS datasets, we ~~regularised~~estimated the ρ value ~~by~~with the empirical copula, which is related to the standard estimator by a factor of (n-1)/(n+1) [128,129]:

$$\rho_r := \rho \times \frac{n-1}{n+1}$$

where ρ_r is the regularised correlation coefficient, and n is the number of alanine substitutions used for correlation calculation. For each DMS dataset, AS result with the highest ρ_r was picked for modelling.

9.5 AS data pre-processing

AS data were pre-processed prior to modelling. For variants without available (filtered/matched) AS data, their AS scores were imputed with the mean value of all available AS scores across all studies. Then the AS data were encoded by the wild-type and variant amino acid type with one-hot-encoding. For each variant, the AS feature is expanded with two one-hot vectors. Each of the vectors has 19 zeros and one non-zero value which was the AS score, with the location of the non-zero value indicating the wild-type or variant amino acid type.

9.6 Training and evaluation of DMS score predictor

To build the predictors, we performed linear regression using the function `sklearn.linear_model.LinearRegression` from scikit-learn ~~[129]~~[130]. Training and validation data were separated with leave-one-protein-out cross-validation. In this process, data from one protein were withheld for subsequent validation, and the rest were used for training. This process was iterated over all proteins in the data. Variants were inversely weighted during the training process by the number of measurements available, thus compensating for some regions having greater coverage with DMS and AS assays. Predictors were trained on protein features, DMS data and (optionally) AS data using four different filtering or matching strategies: i) all DMS/AS data, ii) compatibility-filtered DMS/AS data, iii) correlation-matched DMS/AS data, and iv) a control, constructed using DMS data only.

In the evaluation process, let V be protein variants assayed by both DMS study D and AS study A. Variant scores are predicted by the previously mentioned predictors either using AS data ($\hat{s}_V^A$) or not ($\hat{s}_V$). Spearman's correlation (ρ) was calculated between the DMS scores s_V^D and each set of predicted scores. The difference of ρ was used to evaluate the performance change ($\Delta\rho_V$).

$$\rho_V^A = \text{Spearman's correlation}(\hat{s}_V^A, s_V^D)$$

$$\rho_V = \text{Spearman's correlation}(\hat{s}_V, s_V^D)$$

$$\Delta\rho_V = \rho_V^A - \rho_V$$

To evaluate, we iterated over variants from each pair of DMS/AS studies. Results were dropped for variants V with only one protein residue available during analysis and visualization. Model performance was compared using the following statistical tests. Results in Fig 5 & Fig S5 were tested with Welch's test, and results in Fig S4 & Fig S6 were tested with paired t-tests. The p-values were jointly corrected using the Holm–Šidák method. The 95% confidence interval of median values are calculated by Gaussian-based asymptotic approximation [131].

9.7 Prediction with other variant effect predictors

For PROVEAN [132] and SIFT [133], prediction results on target variants were directly downloaded from the pre-calculated database for PROVEAN. For PolyPhen-2 [134] and GEMME [135], variant scores were computed through their online toolkits, using the default settings. ESM-1v [136] was set up locally and run according to its examples and documentations. EVE [137] results were collected from their pre-calculated database and a benchmarking study [138].

10 References

1. Fowler DM, Fields S. Deep mutational scanning: a new style of protein science. Nature Methods. 2014; doi: 10.1038/nmeth.3027.

2. Findlay GM. Linking genome variants to disease: scalable approaches to test the functional impact of human mutations. Human Molecular Genetics. 2021; doi: 10.1093/hmg/ddab219.

3. Geck RC, Boyle G, Amorosi CJ, Fowler DM, Dunham MJ. Measuring Pharmacogene Variant Function at Scale Using Multiplexed Assays. Annual Review of Pharmacology and Toxicology. 2022; doi: 10.1146/annurev-pharmtox-032221-085807.

4. Weile J, Roth FP. Multiplexed assays of variant effects contribute to a growing genotype–phenotype atlas. Hum Genet. 2018; doi: 10.1007/s00439-018-1916-x.

5. Diss G, Lehner B. The genetic landscape of a physical interaction. eLife. 2018; doi: 10.7554/eLife.32472.

6. Fowler DM, Araya CL, Fleishman SJ, Kellogg EH, Stephany JJ, Baker D, et al.. High-resolution mapping of protein sequence-function relationships. Nature Methods. 2010; doi: 10.1038/nmeth.1492.

7. Amorosi CJ, Chiasson MA, McDonald MG, Wong LH, Sitko KA, Boyle G, et al.. Massively parallel characterization of CYP2C9 variant enzyme activity and abundance. The American Journal of Human Genetics. 2021; doi: 10.1016/j.ajhg.2021.07.001.

8. Faure AJ, Domingo J, Schmiedel JM, Hidalgo-Carcedo C, Diss G, Lehner B. Mapping the energetic and allosteric landscapes of protein binding domains. Nature. 2022; doi: 10.1038/s41586-022-04586-4.

9. Matreyek KA, Starita LM, Stephany JJ, Martin B, Chiasson MA, Gray VE, et al.. Multiplex assessment of protein variant abundance by massively parallel sequencing. Nature Genetics. 2018; doi: 10.1038/s41588-018-0122-z.

10. Mighell TL, Evans-Dutson S, O'Roak BJ. A Saturation Mutagenesis Approach to Understanding PTEN Lipid Phosphatase Activity and Genotype-Phenotype Relationships. The Amer-ican Journal of Human Genetics. 2018; doi: 10.1016/j.ajhg.2018.03.018.

11. Stiffler MA, Hekstra DR, Ranganathan R. Evolvability as a Function of Purifying Selection in TEM-1 β-Lactamase. Cell. 2015; doi: 10.1016/j.cell.2015.01.035.

12. Ahler E, Register AC, Chakraborty S, Fang L, Dieter EM, Sitko KA, et al.. A Combined Approach Reveals a Regulatory Mechanism Coupling Src's Kinase Activity, Localization, and Phosphotransferase-Independent Functions. Molecular Cell. 2019; doi: 10.1016/j.molcel.2019.02.003.

13. Giacomelli AO, Yang X, Lintner RE, McFarland JM, Duby M, Kim J, et al.. Mutational processes shape the landscape of TP53 mutations in human cancer. Nature Genetics. Nature Publishing Group; 2018; doi: 10.1038/s41588-018-0204-y.

14. Roscoe BP, Thayer KM, Zeldovich KB, Fushman D, Bolon DNA. Analyses of the Effects of All Ubiquitin Point Mutants on Yeast Growth Rate. Journal of Molecular Biology. 2013; doi: 10.1016/j.jmb.2013.01.032.

15. Tabet D, Parikh V, Mali P, Roth FP, Claussnitzer M. Scalable Functional Assays for the Interpretation of Human Genetic Variation. Annu Rev Genet. 2022; doi: 10.1146/annurev-genet-072920-032107.

16. Kuang D, Weile J, Kishore N, Nguyen M, Rubin AF, Fields S, et al.. MaveRegistry: a collaboration platform for multiplexed assays of variant effect. Lu Z, editor. Bioinformatics. 2021; doi: 10.1093/bioinformatics/btab215.

~~15.~~17. Gray VE, Hause RJ, Luebeck J, Shendure J, Fowler DM. Quantitative Missense Variant Effect Prediction Using Large-Scale Mutagenesis Data. Cell Systems. 2018; doi: 10.1016/j.cels.2017.11.003.

~~16.~~18. Alley EC, Khimulya G, Biswas S, AlQuraishi M, Church GM. Unified rational protein en-gineering with sequence-based deep representation learning. Nat Methods. 2019; doi: 10.1038/s41592-019-0598-1.

~~17~~19. Munro D, Singh M. DeMaSk: a deep mutational scanning substitution matrix and its use for variant impact prediction. Xu J, editor. Bioinformatics. 2020; doi: 10.1093/bioinformatics/btaa1030.

~~18~~20. Biswas S, Khimulya G, Alley EC, Esvelt KM, Church GM. Low- N protein engineering with data-efficient deep learning. Nature Methods. Nature Publishing Group; 2021; doi: 10.1038/s41592-021-01100-y.

~~19~~21. Høie MH, Cagiada M, Beck Frederiksen AH, Stein A, Lindorff-Larsen K. Predicting and interpreting large-scale mutagenesis data using analyses of protein stability and conserva-tion. Cell Reports. 2022; doi: 10.1016/j.celrep.2021.110207.

~~20~~22. Wu Y, Li R, Sun S, Weile J, Roth FP. Improved pathogenicity prediction for rare human missense variants. The American Journal of Human Genetics. 2021; doi: 10.1016/j.ajhg.2021.08.012.

~~21~~23. Hsu C, Nisonoff H, Fannjiang C, Listgarten J. Learning protein fitness models from evolutionary and assay-labeled data. Nat Biotechnol. 2022; doi: 10.1038/s41587-021-01146-5.

24. Findlay GM, Daza RM, Martin B, Zhang MD, Leith AP, Gasperini M, et al.. Accurate classification of BRCA1 variants with saturation genome editing. Nature. 2018; doi: 10.1038/s41586-018-0461-z.

25. Cagiada M, Bottaro S, Lindemose S, Schenstrøm SM, Stein A, Hartmann-Petersen R, et al.. Discovering functionally important sites in proteins. bioRxiv;

~~22~~26. Block C, Janknecht R, Herrmann C, Nassar N, Wittinghofer A. Quantitative structure-activity analysis correlating Ras/Raf interaction in vitro to Raf activation in vivo. Nature Structural Biology. Nature Publishing Group; 1996; doi: 10.1038/nsb0396-244.

~~23~~27. Sloan DJ, Hellinga HW. Dissection of the protein G B1 domain binding site for human IgG Fc fragment. Protein Science. 1999; doi: 10.1110/ps.8.8.1643.

~~24~~28. Fleming KG, Engelman DM. Specificity in transmembrane helix–helix interactions can define a hierarchy of stability for sequence variants. PNAS. National Academy of Sciences; 2001; doi: 10.1073/pnas.251367498.

~~25~~29. Shibata Y, White JF, Serrano-Vega MJ, Magnani F, Aloia AL, Grisshammer R, et al.. Thermostabilization of the Neurotensin Receptor NTS1. Journal of Molecular Biology. 2009; doi: 10.1016/j.jmb.2009.04.068.

~~26~~30. Brzovic PS, Heikaus CC, Kisselev L, Vernon R, Herbig E, Pacheco D, et al.. The Acidic Transcription Activator Gcn4 Binds the Mediator Subunit Gal11/Med15 Using a Simple Protein Interface Forming a Fuzzy Complex. Molecular Cell. 2011; doi: 10.1016/j.molcel.2011.11.008.

~~27~~31. Gajula KS, Huwe PJ, Mo CY, Crawford DJ, Stivers JT, Radhakrishnan R, et al.. High-throughput mutagenesis reveals functional determinants for DNA targeting by activation-induced deaminase. Nucleic Acids Research. 2014; doi: 10.1093/nar/gku689.

~~28~~32. Kortemme T, Kim DE, Baker D. Computational Alanine Scanning of Protein-Protein Interfaces. Science's STKE. American Association for the Advancement of Science; 2004; doi: 10.1126/stke.2192004pl2.

~~29~~33. Morrison KL, Weiss GA. Combinatorial alanine-scanning. Current Opinion in Chemical Biology. 2001; doi: 10.1016/S1367-5931(00)00206-4.

~~30~~34. Cunningham BC, Wells JA. High-resolution epitope mapping of hGH-receptor interactions by alanine-scanning mutagenesis. Science. American Association for the Advancement of Science; 1989; doi: 10.1126/science.2471267.

~~31~~35. DeLano WL. Unraveling hot spots in binding interfaces: progress and challenges. Current Opinion in Structural Biology. 2002; doi: 10.1016/S0959-440X(02)00283-X.

~~32~~36. Eustache S, Leprince J, Tufféry P. Progress with peptide scanning to study structure-activity relationships: the implications for drug discovery. Expert Opinion on Drug Discovery. 2016; doi: 10.1080/17460441.2016.1201058.

~~33~~37. Olson CA, Wu NC, Sun R. A Comprehensive Biophysical Description of Pairwise Epistasis throughout an Entire Protein Domain. Current Biology. 2014; doi: 10.1016/j.cub.2014.09.072.

~~34~~38. Staller MV, Holehouse AS, Swain-Lenz D, Das RK, Pappu RV, Cohen BA. A High-Throughput Mutational Scan of an Intrinsically Disordered Acidic Transcriptional Activation Domain. Cell Systems. 2018; doi: 10.1016/j.cels.2018.01.015.

~~35~~39. Gray VE, Sitko K, Kameni FZN, Williamson M, Stephany JJ, Hasle N, et al.. Elucidating the Molecular Determinants of Aβ Aggregation with Deep Mutational Scanning. G3 (Bethesda). 2019; doi: 10.1534/g3.119.400535.

~~36~~40. Esposito D, Weile J, Shendure J, Starita LM, Papenfuss AT, Roth FP, et al.. MaveDB: an open-source platform to distribute and interpret data from multiplexed assays of variant effect. Genome Biol. 2019; doi: 10.1186/s13059-019-1845-6.

~~37~~41. Rubin AF, Min JK, Rollins NJ, Da EY, Esposito D, Harrington M, et al.. MaveDB v2: a curated community database with over three million variant effects from multiplexed functional assays. bioRxiv;

~~38~~42. Heredia JD, Park J, Brubaker RJ, Szymanski SK, Gill KS, Procko E. Mapping Interaction Sites on Human Chemokine Receptors by Deep Mutational Scanning. The Journal of Immunology. American Association of Immunologists; 2018; doi: 10.4049/jimmunol.1800343.

~~39~~43. Tian S, Choi W-T, Liu D, Pesavento J, Wang Y, An J, et al.. Distinct Functional Sites for Human Immunodeficiency Virus Type 1 and Stromal Cell-Derived Factor 1α on CXCR4 Transmembrane Helical Domains. JVI. 2005; doi: 10.1128/JVI.79.20.12667-12673.2005.

~~40~~44. Chabot DJ, Zhang P-F, Quinnan GV, Broder CC. Mutagenesis of CXCR4 Identifies Important Domains for Human Immunodeficiency Virus Type 1 X4 Isolate Envelope-Mediated Membrane Fusion and Virus Entry and Reveals Cryptic Coreceptor Activity for R5 Isolates. J Virol. 1999; doi: 10.1128/JVI.73.8.6598-6609.1999.

~~41~~45. Han DP, Penn-Nicholson A, Cho MW. Identification of critical determinants on ACE2 for SARS-CoV entry and development of a potent entry inhibitor. Virology. 2006; doi: 10.1016/j.virol.2006.01.029.

~~42~~46. Fujita–Yoshigaki J, Shirouzu M, Ito Y, Hattori S, Furuyama S, Nishimura S, et al.. A Constitutive Effector Region on the C-terminal Side of Switch I of the Ras Protein. J Biol Chem. American Society for Biochemistry and Molecular Biology; 1995; doi: 10.1074/jbc.270.9.4661.

~~43~~47. Gray VE, Hause RJ, Fowler DM. Analysis of Large-Scale Mutagenesis Data To Assess the Impact of Single Amino Acid Substitutions. Genetics. 2017; doi: 10.1534/genetics.117.300064.

~~44~~48. Hidalgo P, Ansari AZ, Schmidt P, Hare B, Simkovich N, Farrell S, et al.. Recruitment of the transcriptional machinery through GAL11P: structure and interactions of the GAL4 dimeri-zation domain. Genes Dev. 2001; doi: 10.1101/gad.873901.

~~45~~49. Rodríguez-Escudero I, Oliver MD, Andrés-Pons A, Molina M, Cid VJ, Pulido R. A comprehensive functional analysis of PTEN mutations: implications in tumor- and autism-related syndromes. Human Molecular Genetics. 2011; doi: 10.1093/hmg/ddr337.

~~46~~50. Schröter C, Günther R, Rhiel L, Becker S, Toleikis L, Doerner A, et al.. A generic ap-proach to engineer antibody pH-switches using combinatorial histidine scanning libraries and yeast display. mAbs. 2015; doi: 10.4161/19420862.2014.985993.

~~47~~51. Starace DM, Bezanilla F. Histidine Scanning Mutagenesis of Basic Residues of the S4 Segment of the Shaker K+ Channel. J Gen Physiol. 117:469–902001;

~~48. Stekhoven DJ, Buhlmann P. MissForest--non-parametric missing value imputation for mixed-type data. Bioinformatics. 2012; doi: 10.1093/bioinformatics/btr597.~~

~~49. Wu Y, Weile J, Cote AG, Sun S, Knapp J, Verby M, et al.. A web application and service for imputing and visualizing missense variant effect maps. Schwartz R, editor. Bioinformatics. 2019; doi: 10.1093/bioinformatics/btz012.~~

~~50~~52. Cagiada M, Johansson KE, Valanciute A, Nielsen SV, Hartmann-Petersen R, Yang JJ, et al.. Understanding the Origins of Loss of Protein Function by Analyzing the Effects of Thousands of Variants on Activity and Abundance. Ozkan B, editor. Molecular Biology and Evolution. 2021; doi: 10.1093/molbev/msab095.

~~51. Cagiada M, Bottaro S, Lindemose S, Schenstrøm SM, Stein A, Hartmann-Petersen R, et al.. Discovering functionally important sites in proteins. bioRxiv;~~

~~52~~53. Jepsen MM, Fowler DM, Hartmann-Petersen R, Stein A, Lindorff-Larsen K. Chapter 5 - Classifying disease-associated variants using measures of protein activity and stability. In: Pey AL, editor. Protein Homeostasis Diseases. Academic Press;

~~53~~54. Matreyek KA, Stephany JJ, Ahler E, Fowler DM. Integrating thousands of PTEN variant activity and abundance measurements reveals variant subgroups and new dominant negatives in cancers. Genome Med. 2021; doi: 10.1186/s13073-021-00984-x.

~~54~~55. Mighell TL, Thacker S, Fombonne E, Eng C, O'Roak BJ. An Integrated Deep-Mutational-Scanning Approach Provides Clinical Insights on PTEN Genotype-Phenotype Relationships. The American Journal of Human Genetics. 2020; doi: 10.1016/j.ajhg.2020.04.014.

~~55~~56. Nielsen SV, Hartmann-Petersen R, Stein A, Lindorff-Larsen K. Multiplexed assays reveal effects of missense variants in MSH2 and cancer predisposition. PLOS Genetics. Public Library of Science; 2021; doi: 10.1371/journal.pgen.1009496.

~~56~~57. The UniProt Consortium, Bateman A, Martin M-J, Orchard S, Magrane M, Agivetova R, et al.. UniProt: the universal protein knowledgebase in 2021. Nucleic Acids Research. 2021; doi: 10.1093/nar/gkaa1100.

~~57~~58. Andrews B, Fields S. Distinct patterns of mutational sensitivity for λ resistance and maltodextrin transport in Escherichia coli LamB. Microb Genom. 2020; doi: 10.1099/mgen.0.000364.

~~58~~59. Bandaru P, Shah NH, Bhattacharyya M, Barton JP, Kondo Y, Cofsky JC, et al.. Deconstruction of the Ras switching cycle through saturation mutagenesis. eLife. 2017; doi: 10.7554/eLife.27810.

~~59~~60. Bolognesi B, Faure AJ, Seuma M, Schmiedel JM, Tartaglia GG, Lehner B. The mutational landscape of a prion-like domain. Nat Commun. 2019; doi: 10.1038/s41467-019-12101-z.

~~60~~61. Bridgford JL, Lee SM, Lee CMM, Guglielmelli P, Rumi E, Pietra D, et al.. Novel drivers and modifiers of MPL-dependent oncogenic transformation identified by deep mutational scanning. Blood. American Society of Hematology; 2020; doi: 10.1182/blood.2019002561.

~~61~~62. Chan KK, Dorosky D, Sharma P, Abbasi SA, Dye JM, Kranz DM, et al.. Engineering human ACE2 to optimize binding to the spike protein of SARS coronavirus 2. Science. American Association for the Advancement of Science; 2020; doi: 10.1126/science.abc0870.

~~62~~63. Chiasson MA, Rollins NJ, Stephany JJ, Sitko KA, Matreyek KA, Verby M, et al.. Multiplexed measurement of variant abundance and activity reveals VKOR topology, active site and human variant impact. Elife. 2020; doi: 10.7554/eLife.58026.

~~63~~64. Elazar A, Weinstein J, Biran I, Fridman Y, Bibi E, Fleishman SJ. Mutational scanning reveals the determinants of protein insertion and association energetics in the plasma membrane. Shan Y, editor. eLife. eLife Sciences Publications, Ltd; 2016; doi: 10.7554/eLife.12125.

~~64. Findlay GM, Daza RM, Martin B, Zhang MD, Leith AP, Gasperini M, et al.. Accurate classification of BRCA1 variants with saturation genome editing. Nature. 2018; doi: 10.1038/s41586-018-0461-z.~~

65. Firnberg E, Labonte JW, Gray JJ, Ostermeier M. A Comprehensive, High-Resolution Map of a Gene’s Fitness Landscape. Mol Biol Evol. 2014; doi: 10.1093/molbev/msu081.

66. Hietpas RT, Jensen JD, Bolon DNA. Experimental illumination of a fitness landscape. Proceedings of the National Academy of Sciences. 2011; doi: 10.1073/pnas.1016024108.

67. Hietpas RT, Bank C, Jensen JD, Bolon DNA. Shifting fitness landscapes in response to altered environments. Evolution. 2013; doi: 10.1111/evo.12207.

68. Jiang L, Mishra P, Hietpas RT, Zeldovich KB, Bolon DNA. Latent Effects of Hsp90 Mutants Revealed at Reduced Expression Levels. PLOS Genetics. Public Library of Science; 2013; doi: 10.1371/journal.pgen.1003600.

69. Jiang RJ. Exhaustive Mapping of Missense Variation in Coronary Heart Disease-related Genes [Thesis]. University of Toronto;

70. Keskin A, Akdoğan E, Dunn CD. Evidence for Amino Acid Snorkeling from a High-Resolution, In Vivo Analysis of Fis1 Tail-Anchor Insertion at the Mitochondrial Outer Membrane. Genetics. 2017; doi: 10.1534/genetics.116.196428.

71. Kitzman JO, Starita LM, Lo RS, Fields S, Shendure J. Massively parallel single-amino-acid mutagenesis. Nat Methods. 2015; doi: 10.1038/nmeth.3223.

72. Kotler E, Shani O, Goldfeld G, Lotan-Pompan M, Tarcic O, Gershoni A, et al.. A Systematic p53 Mutation Library Links Differential Functional Impact to Cancer Mutation Pattern and Evolutionary Conservation. Molecular Cell. Elsevier; 2018; doi: 10.1016/j.molcel.2018.06.012.

73. Kowalsky CA, Whitehead TA. Determination of binding affinity upon mutation for type I dockerin–cohesin complexes from Clostridium thermocellum and Clostridium cellulolyticum using deep sequencing. Proteins: Structure, Function, and Bioinformatics. 2016; doi: 10.1002/prot.25175.

74. McLaughlin Jr RN, Poelwijk FJ, Raman A, Gosal WS, Ranganathan R. The spatial architecture of protein function and adaptation. Nature. 2012; doi: 10.1038/nature11500.

75. Melamed D, Young DL, Gamble CE, Miller CR, Fields S. Deep mutational scanning of an RRM domain of the Saccharomyces cerevisiae poly(A)-binding protein. RNA. 2013; doi: 10.1261/rna.040709.113.

76. Mishra P, Flynn JM, Starr TN, Bolon DNA. Systematic Mutant Analyses Elucidate General and Client-Specific Aspects of Hsp90 Function. Cell Reports. 2016; doi: 10.1016/j.celrep.2016.03.046.

77. Nedrud D, Coyote-Maestas W, Schmidt D. A large-scale survey of pairwise epistasis reveals a mechanism for evolutionary expansion and specialization of PDZ domains. Proteins: Structure, Function, and Bioinformatics. 2021; doi: 10.1002/prot.26067.

78. Newberry RW, Arhar T, Costello J, Hartoularos GC, Maxwell AM, Naing ZZC, et al.. Robust Sequence Determinants of α-Synuclein Toxicity in Yeast Implicate Membrane Binding. ACS Chem Biol. 2020; doi: 10.1021/acschembio.0c00339.

79. Newberry RW, Leong JT, Chow ED, Kampmann M, DeGrado WF. Deep mutational scanning reveals the structural basis for α-synuclein activity. Nat Chem Biol. 2020; doi: 10.1038/s41589-020-0480-6.

80. Roscoe BP, Bolon DNA. Systematic Exploration of Ubiquitin Sequence, E1 Activation Efficiency, and Experimental Fitness in Yeast. Journal of Molecular Biology. 2014; doi: 10.1016/j.jmb.2014.05.019.

81. Sarkisyan KS, Bolotin DA, Meer MV, Usmanova DR, Mishin AS, Sharonov GV, et al.. Local fitness landscape of the green fluorescent protein. Nature. Nature Publishing Group; 2016; doi: 10.1038/nature17995.

82. Silverstein RA, Sun S, Verby M, Weile J, Wu Y, Roth FP. A systematic genotype-phenotype map for missense variants in the human intellectual disability-associated gene GDI1. bioRxiv;

83. Starita LM, Pruneda JN, Lo RS, Fowler DM, Kim HJ, Hiatt JB, et al.. Activity-enhancing mutations in an E3 ubiquitin ligase identified by high-throughput mutagenesis. PNAS. 2013; doi: 10.1073/pnas.1303309110.

84. Starita LM, Young DL, Islam M, Kitzman JO, Gullingsrud J, Hause RJ, et al.. Massively Parallel Functional Analysis of BRCA1 RING Domain Variants. Genetics. 2015; doi: 10.1534/genetics.115.175802.

85. Starita LM, Islam MM, Banerjee T, Adamovich AI, Gullingsrud J, Fields S, et al.. A Multiplex Homology-Directed DNA Repair Assay Reveals the Impact of More Than 1,000 BRCA1 Missense Substitution Variants on Protein Function. The American Journal of Human Genetics. 2018; doi: 10.1016/j.ajhg.2018.07.016.

86. Suiter CC, Moriyama T, Matreyek KA, Yang W, Scaletti ER, Nishii R, et al.. Massively parallel variant characterization identifies NUDT15 alleles associated with thiopurine toxicity. Proc Natl Acad Sci USA. 2020; doi: 10.1073/pnas.1915680117.

87. Sun S, Weile J, Verby M, Wu Y, Wang Y, Cote AG, et al.. A proactive genotype-to-patient-phenotype map for cystathionine beta-synthase. Genome Med. 2020; doi: 10.1186/s13073-020-0711-1.

88. Thompson S, Zhang Y, Ingle C, Reynolds KA, Kortemme T. Altered expression of a quality control protease in E. coli reshapes the in vivo mutational landscape of a model enzyme. eLife. 2020; doi: 10.7554/eLife.53476.

89. Trenker R, Wu X, Nguyen JV, Wilcox S, Rubin AF, Call ME, et al.. Human and viral membrane–associated E3 ubiquitin ligases MARCH1 and MIR2 recognize different features of CD86 to downregulate surface expression. Journal of Biological Chemistry. Elsevier; 2021; doi: 10.1016/j.jbc.2021.100900.

90. Weile J, Sun S, Cote AG, Knapp J, Verby M, Mellor JC, et al.. A framework for exhaustively mapping functional missense variants. Mol Syst Biol. 2017; doi: 10.15252/msb.20177908.

91. Weile J, Kishore N, Sun S, Maaieh R, Verby M, Li R, et al.. Shifting landscapes of human MTHFR missense-variant effects. The American Journal of Human Genetics. Elsevier; 2021; doi: 10.1016/j.ajhg.2021.05.009.

92. Wrenbeck EE, Bedewitz MA, Klesmith JR, Noshin S, Barry CS, Whitehead TA. An Automated Data-Driven Pipeline for Improving Heterologous Enzyme Expression. ACS Synth Bi-ol. American Chemical Society; 2019; doi: 10.1021/acssynbio.8b00486.

93. Zhang L, Sarangi V, Moon I, Yu J, Liu D, Devarajan S, et al.. CYP2C9 and CYP2C19: Deep Mutational Scanning and Functional Characterization of Genomic Missense Variants. Clinical and Translational Science. 2020; doi: https://doi.org/10.1111/cts.12758.

94. Zinkus-Boltz J, DeValk C, Dickinson BC. A Phage-Assisted Continuous Selection Approach for Deep Mutational Scanning of Protein–Protein Interactions. ACS Chem Biol. American Chemical Society; 2019; doi: 10.1021/acschembio.9b00669.

95. Bernier-Villamor V, Sampson DA, Matunis MJ, Lima CD. Structural Basis for E2-Mediated SUMO Conjugation Revealed by a Complex between Ubiquitin-Conjugating Enzyme Ubc9 and RanGAP. Cell. 108:122002;

96. Blanpain C, Doranz BJ, Vakili J, Rucker J, Govaerts C, Baik SSW, et al.. Multiple Charged and Aromatic Residues in CCR5 Amino-terminal Domain Are Involved in High Af-finity Binding of Both Chemokines and HIV-1 Env Protein. J Biol Chem. 1999; doi: 10.1074/jbc.274.49.34719.

97. Brzovic PS, Keeffe JR, Nishikawa H, Miyamoto K, Fox D, Fukuda M, et al.. Binding and recognition in the assembly of an active BRCA1/BARD1 ubiquitin-ligase complex. Proceedings of the National Academy of Sciences. 2003; doi: 10.1073/pnas.0836054100.

98. Chen S, Wu J, Zhong S, Li Y, Zhang P, Ma J, et al.. iASPP mediates p53 selectivity through a modular mechanism fine-tuning DNA recognition. Proc Natl Acad Sci USA. 2019; doi: 10.1073/pnas.1909393116.

99. Chupreta S, Holmstrom S, Subramanian L, Iñiguez-Lluhí JA. A Small Conserved Surface in SUMO Is the Critical Structural Determinant of Its Transcriptional Inhibitory Properties. MCB. 2005; doi: 10.1128/MCB.25.10.4272-4282.2005.

100. Cobb JA, Roberts DM. Structural Requirements for N-Trimethylation of Lysine 115 of Calmodulin. Journal of Biological Chemistry. 2000; doi: 10.1074/jbc.M002332200.

101. Coyne RS, McDonald HB, Edgemon K, Brody LC. Functional Characterization of BRCA1 Sequence Variants using a Yeast Small Colony Phenotype Assay. Cancer Biology & Therapy. 2004; doi: 10.4161/cbt.3.5.809.

102. Denker K, Orlik F, Schiffler B, Benz R. Site-directed Mutagenesis of the Greasy Slide Aromatic Residues Within the LamB (Maltoporin) Channel of Escherichia coli: Effect on Ion and Maltopentaose Transport. Journal of Molecular Biology. 2005; doi: 10.1016/j.jmb.2005.07.025.

103. Dragic T, Trkola A, Lin SW, Nagashima KA, Kajumo F, Zhao L, et al.. Amino-Terminal Substitutions in the CCR5 Coreceptor Impair gp120 Binding and Human Immunodeficiency Virus Type 1 Entry. J Virol. 1998; doi: 10.1128/JVI.72.1.279-285.1998.

104. Dragic T, Trkola A, Thompson DAD, Cormier EG, Kajumo FA, Maxwell E, et al.. A binding pocket for a small molecule inhibitor of HIV-1 entry within the transmembrane helices of CCR5. Proceedings of the National Academy of Sciences. 2000; doi: 10.1073/pnas.090576697.

105. Ecsédi P, Gógl G, Hóf H, Kiss B, Harmat V, Nyitray L. Structure Determination of the Transactivation Domain of p53 in Complex with S100A4 Using Annexin A2 as a Crystallization Chaperone. Structure. 2020; doi: 10.1016/j.str.2020.05.001.

106. Kopecká J, Krijt J, Raková K, Kožich V. Restoring assembly and activity of cystathionine β-synthase mutants by ligands and chemical chaperones. Journal of Inherited Metabolic Disease. 2011; doi: 10.1007/s10545-010-9087-5.

107. Kožich V, Sokolová J, Klatovská V, Krijt J, Janošík M, Jelínek K, et al.. Cystathionine β-synthase mutations: effect of mutation topology on folding and activity. Hum Mutat. 2010; doi: 10.1002/humu.21273.

108. Kruger W d., Wang L, Jhee K h., Singh R h., Elsas II L j.. Cystathionine β-synthase deficiency in Georgia (USA): Correlation of clinical and biochemical phenotype with genotype. Human Mutation. 2003; doi: 10.1002/humu.10290.

109. Lee SY, Pullen L, Virgil DJ, Castañeda CA, Abeykoon D, Bolon DNA, et al.. Alanine Scan of Core Positions in Ubiquitin Reveals Links between Dynamics, Stability, and Function. Journal of Molecular Biology. 2014; doi: 10.1016/j.jmb.2013.10.042.

110. Li W, Zhang C, Sui J, Kuhn JH, Moore MJ, Luo S, et al.. Receptor and viral determinants of SARS-coronavirus adaptation to human ACE2. EMBO J. 2005; doi: 10.1038/sj.emboj.7600640.

111. Lin G, Baribaud F, Romano J, Doms RW, Hoxie JA. Identification of gp120 Binding Sites on CXCR4 by Using CD4-Independent Human Immunodeficiency Virus Type 2 Env Proteins. JVI. 2003; doi: 10.1128/JVI.77.2.931-942.2003.

112. Mascle XH, Lussier-Price M, Cappadocia L, Estephan P, Raiola L, Omichinski JG, et al.. Identification of a Non-covalent Ternary Complex Formed by PIAS1, SUMO1, and UBC9 Proteins Involved in Transcriptional Regulation. Journal of Biological Chemistry. 2013; doi: 10.1074/jbc.M113.486845.

113. Matthews EE, Thévenin D, Rogers JM, Gotow L, Lira PD, Reiter LA, et al.. Thrombopoietin receptor activation: transmembrane helix dimerization, rotation, and allosteric modulation. The FASEB Journal. 2011; doi: https://doi.org/10.1096/fj.10-178673.

114. Mayfield JA, Davies MW, Dimster-Denk D, Pleskac N, McCarthy S, Boydston EA, et al.. Surrogate Genetics and Metabolic Profiling for Characterization of Human Disease Alleles. Genetics. 2012; doi: 10.1534/genetics.111.137471.

115. Navenot J-M, Wang Z, Trent JO, Murray JL, Hu Q, DeLeeuw L, et al.. Molecular anatomy of CCR5 engagement by physiologic and viral chemokines and HIV-1 envelope glycoproteins: differences in primary structural requirements for RANTES, MIP-1α, and vMIP-II binding11Edited by P. E. Wright. Journal of Molecular Biology. 2001; doi: 10.1006/jmbi.2001.5086.

116. Peng L, Damschroder MM, Cook KE, Wu H, Dall'Acqua WF. Molecular basis for the antagonistic activity of an anti-CXCR4 antibody. mAbs. 2016; doi: 10.1080/19420862.2015.1113359.

117. Peterson BR, Sun LJ, Verdine GL. A critical arginine residue mediates cooperativity in the contact interface between transcription factors NFAT and AP-1. Proceedings of the National Academy of Sciences. 1996; doi: 10.1073/pnas.93.24.13671.

118. Rabut GEE, Konner JA, Kajumo F, Moore JP, Dragic T. Alanine Substitutions of Polar and Nonpolar Residues in the Amino-Terminal Domain of CCR5 Differently Impair Entry of Macrophage- and Dualtropic Isolates of Human Immunodeficiency Virus Type 1. J Virol. 1998; doi: 10.1128/JVI.72.4.3464-3468.1998.

119. Ransburgh DJR, Chiba N, Ishioka C, Toland AE, Parvin JD. Identification of Breast Tumor Mutations in BRCA1 That Abolish Its Function in Homologous DNA Recombination. Cancer Res. 2010; doi: 10.1158/0008-5472.CAN-09-2850.

120. Tan Y, Tong P, Wang J, Zhao L, Li J, Yu Y, et al.. The Membrane-Proximal Region of C–C Chemokine Receptor Type 5 Participates in the Infection of HIV-1. Front Immunol. 2017; doi: 10.3389/fimmu.2017.00478.

121. Towler WI, Zhang J, Ransburgh DJR, Toland AE, Ishioka C, Chiba N, et al.. Analysis of BRCA1 Variants in Double-Strand Break Repair by Homologous Recombination and Single-Strand Annealing. Human Mutation. 2013; doi: 10.1002/humu.22251.

122. Trent JO, Wang Z, Murray JL, Shao W, Tamamura H, Fujii N, et al.. Lipid Bilayer Simu-lations of CXCR4 with Inverse Agonists and Weak Partial Agonists. J Biol Chem. 2003; doi: 10.1074/jbc.M307850200.

123. Van Gelder P, Dumas F, Bartoldus I, Saint N, Prilipov A, Winterhalter M, et al.. Sugar Transport through Maltoporin of Escherichia coli : Role of the Greasy Slide. J Bacteriol. 2002; doi: 10.1128/JB.184.11.2994-2999.2002.

124. VanBerkum MF, Means AR. Three amino acid substitutions in domain I of calmodulin prevent the activation of chicken smooth muscle myosin light chain kinase. J Biol Chem. Amer-ican Society for Biochemistry and Molecular Biology; 266:21488–951991;

125. Wei Q, Wang L, Wang Q, Kruger WD, Dunbrack RL. Testing computational prediction of missense mutation phenotypes: Functional characterization of 204 mutations of human cysta-thionine beta synthase. Proteins: Structure, Function, and Bioinformatics. 2010; doi: 10.1002/prot.22722.

126. Williams AD, Shivaprasad S, Wetzel R. Alanine Scanning Mutagenesis of Aβ(1-40) Am-yloid Fibril Stability. Journal of Molecular Biology. 2006; doi: 10.1016/j.jmb.2006.01.041.

127. Zhang J, Rao E, Dioszegi M, Kondru R, DeRosier A, Chan E, et al.. The Second Extracel-lular Loop of CCR5 Contains the Dominant Epitopes for Highly Potent Anti-Human Immuno-deficiency Virus Monoclonal Antibodies. AAC. 2007; doi: 10.1128/AAC.01302-06.

128. Nelsen RB. An introduction to copulas. 2nd ed. New York: Springer;

129. Bedő J, Ong CS. Multivariate Spearman's rho for aggregating ranks using copulas. Journal of Machine Learning Research. arXiv; 2016; doi: 10.48550/ARXIV.1410.4391.

~~129~~130. Pedregosa F, Varoquaux G, Gramfort A, Michel V, Thirion B, Grisel O, et al.. Scikit-learn: Machine Learning in Python. Journal of machine Learning research. :2825–30 2011;

131. Hunter JD. Matplotlib: A 2D Graphics Environment. Computing in Science & Engineering. 2007; doi: 10.1109/MCSE.2007.55.

132. Choi Y, Sims GE, Murphy S, Miller JR, Chan AP. Predicting the Functional Effect of Amino Acid Substitutions and Indels. de Brevern AG, editor. PLoS ONE. 2012; doi: 10.1371/journal.pone.0046688.

133. Vaser et al.. SIFT missense predictions for genomes. Nature Protocols. 2016;

134. Adzhubei IA, Schmidt S, Peshkin L, Ramensky VE, Gerasimova A, Bork P, et al.. A method and server for predicting damaging missense mutations. Nature Methods. 2010; doi: 10.1038/nmeth0410-248.

135. Laine E, Karami Y, Carbone A. GEMME: A Simple and Fast Global Epistatic Model Predicting Mutational Effects. Molecular Biology and Evolution. 2019; doi: 10.1093/molbev/msz179.

136. Meier J, Rao R, Verkuil R, Liu J, Sercu T, Rives A. Language models enable zero-shot prediction of the effects of mutations on protein function. bioRxiv;

137. Frazer J, Notin P, Dias M, Gomez A, Min JK, Brock K, et al.. Disease variant prediction with deep generative models of evolutionary data. Nature. 2021; doi: 10.1038/s41586-021-04043-8.

138. Livesey BJ, Marsh JA. Updated benchmarking of variant effect predictors using deep mu-tational scanning. bioRxiv;

~~130~~139. González J, Dai Z, Hennig P, Lawrence ND. Batch Bayesian Optimization via Local Pe-nalization. arXiv;

Supplementary material

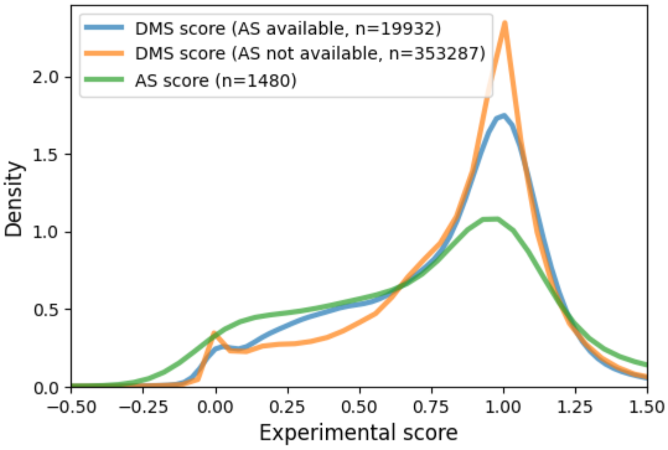

Fig S1. DMS and AS score distribution. The figure shows the kernel estimated density of normalized AS scores and DMS scores for variants with or without available AS data.

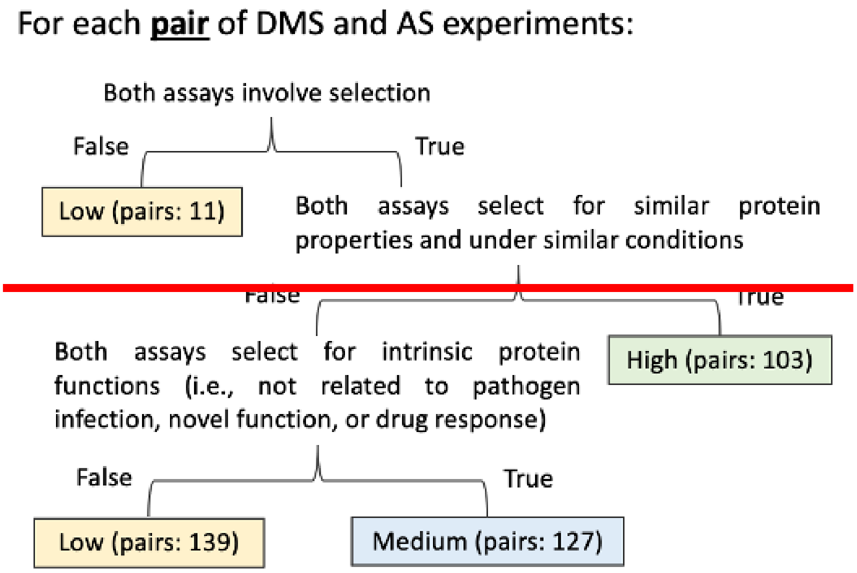

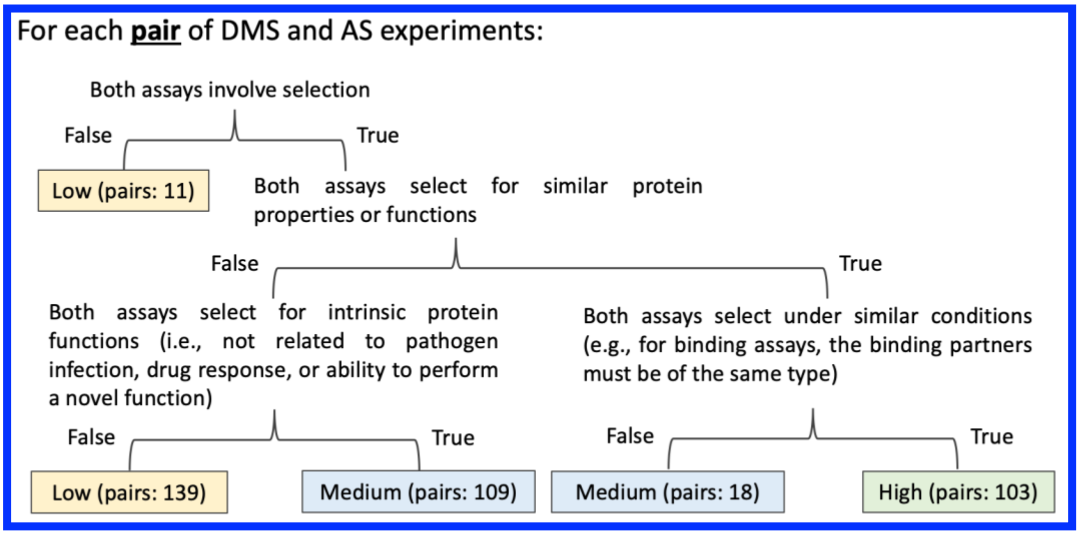

Fig S2. Decision tree for classifying ~~the~~ DMS and AS assay compatibility. The ~~end-nodes~~ similarity of DMS and AS assays are compared (Methods) and the DMS/AS assay pairs are classified using three levels of compatibility (low, medium, high). The leaf-node text and color show the classified assay compatibility. The number indicates the count of assay pairs for each compatibility level ~~(low, medium, high)~~.

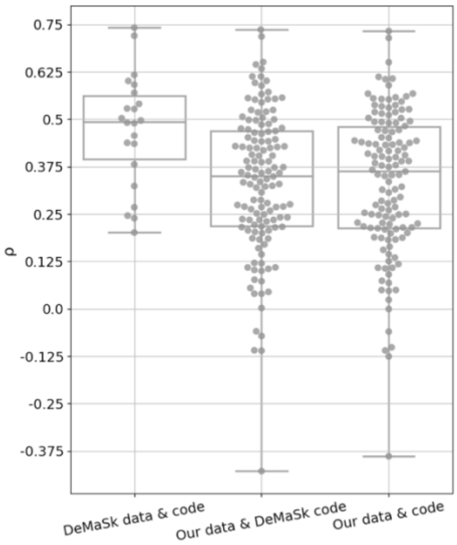

Fig S3. Comparison between published and re-implemented predictors. The plot shows leave-one-protein-out cross-validation performance on predictors built from the published DeMaSk code or our code. The predictors were trained and evaluated on DMS data either provided by the DeMaSk study or curated by our own. The "DeMaSk data & code" result is similar to the published result. For the "Our data & DeMaSk code" result, we used our own data and published code which shows a median performance around 0.35. This is probably because many more DMS results are included in our data. The similarity of results achieved using "Our data & code" demonstrates the correctness of our re-implementation. (Whiskers show the full value range)

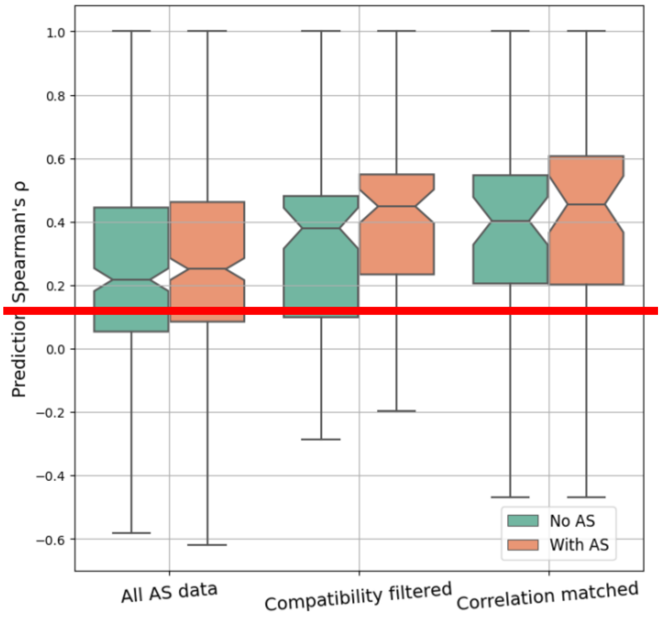

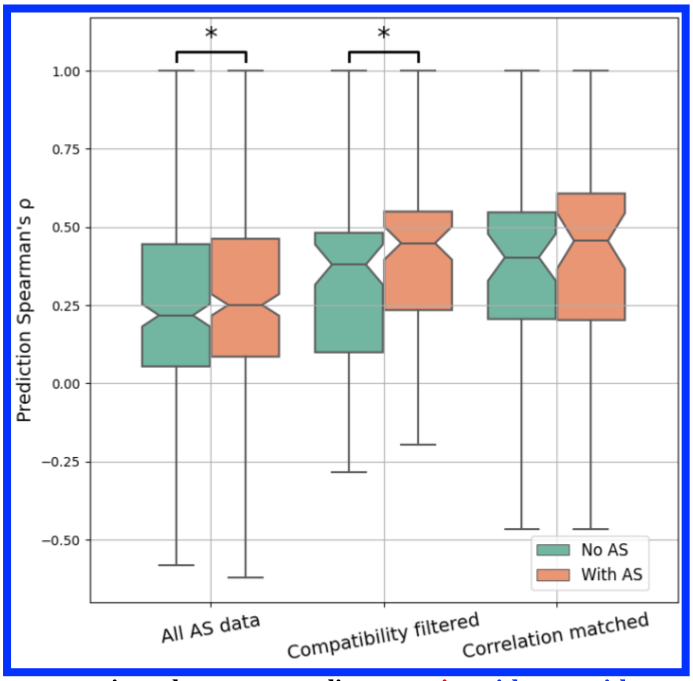

Fig S4. Performance comparison between predictors ~~using~~with or without AS data~~ or not~~. The Spearman's ρ between ~~experiment~~ DMS scores and predicted scores for each DMS and AS data pair are shown as box plots. Different approaches to filtering~~/matching~~ the data are shown on the x-axis: "All AS data" used all available data; "Compatibility filtered" used only data of high assay compatibility; "Correlation matched" used only data with the highest regularised correlation for each DMS dataset. The figure does not include data without available ~~(filtered/matched)~~ AS scores. This means that the different results are not directly comparable since they are ~~visualized on~~computed for different subsets of DMS/AS data pairs (for example, "All AS data" contains all DMS/AS data pairs, but "Compatibility filtered" contains only data pairs of high assay compatibility). Control

results are shown as green boxes for ~~predicting~~predictions on the same residues without AS data as a feature. The underlying ρ for each data pair in the control results is the same, but the boxes are shifted due to data filtering~~/matching~~. Results for data pairs with only one residue are not shown. P-values were calculated using paired t-test and jointly corrected using Holm-Šidák (Methods), *: $p<0.05$. Notches show the 95% confidence interval around the median, and whiskers show the full value range.

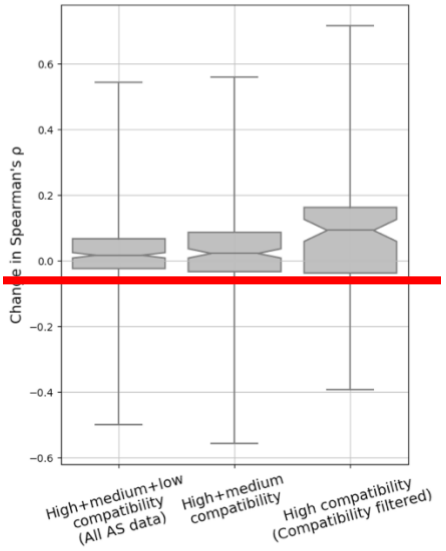

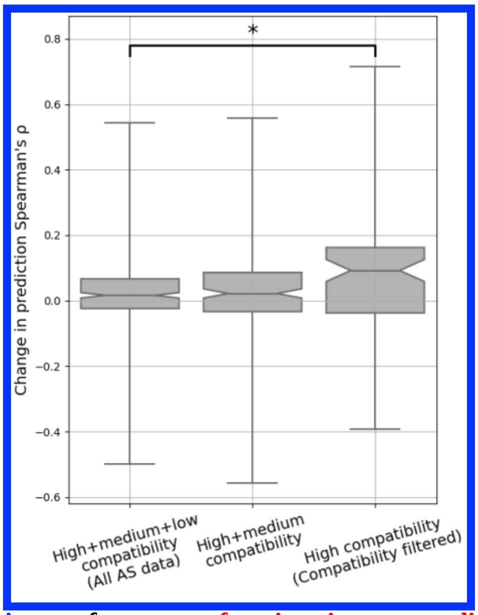

Fig S5. The change in prediction performance ~~of variant impact prediction~~ for using data of different assay compatibility levels. The change of prediction Spearman's ρ for each DMS and AS data pair is shown as box plots. A higher value represents higher prediction accuracy achieved for using AS data. Different data filtering methods are shown on the x-axis. Results for data pairs with only one residue are not shown. P-values were

calculated using Welch's test and jointly corrected using Holm-Šidák (Methods), *: $p<0.05$. Notches show the 95% confidence interval around the median, and whiskers show the full value range.

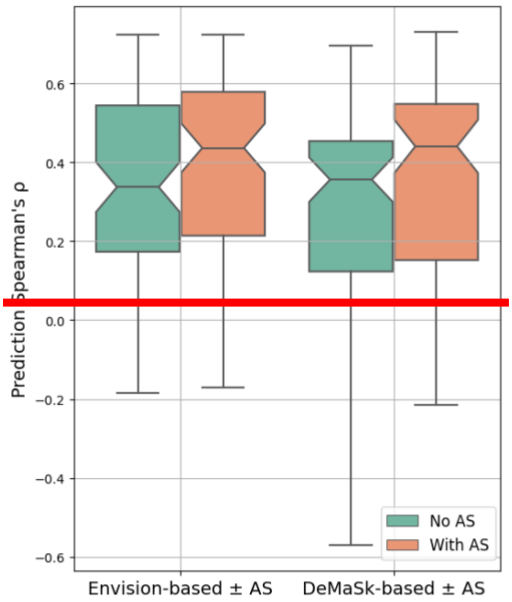

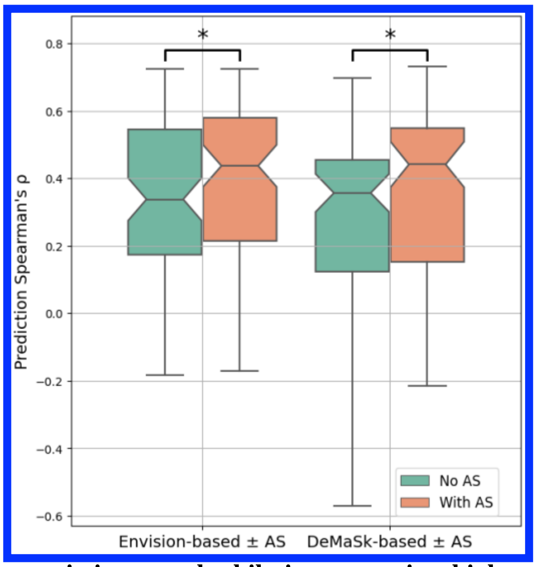

Fig S6. Prediction performance is improved while incorporating high compatibility AS data into the Envision model. The Spearman's ρ between experiment DMS scores and predicted scores for each ~~high compatible~~ DMS/AS assay pair with high compatibility are shown as box plots. The x-axis shows the predictor used, either Envision or DeMaSk. Control results are shown as green boxes for ~~predicting without AS data as a feature~~predictions on the same residues without AS data as a feature. Results for data pairs with only one residue are not shown. P-values were calculated using paired t-test and jointly corrected using Holm-Šidák (Methods), *: $p<0.05$. Notches show the 95% confidence interval around the median, and whiskers show the full value range.

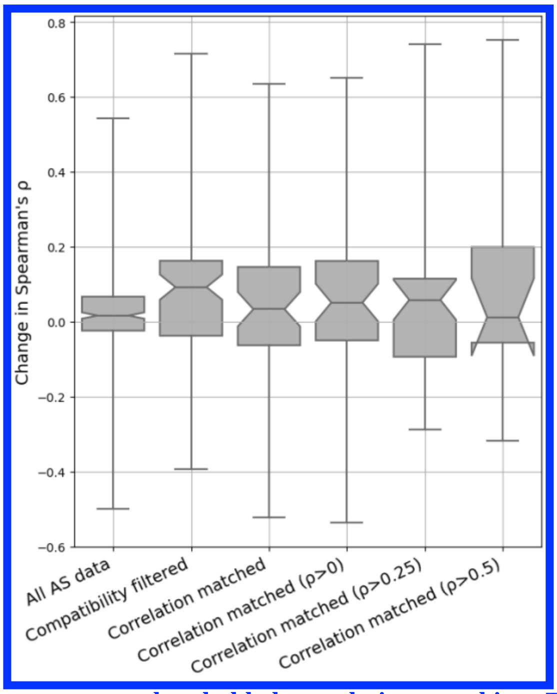

Fig S7. Performance improvement on thresholded correlation matching. The change of prediction ρ for each DMS and AS data pair is shown as box plots. Different approaches to filtering/matching the data are shown on the x-axis: "All AS data", "Compatibility filtered" and "Correlation matched" are the same results as previously discussed; while doing correlation matching, a further thresholding (0, 0.25 or 0.5) on the regularized DMS/AS correlation values (ρ_r) was applied. Notches show the 95% confidence interval around the median, and whiskers show the full value range.

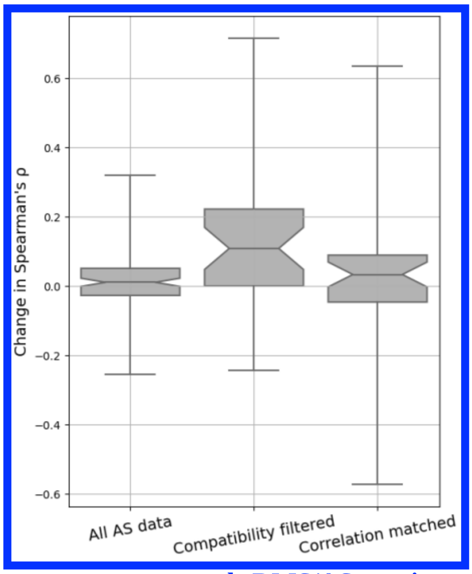

Fig S8. Performance improvement on averaged DMS/AS testing data. This figure shows model performance when we averaged variant scores for DMS or AS data that are: i) published in the same paper; ii)

targeting the same protein region; iii) measured by the same type of assays (Supplementary Table 1). The change of prediction ρ for each averaged DMS and AS data pair is shown. A higher value represents higher prediction accuracy achieved when using AS data. Different approaches to filtering/matching the data are shown on the x-axis: “All AS data” used all available data; “Compatibility filtered” used only data of high assay compatibility; “Correlation matched” used only data with the highest regularised correlation for each DMS dataset. Results for data pairs with only one residue are not shown. Notches show the 95% confidence interval around the median, and whiskers show the full value range.

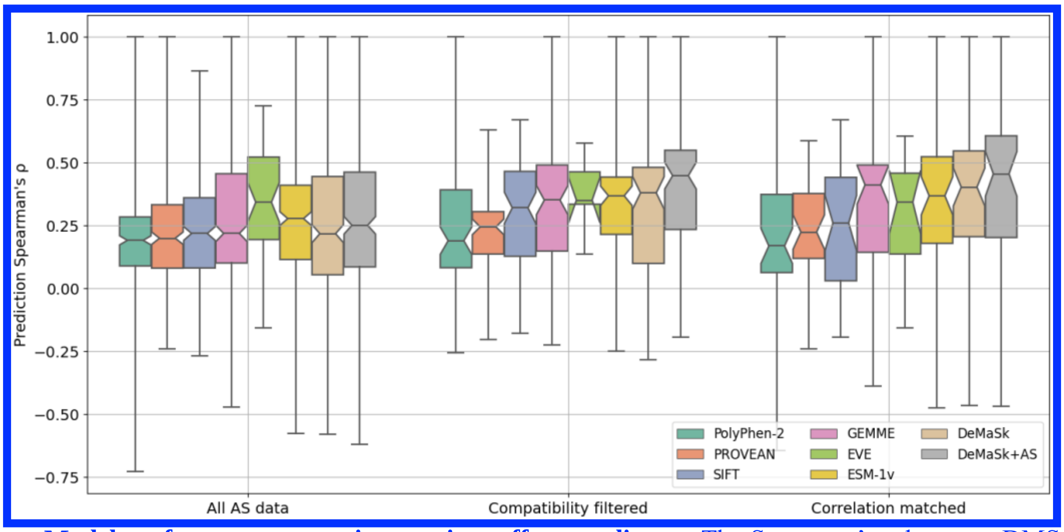

Fig S9. Model performance on various variant effect predictors. The Spearman’s ρ between DMS scores and predicted scores from different variant effect predictors for each DMS and AS pair are shown as box plots. Results are evaluated on different sets of variant data shown on the x-axis: “All AS data” used all available data; “Compatibility filtered” used only data of high assay compatibility; “Correlation matched” used only AS data with the highest regularised correlation for each DMS dataset. The figure does not include residues without available AS scores. Results for data pairs with only one residue are not shown. Notches show the 95% confidence interval around the median, and whiskers show the full value range.

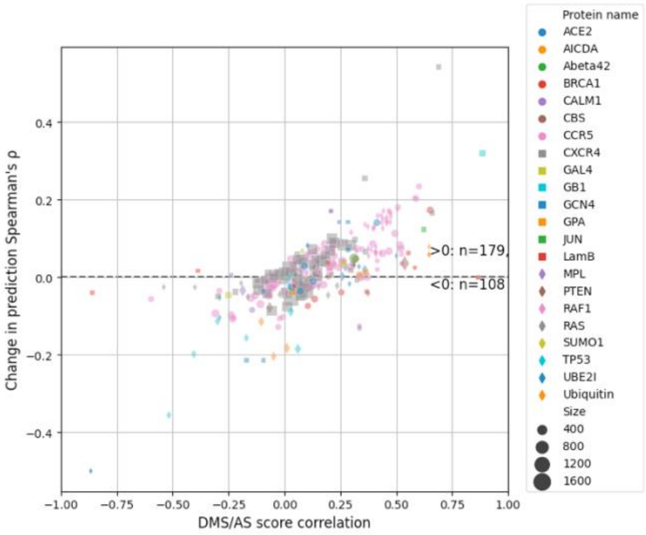

Fig S10. ~~**Fig S7.**~~ **Prediction performance change for using all AS data.** Each dot represents a DMS/AS data pair. The vertical axis shows the change of prediction ρ by using AS data (larger means higher performance achieved by using AS data). The horizontal axis shows the DMS/AS score correlation for *all* variants on the matched residues rather than just alanine substitutions. The colours and shapes of the dots correspond to the target protein, and size indicates the number of variants in each data pair. Results for data pairs with only one residue are not shown.

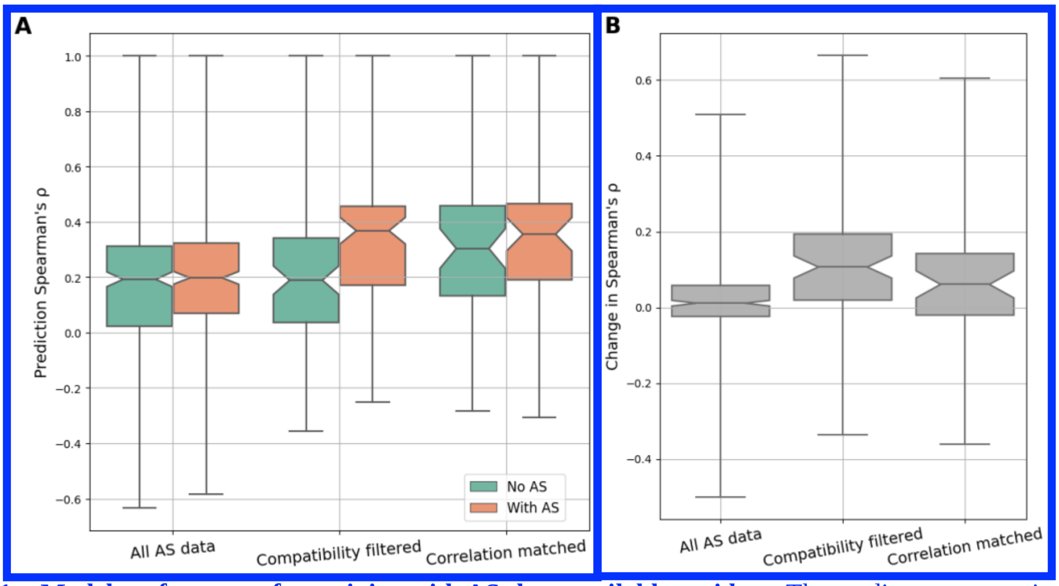

Fig S11. Model performance for training with AS-data-available-residues. The predictors were trained only on variants that have AS data available. Panel A shows the performance visualized by prediction Spearman's ρ for DMS scores and predicted scores for each DMS and AS data pair. Different approaches to filtering the data are shown on the x-axis: "All AS data" used all available data; "Compatibility filtered" used only data of high

assay compatibility; “Correlation matched” used only AS data with the highest regularised correlation for each DMS dataset. Control results are shown as green boxes for predictions on the same residues without AS data as a feature. Panel B shows change of prediction ρ for each DMS and AS data pair. A higher value indicates higher prediction accuracy achieved when using AS data. Different approaches to filtering the data are also shown on the x-axis as described. Notches show the 95% confidence interval around the median, and whiskers show the full value range.

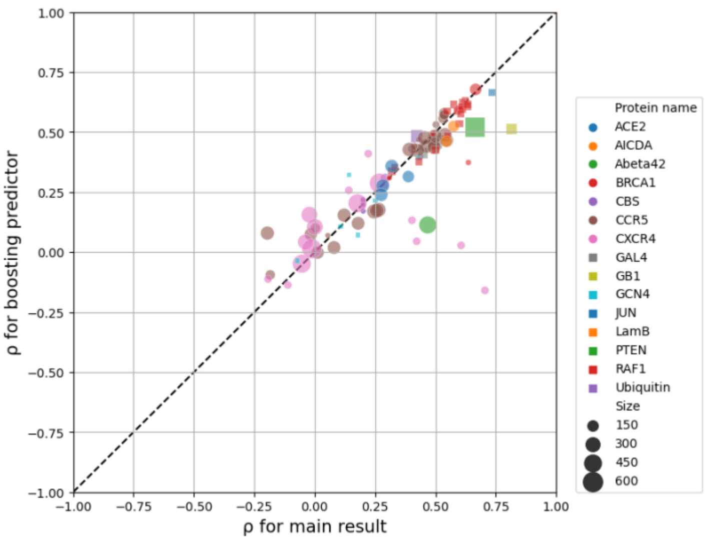

Fig S12. ~~**Fig S8.**~~ **Boosting setup shows similar performance as the main result.** Each dot represents a filtered DMS/AS data pair of high assay compatibility. The vertical and horizontal axes show the prediction Spearman’s ρ for either modelled with boosting or the one-step (main result) setup. The colours and shapes of the dots correspond to the target protein, and size indicates the number of variants in each data pair.

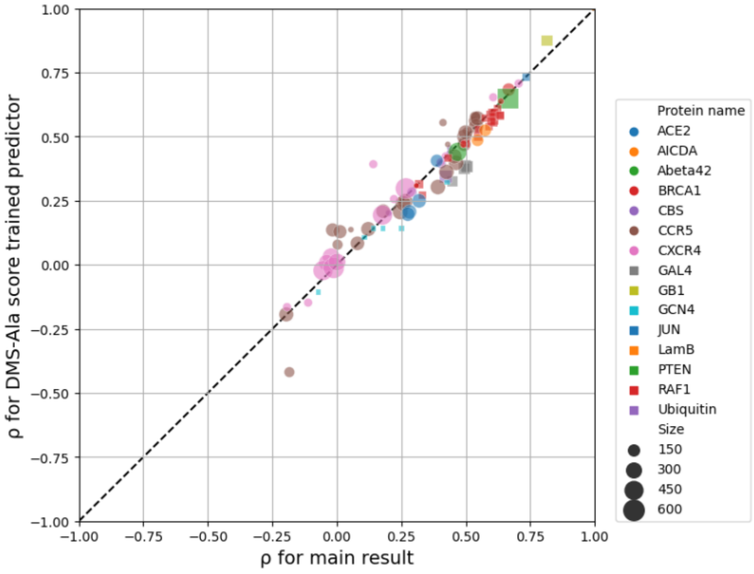

Fig S13. ~~**Fig S9.**~~ **Training with DMS scores of alanine substitutions shows similar performance as the main result.** The vertical and horizontal axes show the prediction Spearman's ρ for predictors either trained with DMS score of alanine substitutions (DMS-Ala) or AS data of high assay compatibility (main result), yet all evaluated on high compatibility AS data. The colours and shapes of the dots correspond to the target protein, and size indicates the number of variants in each data pair.

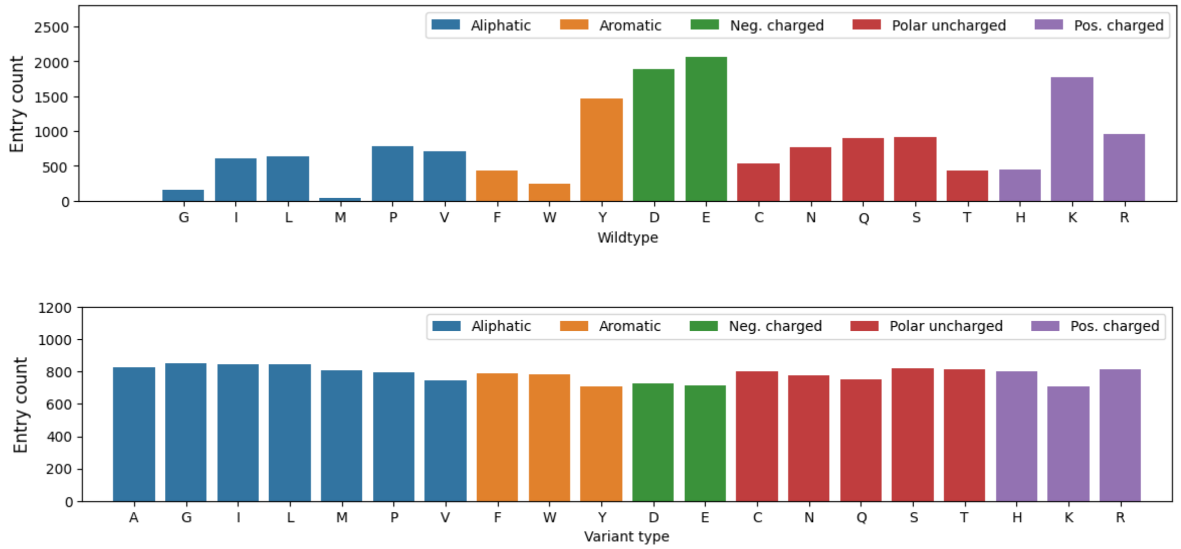

Fig S14. ~~**Fig S10.**~~ **Count of variant entries for each wild-type or variant amino acid of high assay compatibility data.** (Neg.: negatively, Pos.: positively)

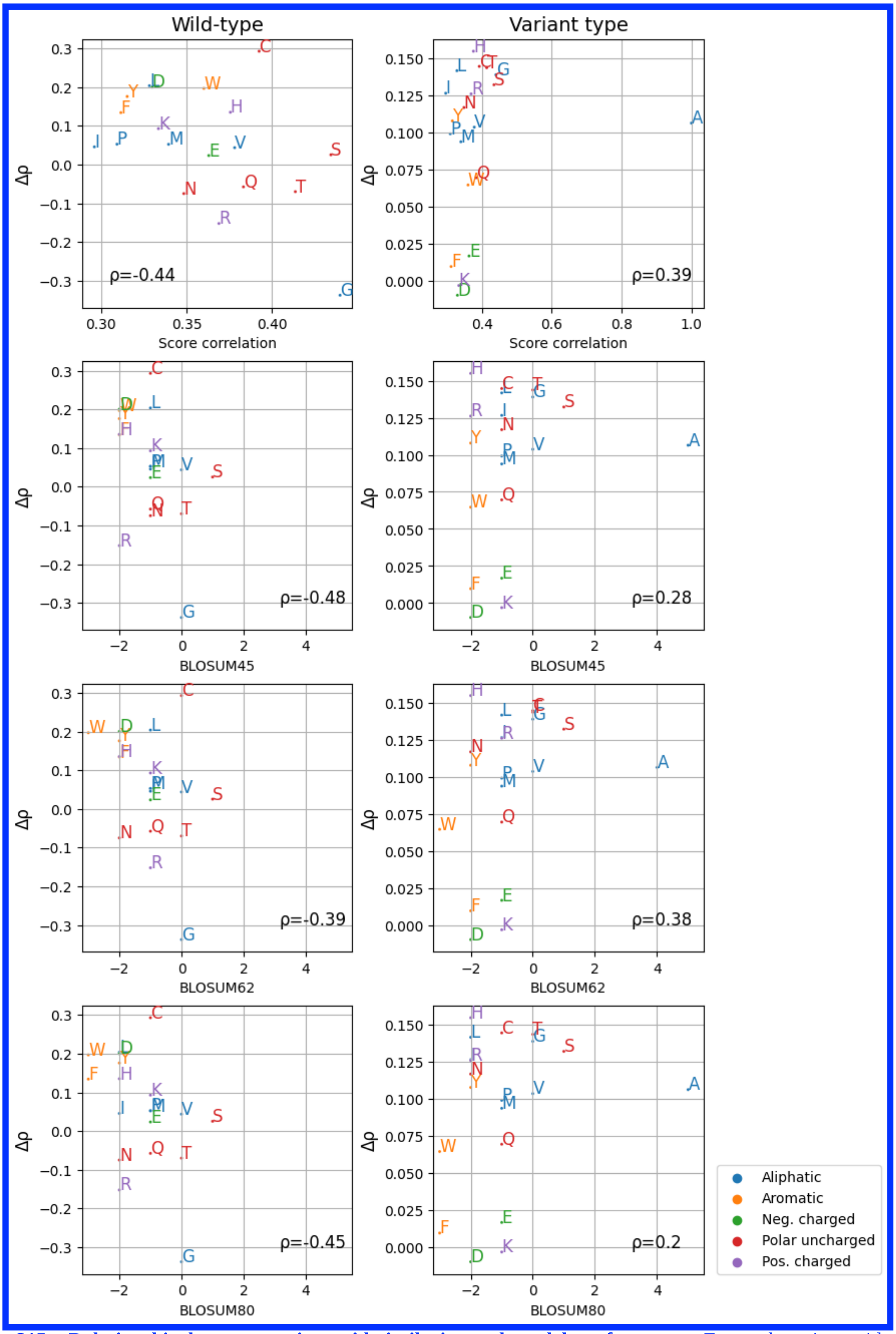

Fig S15. Relationship between amino acid similarity and model performance. For each amino acid, its similarity to alanine was computed by their DMS score correlation or using BLOSUM scores as shown on the x-

axis. The performance improvement (Δρ) for each wild-type (left) or variant (right) amino acid while using AS data were computed as previously mentioned (Fig 7), with their Spearman's correlation against the similarity measurements shown on the figure. The label for each amino acid is coloured by the amino acid physicochemical property. (Neg.: negatively; Pos.: positively)

Table S1. DMS/AS correlation on each secondary structural region. The secondary structure of each variant is determined by UniProt annotations. The Spearman's correlation between DMS and all or high compatibility AS data on each structural region is computed, with the number of protein residues involved shown in parenthesis.

**ρ (n_residues)**	**HELIX**	**STRAND**	**TURN**
All AS	0.13 (233)	0.13 (83)	0.17 (22)
AS of high compatibility	0.28 (115)	0.26 (56)	0.41 (15)

Table S2. ~~Table S1.~~ Amount of data with AS scores available

**Data composition**	**Protein**	**DMS dataset**	**AS dataset[1]**	**Variant entries[2]**
All AS	22	54	146	70446
Compatibility filtered	15	35	60	15739
High+medium assay compatibility	21	51	105	28380
Correlation matched	22	54	32	7940

1. This column shows how many unique AS datasets are included.
2. Include duplicated variants caused by multiple experiments targeting the same protein variant.

Supplementary information

Applying AS data to Envision method

We re-implemented a predictor based on Envision ~~[15]~~[17] to incorporate AS data. Features used in Envision were downloaded from its online toolkit. All Envision features are used for

modelling except for substitution type (wt_mut) which has low importance according to the published result and our pilot studies yet is computationally expensive in our setup. Protein data were excluded if their features were not available online. DMS and AS data pairs with high assay compatibility were used for modelling. Missing feature values were imputed by the mean values for numerical features or the most frequent values for categorical features. Categorical features are encoded with the one-hot encoder. We used `sklearn.ensemble.GradientBoostingRegressor` from scikit-learn package ~~[129]~~[130] to build the predictor, and hyperparameters were tuned by Bayesian Optimization ~~[130]~~[139] with Group K-Fold (protein-30-fold) cross-validation. The training and evaluation process were similar to that previously described. For comparison, we repeated the DeMaSk-based analysis on the same subset of data.

Boosting with AS data

To deal with the sparsity of AS data, we tested a variant impact predictor based on boosting. A first linear regression predictor was trained with all training DMS data using the three DeMaSk features without AS data, which was the same as the control predictor mentioned previously. We then calculated the prediction error by subtracting the predicted scores from DMS scores, and a second linear regression predictor was trained to predict the error. The second predictor was trained only on DMS/AS data of high assay compatibility and used both protein features and the encoded AS scores. The final prediction result was the sum of the outputs from these two predictors.

Replacing AS data with DMS scores of alanine substitutions

We investigated another potential approach to overcome the sparsity of AS data by replacing the AS feature with the DMS scores of alanine substitutions (DMS-Ala). ~~For~~ The intention of

this study is to model the scenario of ideal AS data, which perfectly matches the DMS-Ala data during training. To do this, for all DMS datasets we collected, their AS feature values, regardless of availability, were replaced by the DMS-Ala scores on the same residue. Missing scores were imputed by the mean value of all DMS-Ala scores. A regression model was trained and evaluated as previously described, using the three DeMaSk features as well as the DMS-Ala scores. The AS data of high assay compatibility are still used for the testing process.

Supplementary Table 2

Click here to access/download
Supplementary Material
Supplementary_Table_2_Supplementary Matrerial.csv

Supplementary Table 3

Click here to access/download

Supplementary Material

Supplementary_Table_3_Supplementary Matrerial.csv

Supplementary Table 1

Click here to access/download
Supplementary Material
Supplementary_Table_1_Supplementary Material.xlsx
