# Supplementary material for: Integrating deep mutational scanning and low-throughput mutagenesis data to predict the impact of amino acid variants
Source: Gigascience. 2023 Sep 18;12:giad073. doi: 10.1093/gigascience/giad073 (PMC10506130; doi:10.1093/gigascience/giad073)
Supplement: giad073_Supplemental_Files [file giad073_supplemental_files.zip › Supplementary_Information.pdf]

Applying AS data to Envision method

We re-implemented a predictor based on Envision to incorporate AS data. Features used in Envision were downloaded from its online toolkit. All Envision features are used for modelling except for substitution type (wt_mut) which has low importance according to the published result and our pilot studies yet is computationally expensive in our setup. Protein data were excluded if their features were not available online. DMS and AS data pairs with high assay compatibility were used for modelling. Missing feature values were imputed by the mean values for numerical features or the most frequent values for categorical features. Categorical features are encoded with the one-hot encoder. We used `sklearn.ensemble.GradientBoostingRegressor` from scikit-learn package to build the predictor, and hyperparameters were tuned by Bayesian Optimization with Group K-Fold (protein-30-fold) cross-validation. The training and evaluation process were similar to that previously described. For comparison, we repeated the DeMaSk-based analysis on the same subset of data.

Boosting with AS data

To deal with the sparsity of AS data, we tested a variant impact predictor based on boosting. A first linear regression predictor was trained with all training DMS data using the three DeMaSk features without AS data, which was the same as the control predictor mentioned previously. We then calculated the prediction error by subtracting the predicted scores from DMS scores, and a second linear regression predictor was trained to predict the error. The second predictor was trained only on DMS/AS data of high assay compatibility and used both protein features and the encoded AS scores. The final prediction result was the sum of the outputs from these two predictors.

Replacing AS data with DMS scores of alanine substitutions

We investigated another potential approach to overcome the sparsity of AS data by replacing the AS feature with the DMS scores of alanine substitutions (DMS-Ala). The intention of this study is to model the scenario of ideal AS data, which perfectly matches the DMS-Ala data during training. To do this, for all DMS datasets we collected, their AS feature values, regardless of availability, were replaced by the DMS-Ala scores on the same residue. Missing scores were imputed by the mean value of all DMS-Ala scores. A regression model was trained and evaluated as previously described, using the three DeMaSk features as well as the DMS-Ala scores. The AS data of high assay compatibility are still used for the testing process.
