# Supplementary figures and images for: Integrating deep mutational scanning and low-throughput mutagenesis data to predict the impact of amino acid variants
Source: Gigascience. 2023 Sep 18;12:giad073. doi: 10.1093/gigascience/giad073 (PMC10506130; doi:10.1093/gigascience/giad073)

For each **pair** of DMS and AS experiments:

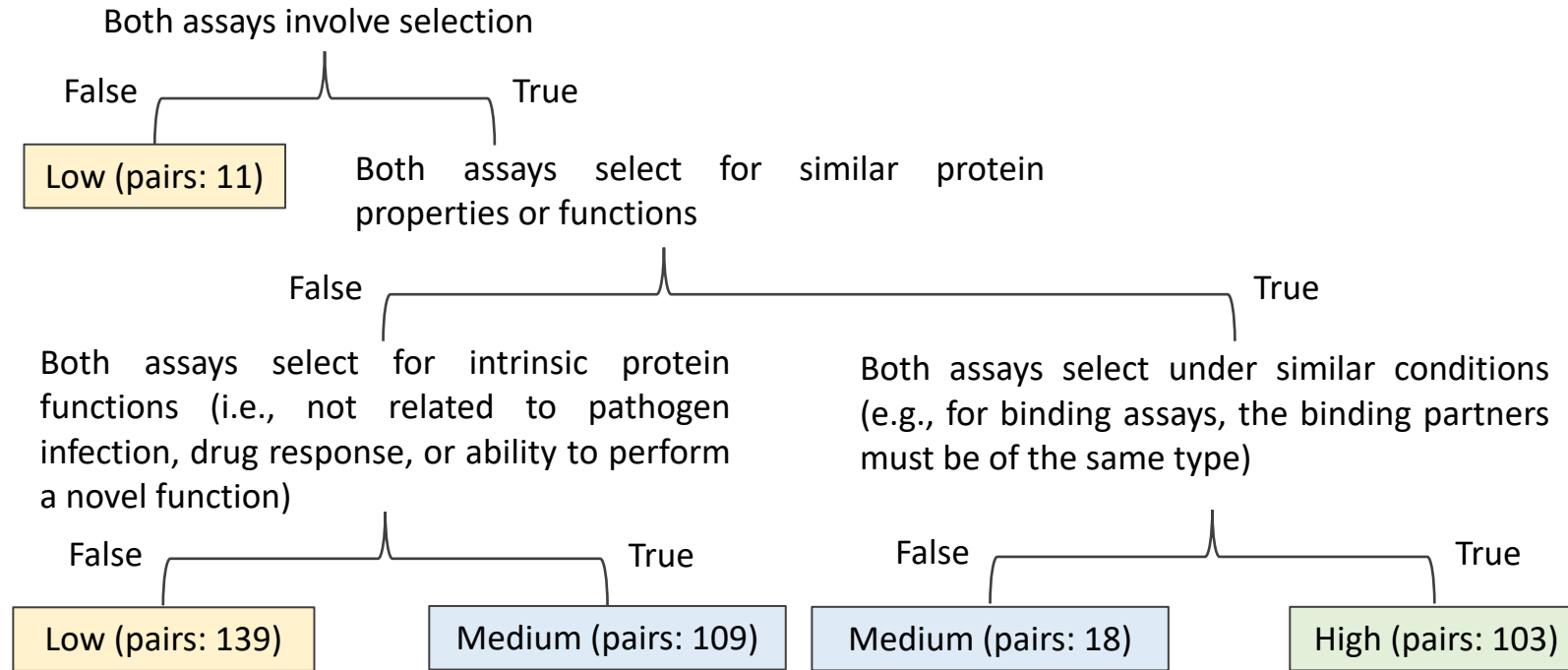

Supplement: giad073_Supplemental_Files [file giad073_supplemental_files.zip › Supplementary_Fig_S2.pdf]
