# Supplementary material for: Integrating deep mutational scanning and low-throughput mutagenesis data to predict the impact of amino acid variants
Source: Gigascience. 2023 Sep 18;12:giad073. doi: 10.1093/gigascience/giad073 (PMC10506130; doi:10.1093/gigascience/giad073)
Supplement: giad073_Response_to_Reviewer_Comments_Original_Submission [file giad073_response_to_reviewer_comments_original_submission.pdf]

Author's Response To Reviewer Comments

We thank the reviewers for their thoughtful comments, which helped us improve our study and the manuscript.

We have attached a detailed response to the reviews (Review_response_untracked.pdf) as well as a version of the updated manuscript with all changes from the original submission highlighted (diff_manuscript.pdf).
