# Supplementary material for: Integrating deep mutational scanning and low-throughput mutagenesis data to predict the impact of amino acid variants
Source: Gigascience. 2023 Sep 18;12:giad073. doi: 10.1093/gigascience/giad073 (PMC10506130; doi:10.1093/gigascience/giad073)
Supplement: giad073_Response_to_Reviewer_Comments_Revision_1 [file giad073_response_to_reviewer_comments_revision_1.pdf]

Author's Response To Reviewer Comments

We would like to thank the reviewers again for their thoughtful comments and careful consideration of the manuscript and revisions. We hope that this work contributes to the ongoing conversation in the field around modelling and analysis of protein mutagenesis data, and that the datasets we curated and present here will be useful for future studies.
