# Supplementary material for: Integrating deep mutational scanning and low-throughput mutagenesis data to predict the impact of amino acid variants
Source: Gigascience. 2023 Sep 18;12:giad073. doi: 10.1093/gigascience/giad073 (PMC10506130; doi:10.1093/gigascience/giad073)
Supplement: giad073_Reviewer_1_Report_Original_Submission — Joseph Ng -- 3/21/2023 Reviewed [file giad073_reviewer_1_report_original_submission.pdf]

Reviewer Report

Title: Integrating deep mutational scanning and low-throughput mutagenesis data to predict the impact of amino acid variants

Version: Original Submission **Date:** 3/21/2023

Reviewer name: Joseph Ng

Reviewer Comments to Author:

This manuscript explored whether low-throughput alanine scanning (AS) experimental data could complement deep mutational scanning (DMS) to classify the impact of amino acid substitutions in a range of protein systems. The analysis partially confirms this hypothesis in that it only applies when the functional readout being measured in the two assays are compatible with one another.
In my opinion this is an insight that should be highlighted in a publication and therefore I believe this manuscript deserved to be published. I just wish the authors could clarify & further explore the points below better in their manuscript before recommending for acceptance:
1. In my opinion the most important bit of data curation is the classification of DMS/AS pairs as high/medium/low etc. compatible, and this is the key towards the authors' insight that assay compatibility is an important determinant of whether signals in the two datasets could be cross-matched for analysis. The criteria behind this classification are listed in Figure S2 but I feel the wording needs to be more specific. For example, in Figure S2, the authors wrote 'Both assays select for similar protein properties and under similar conditions' - what exactly does this mean? What does the authors consider to be 'similar protein properties'? I could not find more detailed explanation of this in the Methods section. The authors gave reasons in the spreadsheet in Supp. Table 1 for the labels they give to each pairs of assays, but I'm still not exactly sure what they consider to be 'similar'. Is there are more specific classification scheme which is more explicit in defining these 'similarities', e.g. by defining a scoring grid explicitly listing the different levels of 'similarities' of measurable properties, e.g. both thermal stability - score of 3; thermal stability vs protein abundance - 2; thermal stability vs cell survival - 1 (or equivalent, I think the key issue is to provide the reader with a clear guide so they can readily assess the compatibility of the datasets by themselves)?
2. I would have thought discrepancy between the DMS and AS scores to be different across different structural regions of the protein, e.g. the discrepancy would be larger in ordered region compared to disorder as the protein fold would constrain the types of amino acids tolerable within the ordered segment of the protein. Is this the case in the authors' collection of datasets? If so, does the compatibility of assays modulate this discrepancy?

Methods

Are the methods appropriate to the aims of the study, are they well described, and are necessary controls included? Choose an item.

Conclusions

Are the conclusions adequately supported by the data shown? Choose an item.

Reporting Standards

Does the manuscript adhere to the journal's guidelines on minimum standards of reporting? Choose an item.

Choose an item.

Statistics

Are you able to assess all statistics in the manuscript, including the appropriateness of statistical tests used? Choose an item.

Quality of Written English

Please indicate the quality of language in the manuscript: Choose an item.

Declaration of Competing Interests

Please complete a declaration of competing interests, considering the following questions:

- Have you in the past five years received reimbursements, fees, funding, or salary from an organisation that may in any way gain or lose financially from the publication of this manuscript, either now or in the future?
- Do you hold any stocks or shares in an organisation that may in any way gain or lose financially from the publication of this manuscript, either now or in the future?
- Do you hold or are you currently applying for any patents relating to the content of the manuscript?
- Have you received reimbursements, fees, funding, or salary from an organization that holds or has applied for patents relating to the content of the manuscript?
- Do you have any other financial competing interests?
- Do you have any non-financial competing interests in relation to this paper?

If you can answer no to all of the above, write 'I declare that I have no competing interests' below. If your reply is yes to any, please give details below.

I declare that I have no competing interests

I agree to the open peer review policy of the journal. I understand that my name will be included on my report to the authors and, if the manuscript is accepted for publication, my named report including any attachments I upload will be posted on the website along with the authors' responses. I agree for my

report to be made available under an Open Access Creative Commons CC-BY license (http://creativecommons.org/licenses/by/4.0/). I understand that any comments which I do not wish to be included in my named report can be included as confidential comments to the editors, which will not be published.

Choose an item.

To further support our reviewers, we have joined with Publons, where you can gain additional credit to further highlight your hard work (see: https://publons.com/journal/530/gigascience). On publication of this paper, your review will be automatically added to Publons, you can then choose whether or not to claim your Publons credit. I understand this statement.

Yes Choose an item.
