# Supplementary material for: Integrating deep mutational scanning and low-throughput mutagenesis data to predict the impact of amino acid variants
Source: Gigascience. 2023 Sep 18;12:giad073. doi: 10.1093/gigascience/giad073 (PMC10506130; doi:10.1093/gigascience/giad073)
Supplement: giad073_Reviewer_2_Report_Original_Submission — Leopold Parts -- 4/7/2023 Reviewed [file giad073_reviewer_2_report_original_submission.pdf]

Reviewer Report

Title: Integrating deep mutational scanning and low-throughput mutagenesis data to predict the impact of amino acid variants

Version: Original Submission **Date:** 4/7/2023

Reviewer name: Leopold Parts

Reviewer Comments to Author:

Summary
Fu et al. explore utilising low-throughput mutational fitness measurements to predict the results of high-throughput deep mutational scanning experiments. They demonstrate that adding alanine scanning results to predictive models improves performance, as long as the alanine scan used a sufficiently similar evaluation approach to a deeper experiment. The findings make intuitive sense, and will be useful for the community to internalize.
While we have several comments about the methods used, and requests to fortify the claims with more characterization, we do not expect addressing any of them will change the core findings. One can argue that direct application of AS boosted predictions is likely to be limited due to the number of scans available and the speed at which DMS experiments are now being performed, so it would also be useful to discuss the context of these results in the evolution of the field, and we make specific suggestions for this. Regardless, the presented results are a useful demonstration of a more general use case of low-throughput or partial mutagenesis data for improving fitness prediction and imputation.
Major Comments
* There are many other computational variant effect predictors beyond Envision and DeMaSk. It would be very useful to see how their prediction results compare to some others, particularly the best performing and common models that are also straightforward to download and run (e.g. EVE, ESM1v, SIFT, PolyPhen2). This would be important context to see how impactful the addition of AS data is to DeMaSk/Envision. Please run additional prediction tools for reference of absolute performance; there is no need to incorporate AS data into them.
* Several proteins have a very small number of AS residues (Figure 2), and from our reading of the methods, other residue scores are imputed with the mean AS value for that protein. (As an aside, it would be good to clarify if this average is across studies or within study). If this reading is correct, the majority of residues for each proteins will have imputed AS results (e.g. in case of PTEN, over 90%), which can be problematic for training and prediction. Please clarify if our interpretation of the imputation approach is correct, and if so, please also provide results for a model trained without imputation, on many fewer residues. If the boosting model has already implemented this, please integrate the Supplementary methods into the main methods, and reference these and the results when describing the imputation approach to avoid such concerns.
* It is not clear how significant/impactful the increases in performance are in figures 4, 5, S4, S5 & S6. Please use a reasonable analytical test, or training data randomization to evaluate the improvement against a null model.

* There are quite a few proteins with repeated DMS/AS measurements. In our experience these correlate from moderately to very highly. Including multiple highly correlated studies could lead to pseudo-replication and biasing the model performance results. Please present a version of the results where the repeats are averaged first to test whether that bias exists.

Minor Comments [suggestions only; no analyses required from us]

* A short discussion about the number of available alanine scans, particularly for proteins without DMS results, would help put the work in context. For example, it would be good to know how many proteins would benefit from improved de-novo predictions (e.g. no DMS data) and how many could have improved imputation (incomplete DMS data). Similarly the rate and cost of DMS data generation is important to understand the utility of their results. I think a short discussion of how useful models of this sort are in practice now and in future would be helpful to the reader. This seems most natural as part of the end of the discussion, but could also fit in the introduction.
* Figure 2 is missing y axis label. We also softly suggest log scale axis, to not obscure the degree to which some proteins have more residues covered and the proportion of residues covered by AS.
* Figure 3 includes DMS/AS study pairs with at least three alanine substitutions to compare - we think this is a low cut-off, particularly with the regularisation applied. I think something like 10+ would be more informative.
* I think their cross-validation scheme leaves out an entire protein at a time, as opposed to one study each iteration. I agree this is the better way to do it. However, I initially read it as the latter, which would lead to leakage between train/validation data since the same residue would be included in both if a protein had multiple datasets. It might be useful to be more explicit to prevent other readers doing the same.
* L231 In the discussion they mention fitting a model only using studies with a minimum DMS/AS correlation. This occurred to me as well while reading the relevant part of the results. Is there a good reason not to do this? It doesn't seem like a large amount of work and conceptually seems a good way to assess a model that says what a DMS might look like is it had the same selection criteria as a given AS.
* L154 Similarly, a correlation cut-off as well as choosing the most corelated study seems like it would be a fairer comparison in figure 5. Just because an AS is the most correlated doesn't necessarily mean it is well correlated.
* It would be interesting to see if the improvement results in figure 7 correlate with substitution matrices (e.g. Blosum) or DMS variant fitness correlations (e.g. correlation between A and C, A and D, etc.). Intuitively it feels like they should.
* It would be nice to label panels in figure 7.
* It also seems notable that predicting alanine substitutions is not the most improved - a brief comment on why would be interesting.
* The AS model adds 2x20 parameters to the model for encoding, which is a lot if CCR5 is held out, as there are only a few hundred total independent residues evaluated. While the performance on held out proteins is a good standard, it would be interesting to evaluate the increase from model selection perspective (BIC/AIC or similar) if possible.
* L217 The statement doesn't seem logical to me - if such advanced imputation methods were available surely they would be better used to impute all substitutions than just model alanine then use linear regression to model the rest?

* L331-332 The formula used for regularising Spearman's rho makes sense, and can likely be interpreted as a regularizing prior, but we found it hard to understand its provenance and meaning from the reference. A sentence on its content (not just describing that it shrinks estimates) and a more specific reference would be useful for interested readers like ourselves.
* L364 It says correlation results were dropped when only one residue was available whereas in figure legends it says results with less than three residues were dropped. Notwithstanding thinking three is maybe too low a cutoff, these should be consistent or clarified slightly if I've misunderstood the meaning.
* It would be nice to have a bit more comment on the purpose of the final supplementary section (Replacing AS data with DMS scores of alanine substitutions) - if you have DMS alanine results it seems likely you will have the other measurements anyway.

Methods

Are the methods appropriate to the aims of the study, are they well described, and are necessary controls included? Choose an item.

Conclusions

Are the conclusions adequately supported by the data shown? Choose an item.

Reporting Standards

Does the manuscript adhere to the journal's guidelines on [minimum standards of reporting?]() Choose an item.

Choose an item.

Statistics

Are you able to assess all statistics in the manuscript, including the appropriateness of statistical tests used? Choose an item.

Quality of Written English

Please indicate the quality of language in the manuscript: Choose an item.

Declaration of Competing Interests

Please complete a declaration of competing interests, considering the following questions:

- Have you in the past five years received reimbursements, fees, funding, or salary from an organisation that may in any way gain or lose financially from the publication of this manuscript, either now or in the future?

- Do you hold any stocks or shares in an organisation that may in any way gain or lose financially from the publication of this manuscript, either now or in the future?
- Do you hold or are you currently applying for any patents relating to the content of the manuscript?
- Have you received reimbursements, fees, funding, or salary from an organization that holds or has applied for patents relating to the content of the manuscript?
- Do you have any other financial competing interests?
- Do you have any non-financial competing interests in relation to this paper?

If you can answer no to all of the above, write 'I declare that I have no competing interests' below. If your reply is yes to any, please give details below.

I declare that we have no competing interests

I agree to the open peer review policy of the journal. I understand that my name will be included on my report to the authors and, if the manuscript is accepted for publication, my named report including any attachments I upload will be posted on the website along with the authors' responses. I agree for my report to be made available under an Open Access Creative Commons CC-BY license (http://creativecommons.org/licenses/by/4.0/). I understand that any comments which I do not wish to be included in my named report can be included as confidential comments to the editors, which will not be published.

Choose an item.

To further support our reviewers, we have joined with Publons, where you can gain additional credit to further highlight your hard work (see: https://publons.com/journal/530/gigascience). On publication of this paper, your review will be automatically added to Publons, you can then choose whether or not to claim your Publons credit. I understand this statement.

Yes Choose an item.
